# Accumulated precursors of specific GPI-anchored proteins upregulate GPI biosynthesis with ARV1

**DOI:** 10.1083/jcb.202208159

**Published:** 2023-02-24

**Authors:** Yi-Shi Liu, Yicheng Wang, Xiaoman Zhou, Linpei Zhang, Ganglong Yang, Xiao-Dong Gao, Yoshiko Murakami, Morihisa Fujita, Taroh Kinoshita

**Affiliations:** 1 Key Laboratory of Carbohydrate Chemistry and Biotechnology, Ministry of Education, School of Biotechnology, Jiangnan University, Wuxi, China; 2 Research Institute for Microbial Diseases, Osaka University, Suita, Japan; 3 WPI Immunology Frontier Research Center, Osaka University, Suita, Japan; 4 Institute for Glyco-Core Research, Gifu University, Gifu, Japan; 5 Center for Infectious Disease Education and Research, Osaka University, Suita, Japan

## Abstract

We previously reported that glycosylphosphatidylinositol (GPI) biosynthesis is upregulated when endoplasmic reticulum–associated degradation (ERAD) is defective; however, the underlying mechanistic basis remains unclear. Based on a genome-wide CRISPR–Cas9 screen, we show that a widely expressed GPI-anchored protein CD55 precursor and ER-resident ARV1 are involved in upregulation of GPI biosynthesis under ERAD-deficient conditions. In cells defective in GPI transamidase, GPI-anchored protein precursors fail to obtain GPI, with the remaining uncleaved GPI-attachment signal at the C-termini. We show that ERAD deficiency causes accumulation of the CD55 precursor, which in turn upregulates GPI biosynthesis, where the GPI-attachment signal peptide is the active element. Among the 31 GPI-anchored proteins tested, only the GPI-attachment signal peptides of CD55, CD48, and PLET1 enhance GPI biosynthesis. ARV1 is prerequisite for the GPI upregulation by CD55 precursor. Our data indicate that GPI biosynthesis is balanced to need by ARV1 and precursors of specific GPI-anchored proteins.

## Teaser

Liu et al. show that precursors of specific GPI-anchored proteins, such as CD55, function with an ER-resident lipid homeostasis regulator ARV1 to upregulate GPI biosynthesis in the ER. This may be an important mechanism to increase GPI when needed.

## Introduction

The ER is a specialized eukaryotic organelle for protein synthesis and processing, lipid synthesis, and calcium storage and release (Braakman and Hebert, 2013; Clapham, 2007; Schwarz and Blower, 2016). Various posttranslational modifications of proteins that reside in or traverse secretory pathways also occur in the ER. For example, N-linked glycans are attached in the ER for efficient folding and proper functions (Braakman and Hebert, 2013; Helenius and Aebi, 2004; Sicari et al., 2019). Glycosylphosphatidylinositol (GPI) anchoring is one of the conserved posttranslational modifications in eukaryotic cells. More than 150 human proteins have been confirmed as GPI-anchored proteins (GPI-APs; Kinoshita, 2020; Liu and Fujita, 2020). GPIs confer characteristic features to the modified proteins that are essential for embryogenesis, neurogenesis, immune response, and fertilization (Nozaki et al., 1999; Um and Ko, 2017; Wang et al., 2013; Watanabe et al., 2017). GPI-APs are biosynthesized in the ER and transported to the cell surface through the Golgi apparatus. The common backbone of GPI, EtNP-6Manα-2Manα-6Manα-4GlcNα-6inositol-phospholipid (where EtNP, Man, and GlcN are ethanolamine phosphate, mannose, and glucosamine, respectively), is generated by sequential additions of components to the phosphatidylinositol (PI) moiety (Orlean and Menon, 2007; Pittet and Conzelmann, 2007). Once assembled, GPI is attached to the proteins by GPI transamidase and then undergoes structural remodeling in both glycan and lipid portions (Fujita et al., 2009; Kinoshita and Fujita, 2016; Liu et al., 2021). The structural variations of GPI anchors are introduced by modification of the core by side chains. The first, α1,6-linked mannose, is often modified by β1,4-linked N-acetylgalactosamine (GalNAc) by PGAP4 (Hirata et al., 2018). The GalNAc side chain can be further modified by β1,3 galactose (Gal) and then with sialic acid (Hirata et al., 2018; Kobayashi et al., 2020). Sialic acid in the GPI side chain of prion proteins, α2-3-linked N-acetylneuraminic acid (Kobayashi et al., 2020), affects the process of prion disease progression (Kobayashi et al., 2020); however, the biological roles of the GPI side chains in various GPI-APs are largely unknown.

**Figure 1.**
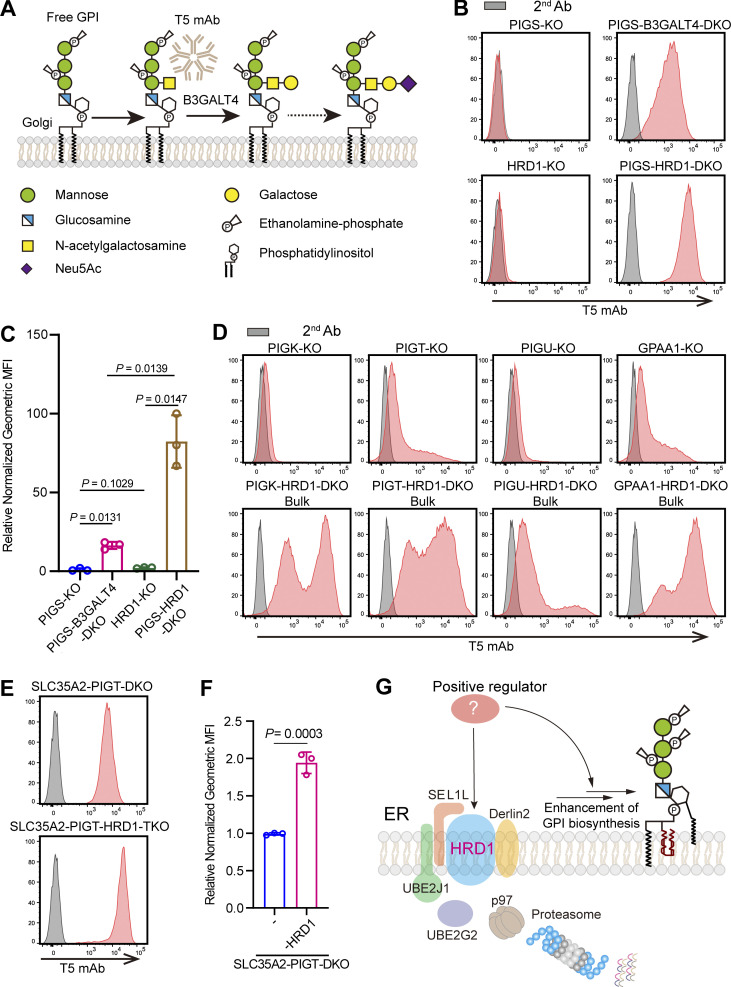
**Defect in ERAD-L pathway upregulates GPI biosynthesis. (A)** Schematic of free GPI side chain modifications with GalNAc, Gal, and sialic acid. B3GALT4 is responsible for transferring Gal to the β1,4-linked GalNAc side chain of GPI. T5 mAb binds to free GPI when GalNAc is not modified by Gal. Monosaccharide symbols are drawn according to the symbol nomenclature for glycans. **(B and C)** Flow cytometry results for PIGS-KO, PIGS-B3GALT4-DKO, HRD1-KO, and PIGS-HRD1-DKO cells stained with T5 mAb that recognizes free GPI with the GalNAc side chain. Normalized geometric MFI is determined for each cell type. Relative normalized geometric MFI (a value of PIGS-KO cells being 1.0) are displayed as the mean ± SD from three independent experiments with P values (one-way ANOVA followed by Dunnett’s multiple comparisons test). **(D)** Flow cytometry results for pooled cultures of GPI transamidase subunit-defective cells, PIGK-KO, PIGT-KO, PIGU-KO, and GPAA1-KO after knocking out HRD1. Cells were stained with T5 mAb. **(E and F)** Flow cytometry analysis of SLC35A2-PIGT-DKO and SLC35A2-PIGT-HRD1-TKO cells stained with the T5 mAb. Relative normalized geometric MFIs of these cells are displayed as the mean ± SD from three independent experiments with P values (unpaired Student’s *t* test). **(G)** Schematic of the ERAD-L pathway negatively regulating GPI biosynthesis by degrading unknown factors. Validated genes are shown in the figure.

The initial GPI identified is a type of glycolipid that anchors various proteins on the cell membrane. In certain parasites, nonprotein-linked GPIs (free GPIs) exist on the membrane (Sharma et al., 1983; Striepen et al., 1997; Tomavo et al., 1994). In addition, recent works have indicated that free GPIs are also found in some tissues and cell types in mammals (Baumann et al., 2000; Duval et al., 2021; Singh et al., 1996; Wang et al., 2019). The T5-4E10 monoclonal antibody (T5 mAb), originally established against free GPI from the parasite *Toxoplasma gondii*, recognizes a GalNAc side chain only when GalNAc is a nonreducing terminal residue (Striepen et al., 1997; Tomavo et al., 1994). We found that the T5 mAb also recognizes mammalian free GPIs with a GalNAc side chain and that when the GalNAc side chain is modified by Gal, the T5 mAb no longer binds (Hirata et al., 2018; Fig. 1 A). We also found that when GPI transamidase is defective, GPIs are expressed as free GPIs on the cell surface (Wang et al., 2019). Nevertheless, in HEK293 cells, GPI transamidase defects caused by knockout (KO) of phosphatidylinositol glycan class S (PIGS), one of the GPI transamidase components, did not induce positive T5 mAb staining (Wang et al., 2020). This was caused by modification of all the GalNAc side chains by Gal. Indeed, strong T5 mAb staining appeared when Gal transfer was blocked by KO of SLC35A2 encoding UDP-Gal transporter necessary for galactosyltransferases (Wang et al., 2022). This phenomenon allowed us to perform genome-wide CRISPR screening for genes involved in GPI side-chain galactosylation. Among genes with KO resulting in positive T5 mAb staining of PIGS-KO HEK293 cells, we identified B3GALT4, which transfers Gal to the GalNAc side chain (Fig. 1 A; Wang et al., 2020). In the same screening, we found that KO of ER-associated degradation (ERAD) pathway genes, such as HRD1, UBE2J1, and UBE2G2, caused positive T5 mAb staining and demonstrated that GPI biosynthesis was upregulated by two- to several-fold in ERAD-defective PIGS-KO cells, so that the amount of free GPI surpassed the galactosylation capacity of the cells, resulting in positive staining of the cell by T5 mAb (Wang et al., 2020).

ERAD plays a critical role in proteostasis under normal or stress conditions. Proteins that fail to fold or assemble are recognized by chaperones and lectins, transferred to membrane-integral adaptors, and retrotranslocated through E3 ubiquitin ligases, followed by ubiquitination for cytosolic proteasomal degradation (Baldridge and Rapoport, 2016; Lemberg and Strisovsky, 2021; Neal et al., 2018; Sun and Brodsky, 2019). The ERAD system consists of several parallel pathways, ERAD-L, ERAD-M, and ERAD-C, handling the substrate proteins in the ER lumen, ER membrane, and ER cytoplasmic surface, respectively (Habeck et al., 2015; Leto et al., 2019; Neal et al., 2018; Shi et al., 2019; Wu et al., 2020). Various E3 ligases, including HRD1, GP78, TRC8, and MARCH6, are utilized in these pathways (Lemberg and Strisovsky, 2021; Neal et al., 2018). HRD1 is the best characterized and the major retrotranslocation component for luminal ERAD-L substrates (Peterson et al., 2019; Schoebel et al., 2017). Both in vitro and in vivo studies have revealed that multimembrane spanning HRD1 serves as a channel for luminal ERAD-L substrates. HRD1 recognizes a variety of substrates to maintain ER homeostasis (Peterson et al., 2019; Schoebel et al., 2017; Vasic et al., 2020).

Here, to identify factors involved in GPI biosynthesis upregulation under ERAD-L defective conditions, we performed genome-wide CRISPR screening and identified CD55 (a widely expressed GPI-AP also known as complement decay-accelerating factor) and ARV1. We show that a precursor of CD55 is an ERAD-L substrate and acts as a positive regulator of GPI biosynthesis. We further show that ARV1, which is known to be involved in lipid homeostasis (Georgiev et al., 2013) and also implicated in GPI biosynthesis in *Saccharomyces cerevisiae* (Kajiwara et al., 2008; Okai et al., 2020) and humans (Davids et al., 2020; Salian et al., 2021), is required for CD55 precursor to upregulate GPI biosynthesis. Our results suggest that GPI biosynthesis is regulated on demand by the status of GPI precursor proteins.

## Results

### Biosynthesis of GPI is upregulated in ERAD-L–deficient cells

When GPI transamidase that attaches GPI to proteins is inactive, non-protein linked GPIs remain as free GPIs and are transported from the ER to the cell surface (Wang et al., 2019). Like GPI-AP, free GPI is modified by GalNAc side chain in the Golgi apparatus (Fig. 1 A). In cells expressing B3GALT4, GalNAc side chains are modified by Gal (Wang et al., 2020). T5 mAb binds to free GPI only when side chain GalNAc is unmodified and T5 mAb epitope is lost by Gal modification (Fig. 1 A). PIGS-KO HEK293 cells are defective in GPI transamidase, nevertheless are T5 mAb staining-negative, whereas PIGS-B3GALT4-double KO (DKO) HEK293 cells are T5 mAb staining-positive (Fig. 1 B), indicating that all GalNAc side chains are modified by Gal on PIGS-KO HEK293 cells. PIGS-HRD1-DKO cells defective in ERAD-L, as well as GPI transamidase were strongly stained with the T5 mAb, the staining intensity being approximately fourfold that of PIGS-B3GALT4-DKO cells (Fig. 1, B and C). The results suggest that the fourfold or more increased amount of free GPI surpassed the Gal-addition capacity of B3GALT4 in ERAD-L–defective cells. GPI transamidase consists of five subunits: PIGS, PIGK, PIGT, PIGU, and GPAA1. To check whether this increase in GPI under ERAD-L–defective conditions was PIGS KO specific or common among GPI transamidase defects, we knocked out the HRD1 gene in GPI transamidase gene-deficient HEK293 cells and analyzed free GPI levels on the cell surface in bulk populations. Knocking out HRD1 in PIGK-, PIGT-, PIGU-, and GPAA1-KO cells increased T5 mAb staining, as observed in PIGS-HRD1-DKO cells (Fig. 1 D), indicating that defects in ERAD-L generally increase free GPIs in cells defective in GPI transamidase. We also knocked out HRD1 in SLC35A2 and PIGT DKO (SLC35A2-PIGT-DKO) HEK293 cells. HRD1 KO in SLC35A2-PIGT-DKO cells increased T5 mAb staining levels twofold (Fig. 1, E and F). These results indicated that the ERAD-L pathway suppressed GPI biosynthesis. We hypothesized that there must be positive regulator(s) of GPI biosynthesis, which is controlled by the ERAD-L pathway (Fig. 1 G).

### Identification of positive regulators of GPI biosynthesis

**Figure 2.**
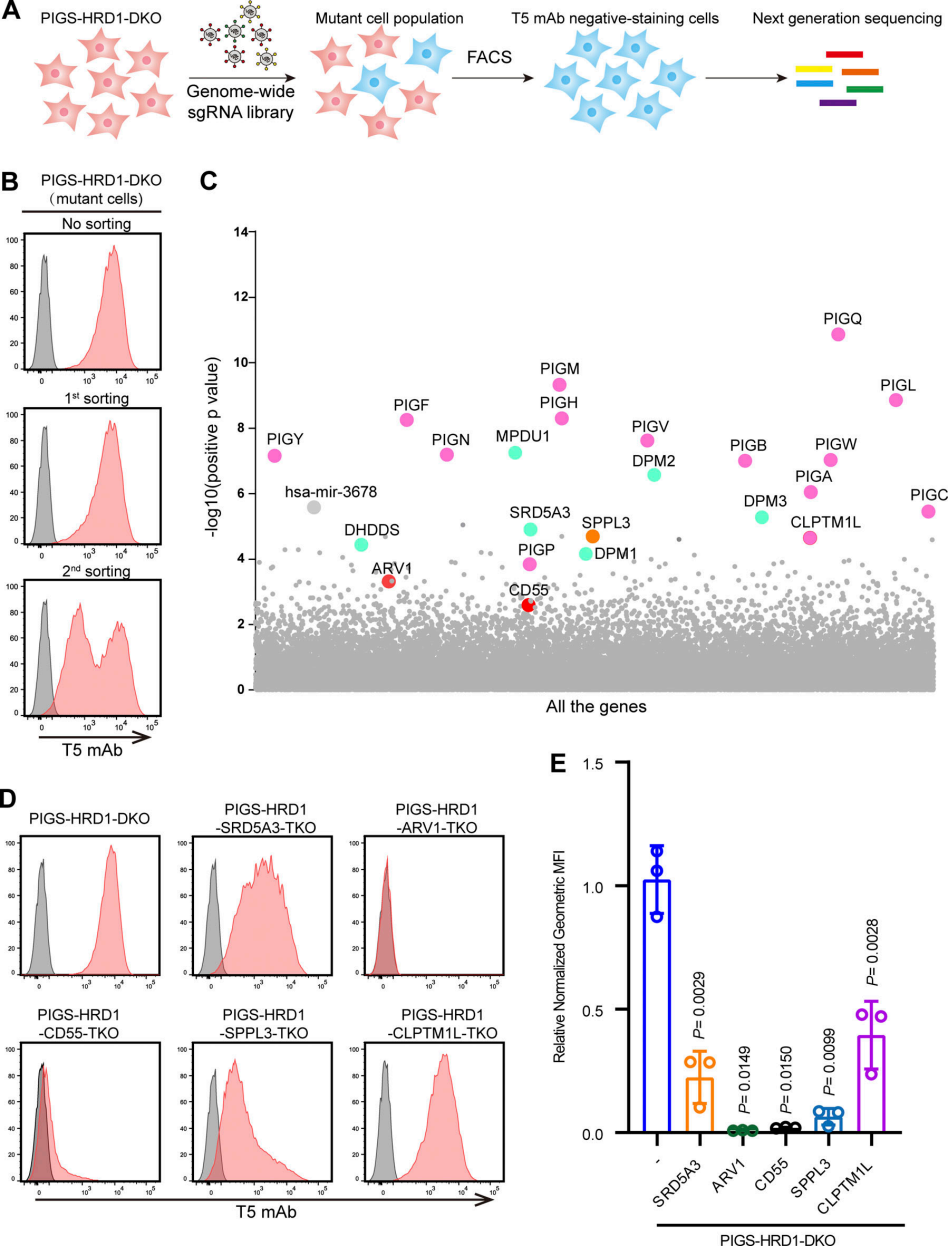
**CRISPR–Cas9 pooled screening to identify regulators of GPI biosynthesis. (A)** Strategy for a FACS-based genome-wide CRISPR screen in PIGS-HRD1-DKO cells. **(B)** Flow cytometry results for parental cells, first sorting, and second sorting of PIGS-HRD1-DKO cells stained with T5 mAb. **(C)** Scatter plot showing genes corresponding to gRNAs that were significantly enriched in second sorted PIGS-HRD1-DKO cells using MAGeCK. The pink bubble denotes PIG genes, the blue bubble denotes DPM-related and Dol-P-related genes, the orange bubble denotes SPPL3, and the red bubble denotes functionally unknown genes in GPI biosynthesis. See Data S1 for the entire data. **(D and E)** Flow cytometry analysis of the KO of top-ranking five genes identified using our CRISPR screen in PIGS-HRD1-DKO cells. Cells were stained with T5 mAb. Relative normalized geometric MFI of PIGS-HRD1-DKO is set 1.0 and those of five gene KO cells are displayed as the mean ± SD from three independent experiments with P values (one-way ANOVA followed by Dunnett’s multiple comparisons test).

**Figure S1.**
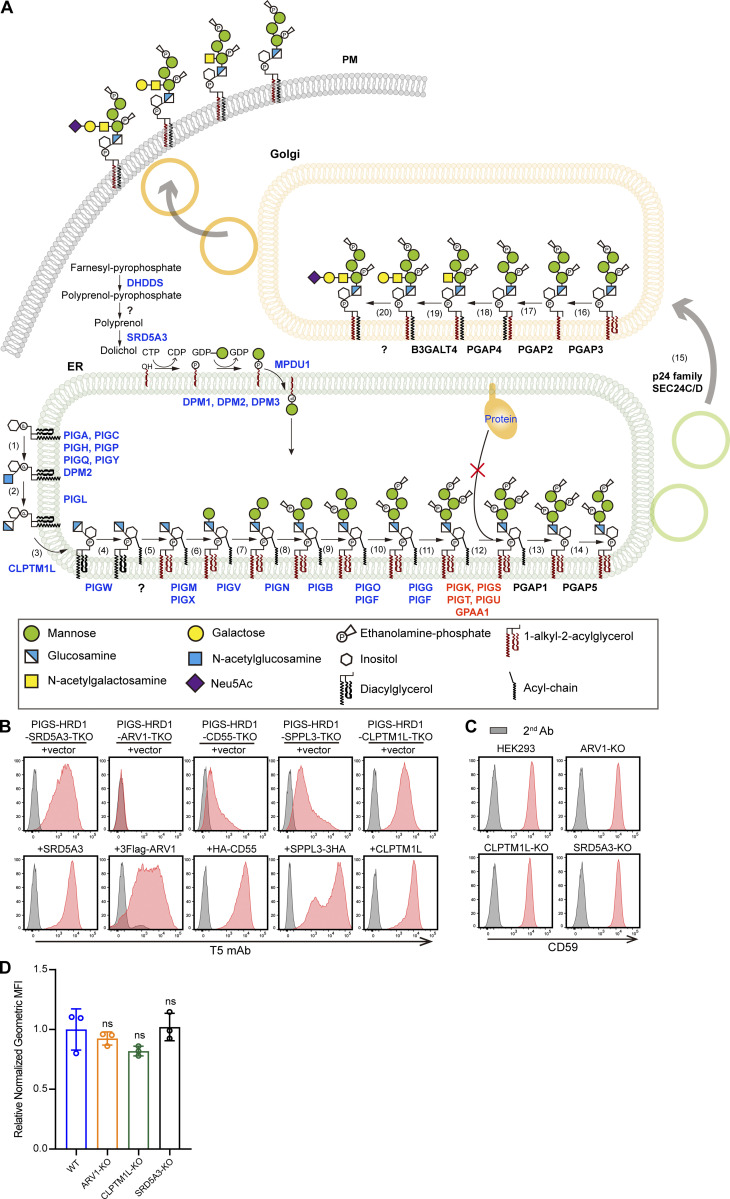
**CRISPR–Cas9 pooled screening to identify regulators of GPI biosynthesis. (A)** Graphic depiction of the GPI biosynthesis pathway in mammalian cells. Genes in blue were significant by MAGeCK analysis, genes in red were complexes of GPI transamidases, and genes in black showed no enrichment. **(B)** Validation of the screening results via the expression of related genes in KO cells. Cells were stained with T5 mAb and analyzed by FACS. **(C and** (**D)** Flow cytometry analysis of the KO of ARV1, CLPTM1L, and SRD5A3 in HEK293 WT cells. Cells were stained with anti-CD59 and quantified. Relative normalized geometric MFI in KO cells is compared with WT cells and displayed as the mean ± SD from three independent experiments (one-way ANOVA followed by Dunnett’s multiple comparisons test).

To identify genes that upregulate GPI biosynthesis when ERAD-L is inactive, we performed a genome-wide CRISPR–Cas9 screen in PIGS-HRD1-DKO HEK293 cells. PIGS-HRD1-DKO cells are positively stained by T5 mAb, whereas PIGS-KO cells are negative for T5 mAb staining, allowing the selection of mutant cells defective in upregulation of GPI biosynthesis. PIGS-HRD1-DKO cells were transduced with lentivirus capable of expressing a human genome-scale CRISPR KO gRNA library (GeCKO v2) and Cas9 enzyme. We then cultured the cells for 2 wk, and cells negatively stained with the T5 mAb were enriched by cell sorting (Fig. 2 A). After two rounds of cell sorting and culture, more than half of the cells became very weakly positive or negative for T5 mAb staining (Fig. 2 B). Genomic DNA was extracted from cells, and the integrated gRNA sequences amplified by PCR were analyzed by deep sequencing. The enrichment of gRNAs was determined by model-based analysis of the genome-wide CRISPR–Cas9 KO (MAGeCK) method (Sanjana et al., 2014). The screening enriched for genes required for GPI biosynthesis (PIG genes) and dolichol-phosphate mannose (DPM) biosynthesis (DPM1, DPM2, DPM3, SRD54A3, and dehydrodolichyl diphosphate synthase), as expected (Fig. 2 C and Fig. S1 A). CLPTM1L (cleft lip and palate transmembrane protein 1-like protein) was highly enriched in the screening. We recently reported that CLPTM1L is a lipid scramblase involved in GPI biosynthesis (Wang et al., 2022). SPPL3, which encodes a membrane-bound aspartic protease that cleaves type-II membrane proteins at the Golgi, was also enriched in the screening. It has been reported that SPPL3 affects the expression of a GPI-AP, CD59, through the regulation of glycosphingolipid biosynthesis (Davis et al., 2015; Kawaguchi et al., 2021). Considering that the screen would identify substrates of the ERAD-L pathway, we expanded our attention to other genes encoding ER-localized proteins. Two GPI-related genes, ARV1 and CD55, although they were not highly ranked (ranking #87 and #279, respectively), attracted our attention (Fig. 2 C). To validate the screening results, we chose and knocked out five genes, SRD5A3, ARV1, CD55, SPPL3, and CLPTM1L, in PIGS-HRD1-DKO cells. The KO cells were analyzed by staining with T5 mAb. All KO cells showed a clear decrease in T5 mAb staining compared with parental PIGS-HRD1-DKO cells. In particular, T5 mAb staining decreased to nearly background levels after knocking out ARV1 or CD55 (Fig. 2, D and E). Decreased T5 mAb staining was restored by transfection of relevant cDNAs (Fig. S1 B). When SRD5A3, ARV1, SPPL3, and CLPTM1L were knocked out in HEK293 WT cells, the surface CD59, a GPI-AP, was only slightly decreased or unchanged compared with parental cells (Fig. S1, C and D). These genes were not identified from previous genetic screens using cell surface GPI-APs as reporters. These data suggested that free GPI would be a more sensitive reporter, enabling us to identify more genes involved in GPI biosynthesis.

### CD55 precursor upregulates GPI biosynthesis

**Figure 3.**
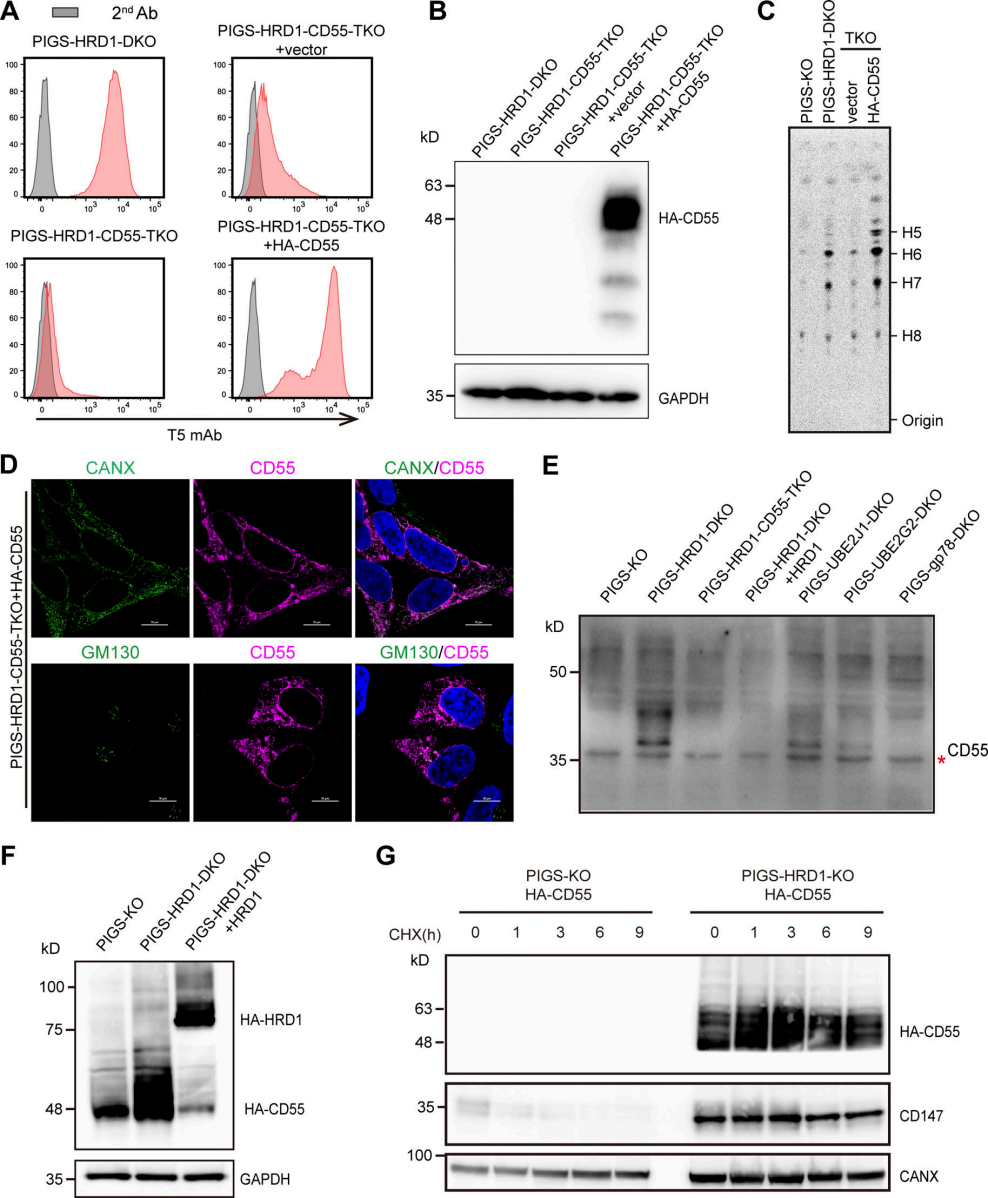
**The precursor of CD55 regulates GPI biosynthesis and is a substrate of the ERAD-L pathway. (A)** PIGS-HRD1-CD55-TKO cells were stably transfected with EV or HA-tagged CD55. Surface expression of free GPI was stained with T5 mAb and analyzed by flow cytometry. **(B)** Cell lysates prepared from the cells used in A were analyzed by WB. Expression of HA-CD55 was detected. GAPDH was used as a loading control. **(C)** Thin-layer chromatography/phosphor imaging analysis of GPI biosynthesis in cells metabolically labeled with [2-^3^H] mannose. Data shown are representative one of three repeated experiments. **(D)** Fluorescence images of PIGS-HRD1-CD55-TKO cells stably expressing HA-tagged CD55. CANX, a marker of ER; GM130, a marker of cis-Golgi. Scale bar, 10 μm. **(E)** WBs of endogenous CD55. Lysates of PIGS-KO, PIGS-HRD1-DKO, PIGS-HRD1-CD55-TKO, PIGS-HRD1-DKO cells stably expressing HA-tagged HRD1, PIGS-UBE2J1-DKO, PIGS-UBE2G2-DKO, and PIGS-GP78-DKO cells were analyzed. The red asterisk indicates a nonspecific band. **(F)** Cell lysates prepared from PIGS-KO, PIGS-HRD1-DKO, and PIGS-HRD1-DKO cells stably expressing HA-tagged HRD1 transiently transfected with HA-tagged CD55 were analyzed by WB. Expression of HA-CD55 was detected. GAPDH was used as a loading control. **(G)** PIGS-KO and PIGS-HRD1-DKO cells stably expressing HA-tagged CD55 were treated with 1 μg ml^−1^ CHX for 1, 3, 6, and 9 h. Cell lysates prepared from CHX-treated cells were analyzed by Western blotting. The protein amount of HA-CD55 was detected. CD147, a substrate of HRD1, was used as a positive control, and CANX was used as a loading control. Source data are available for this figure: SourceData F3.

Among the candidate genes identified in the screening, CD55 encodes a GPI-anchored complement regulatory factor that is ubiquitously expressed in the human body. To understand the cellular mechanisms by which knocking out the GPI-AP gene causes a decrease in GPI biosynthesis, we first generated a PIGS-HRD1-CD55-triple KO (TKO) cell line, in which the expression of surface-free GPIs decreased to the background level (Fig. 2 D and Fig. 3 A). When we stably expressed HA-tagged CD55 in the TKO cells, the amounts of surface-free GPIs were restored or were even higher (geometric mean fluorescence intensity [MFI] 8594) than those in PIGS-HRD1-DKO cells (geometric MFI 6515; Fig. 3, A and B). Transfection of an empty vector (EV) into PIGS-HRD1-CD55-TKO sometimes caused weakly positive T5 staining for an unknown reason (Fig. 3 A, bottom left vs. top right). To test whether the CD55-dependent increase in free GPI on the cell surface was due to GPI biosynthesis, we metabolically labeled cells with [2-^3^H] mannose and analyzed radiolabeled GPI mannolipids by thin-layer chromatography. The GPI precursors (H5, H6, H7, and H8) were decreased by knocking out CD55, whereas they were greatly increased by overexpression of CD55, suggesting that GPI biosynthesis was upregulated by expression of CD55 in PIGS-HRD1-CD55-TKO cells (Fig. 3 C).

In normal cells, CD55 protein is synthesized and attached to GPI catalyzed by GPI transamidase in the ER, whereas in PIGS-KO cells, CD55 fails to link to GPI and remains in a precursor form that possesses a GPI attachment signal peptide at the C-terminus. We next analyzed the localization of CD55 in PIGS-HRD1-DKO HEK293 cells by immunofluorescence (IF). The precursor of CD55 was mainly colocalized with an ER marker, calnexin (CANX), but only weakly with a Golgi marker, GM130 (Fig. 3 D), suggesting that the majority of CD55 precursor proteins remain in the ER. GPI-AP precursor proteins that fail to anchor GPI are thought to be degraded through ERAD pathways (Ashok and Hegde, 2008; Sikorska et al., 2016). To validate whether the CD55 precursor was an ERAD substrate, we analyzed endogenous CD55 levels. Western blots (WBs) revealed specific endogenous immature CD55 bands in ERAD-L–defective PIGS-HRD1-DKO, PIGS-UBE2J1-DKO, and PIGS-UBE2G2-DKO cells (Fig. 3 E). In contrast, bands did not appear in PIGS-KO, HRD1-rescued PIGS-HRD1-DKO, or PIGS-HRD1-CD55-TKO cells. The protein was also undetectable in PIGS-GP78-DKO cells. The E3 ligase GP78 plays a role in another ERAD pathway for membrane proteins, ERAD-M (Fig. 3 E), suggesting that the CD55 precursor is mainly degraded by the HRD1-dependent ERAD-L pathway. We further transiently overexpressed HA-CD55 in PIGS-KO and PIGS-HRD1-DKO cells. The amount of HA-CD55 was much higher when HRD1 was defective (Fig. 3 F). To determine the stability of HA-CD55, we treated PIGS-KO and PIGS-HRD1-KO cells with cycloheximide (CHX) to stop new synthesis of proteins. In PIGS-KO cells, CD147, one of the classic ERAD-L substrates, was degraded in a time-dependent manner. HA-CD55 was undetectable under similar conditions. However, both CD147 and HA-CD55 proteins became stable in PIGS-HRD1-DKO cells (Fig. 3 G). These results indicated that the CD55 precursor was a substrate of the HRD1-dependent ERAD pathway and that its accumulation in PIGS-HRD1-DKO cells caused the upregulation of GPI biosynthesis.

**Figure S2.**
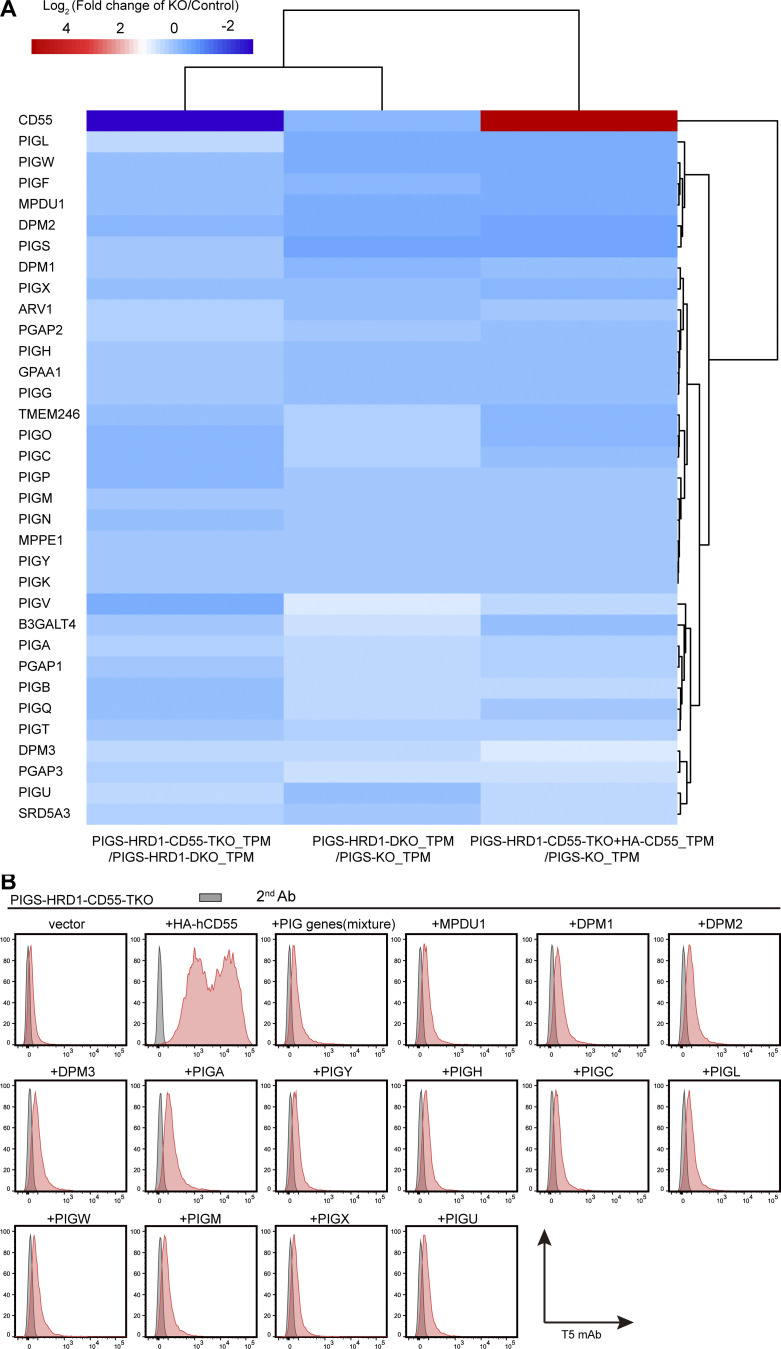
**Analysis of PIG gene expression and protein stability in PIGS-HRD1-CD55-TKO cells. (A)** RNA-seq results from PIGS-KO, PIGS-HRD1-DKO, PIGS-HRD1-CD55-TKO, and PIGS-HRD1-CD55-TKO cells stably overexpressing HA-CD55. Genes related to GPI biosynthesis are listed. **(B)** Flow cytometry analysis of PIGS-HRD1-CD55-TKO cells overexpressing mixed or individual PIG genes. Cells were stained with T5 mAb.

To understand the mechanistic basis of GPI biosynthesis upregulation by the CD55 precursor, we performed RNA sequencing (RNA-seq) for parental PIGS-HRD1-DKO, PIGS-HRD1-CD55-TKO, and HA-CD55–transfected PIGS-HRD1-CD55-TKO cells. Total RNA was extracted and analyzed. The expression profile of GPI biosynthesis-related genes was not significantly affected by CD55 (Fig. S2 A). To directly test whether PIG genes were involved in the GPI biosynthesis upregulation, we transiently transfected PIG genes or a mixture of DPM-related genes into PIGS-HRD1-CD55-TKO cells, but no effects on these transfected cells were found (Fig. S2 B). Together, these findings suggested that CD55 did not regulate GPI biosynthesis through changing the levels of PIG gene-encoded proteins.

### Some GPI attachment signal peptides regulate GPI biosynthesis

**Figure 4.**
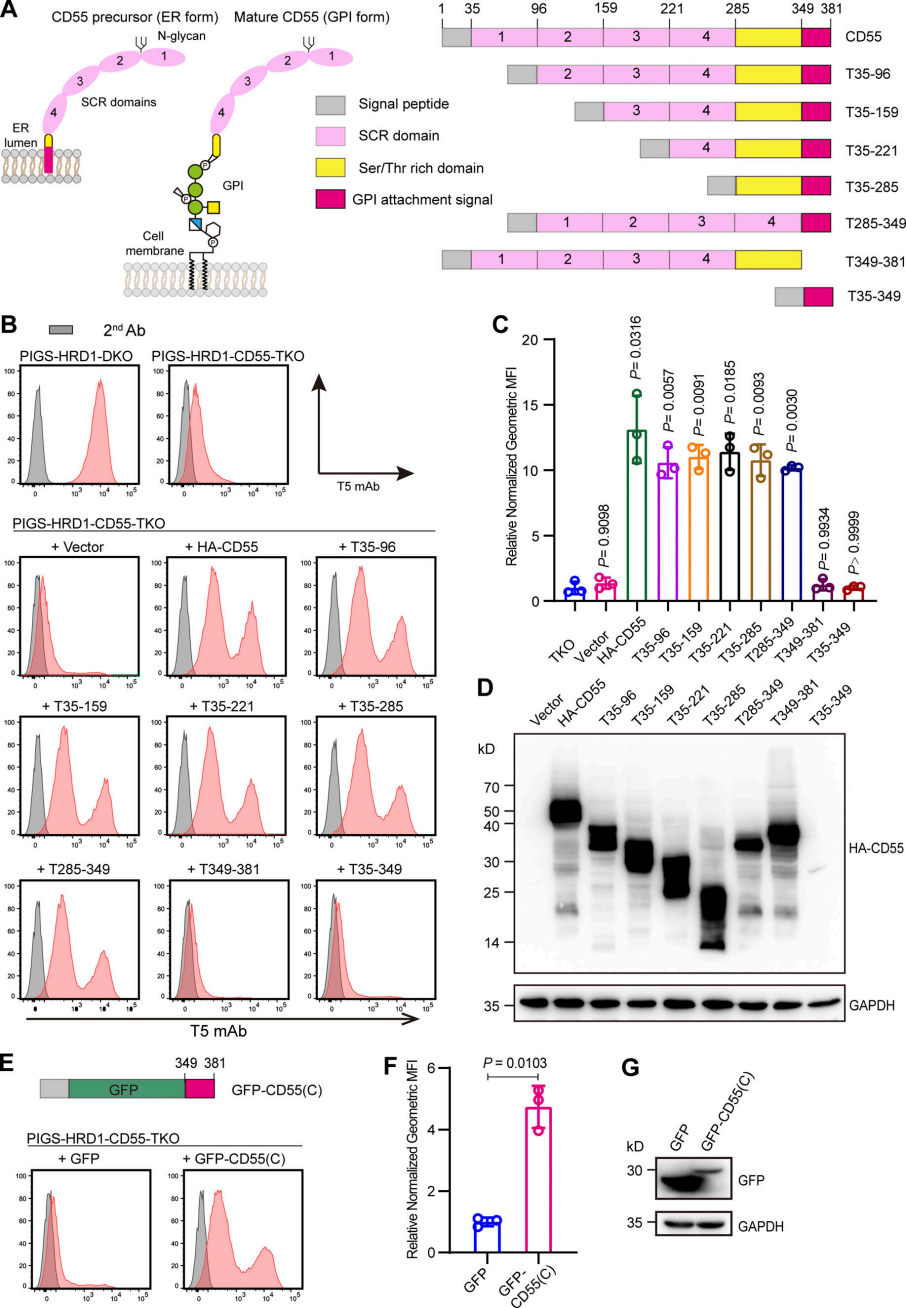
**The GPI attachment signal peptide of CD55 regulates GPI biosynthesis. (A)** Left: Schematic structure of the CD55 precursor (ER form) localized on the ER membrane, and mature CD55 (GPI form) localized on the cell surface. Right: Graphical depiction of the assay designs for truncated CD55 construction. Gray pane: Signal peptide; pink pane: SCR domain; yellow pane: Ser/Thr rich domain; red pane: GPI attachment signal. **(B and C)** Flow cytometry analysis of PIGS-HRD1-CD55-TKO cells transiently expressing plasmids with different truncated CD55 variants. Cells were stained with T5 mAb. Relative normalized geometric MFIs of PIGS-HRD1-CD55-TKO cells transiently expressing EV, HA-tagged CD55, or different truncated CD55 variants are displayed as the mean ± SD from three independent experiments with P values (one-way ANOVA followed by Dunnett’s multiple comparisons test). **(D)** Cell lysates prepared from the cells used in B were analyzed by WB. Expression of truncated CD55 variants was detected using HA antibody. GAPDH was used as a loading control. **(E**–**G)** The GPI attachment signal peptide of CD55 was fused with GFP [GFP-CD55(C)] and transiently expressed in PIGS-HRD1-CD55-TKO cells. GFP-expressing plasmid was used as negative control. Cells were stained with T5 mAb and analyzed by flow cytometry (E). The relative normalized geometric MFI of cells transfected with GFP was set to 1 and that of GFP-CD55(C)-transfected cells was displayed as the mean ± SD of three independent experiments with P value (unpaired Student’s *t* test; F). Cell lysates prepared from the cells used in E were analyzed by WB (G). The expression of fused proteins was detected by GFP antibody. GAPDH was used as a loading control. Source data are available for this figure: SourceData F4.

Mature CD55 (GPI-anchored form) contains four short consensus repeats (SCRs), one N-linked glycan between SCR 1 and 2, and a membrane-proximal O-linked polysaccharide rich region (S/T region; Coyne et al., 1992; He et al., 2002; White et al., 2004; Fig. 4 A). The CD55 precursor (ER form) is synthesized and translocated into the ER lumen through an N-terminal signal peptide and probably localized in the ER membrane by the hydrophobic region in the C-terminal GPI attachment signal peptide before GPI attachment (Fig. 4 A). To determine which part of CD55 is critical for the regulation of GPI biosynthesis, we constructed truncated variants (Fig. 4 A) and transiently transfected them into PIGS-HRD1-CD55-TKO cells. Cells expressing CD55 mutants lacking SCR domains or the S/T region efficiently rescued surface-free GPI expression (Fig. 4 B). However, CD55 lacking a GPI attachment signal (T349–381) could not rescue the expression of free GPIs. A construct only having the 32–amino acid GPI attachment signal (T35–349) also showed no activity, probably due to instability or difficult expression (Fig. 4, B–D). To rescue stable expression, we fused the GPI attachment signal of CD55 with GFP to generate chimera GFP-CD55(C) and transiently overexpressed it in PIGS-HRD1-CD55-TKO cells. GFP-CD55(C) was expressed well and restored generation of surface-free GPIs (Fig. 4, E–G), indicating a unique role of the GPI attachment signal of CD55 in regulating GPI biosynthesis.

**Figure 5.**
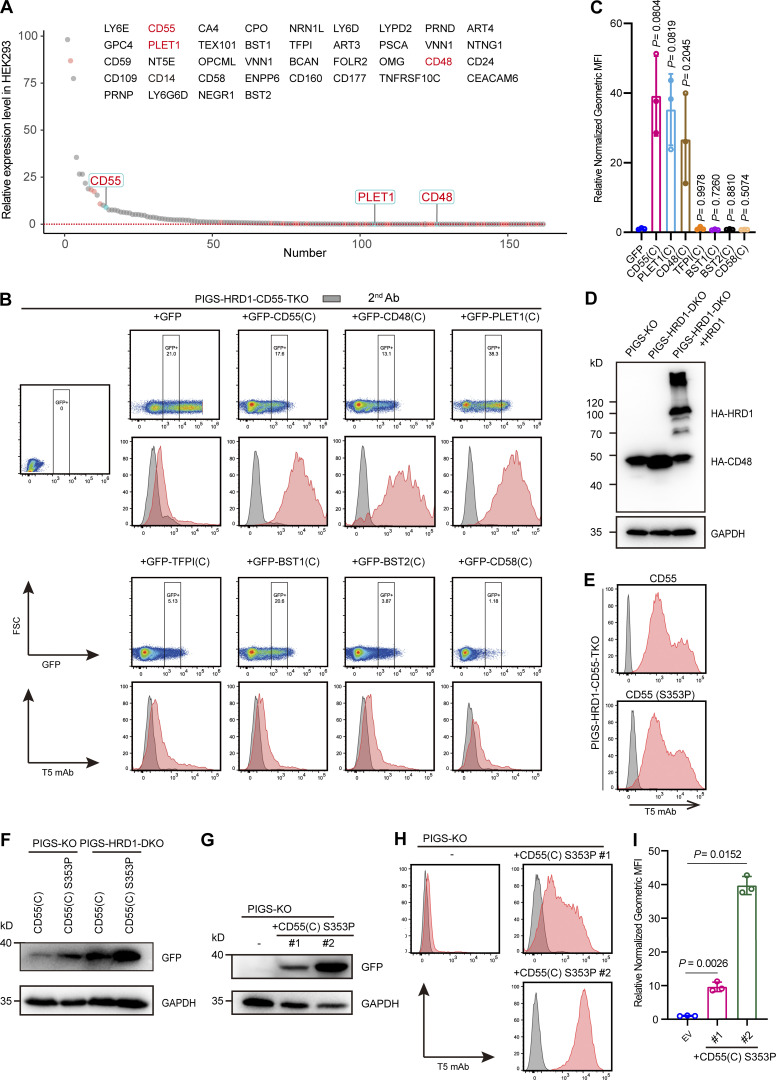
**Specific GPI attachment signal peptides-dependent upregulation of GPI biosynthesis. (A)** Relative levels of mRNAs for GPI-APs in HEK293 cells. Y axis: value of TPM. X axis: number of GPI-APs. Red dots: GPI-APs chosen for KO in PIGS-HRD1-DKO cells or overexpression in PIGS-HRD1-CD55-TKO cells. The tested GPI-APs are listed. CD55, CD48, and PLET1 are highlighted in red. See Table S2 for the entire data. **(B)** Flow cytometry analysis of PIGS-HRD1-CD55-TKO cells transiently expressing GFP-fused GPI attachment signal peptides of various GPI-APs or GFP only. After staining with T5 mAb, cells expressing similar levels of GFP were gated and their T5 mAb staining levels were compared. **(C)** Relative normalized geometric MFI of PIGS-HRD1-CD55-TKO transfected with GFP is compared with those of PIGS-HRD1-CD55-TKO transfected with GFP-fused GPI attachment signal peptides of various GPI-APs and displayed as the mean ± SD from three independent experiments with P values (one-way ANOVA followed by Dunnett’s multiple comparisons test). **(D)** Cell lysates prepared from PIGS-KO, PIGS-HRD1-DKO, and PIGS-HRD1-DKO+HA-HRD1 rescued cells transiently expressing HA-CD48 were analyzed by WB. Protein expression was detected using anti-HA antibody. GAPDH, a loading control. **(E)** PIGS-HRD1-CD55-TKO cells were transiently transfected with cDNAs of EGFP-fused WT CD55(C) or S353P CD55(C). The surface expression of free GPI was stained with T5 mAb and analyzed by flow cytometry. **(F)** Cell lysates prepared from the cells transiently overexpressed EGFP-CD55(C) and S353P CD55(C) in PIGS-KO or PIGS-HRD1-DKO cells were analyzed by WB against anti-GFP antibody. GAPDH, a loading control. **(G–I)** PIGS-KO cells were stably transfected with the GFP-fused CD55(C) S353P mutant, and two single clones, #1 and #2, were isolated. The expression of GFP-fused CD55(C) S353P was analyzed using WB (G). GAPDH, a loading control. The surface expression of free GPI was stained with T5 mAb and analyzed by flow cytometry (H). Relative normalized geometric MFI in PIGS-KO is compared with clones #1 and #2 and displayed as the mean ± SD from three independent experiments with P values (one-way ANOVA followed by Dunnett’s multiple comparisons test; I). Source data are available for this figure: SourceData F5.

**Figure S3.**
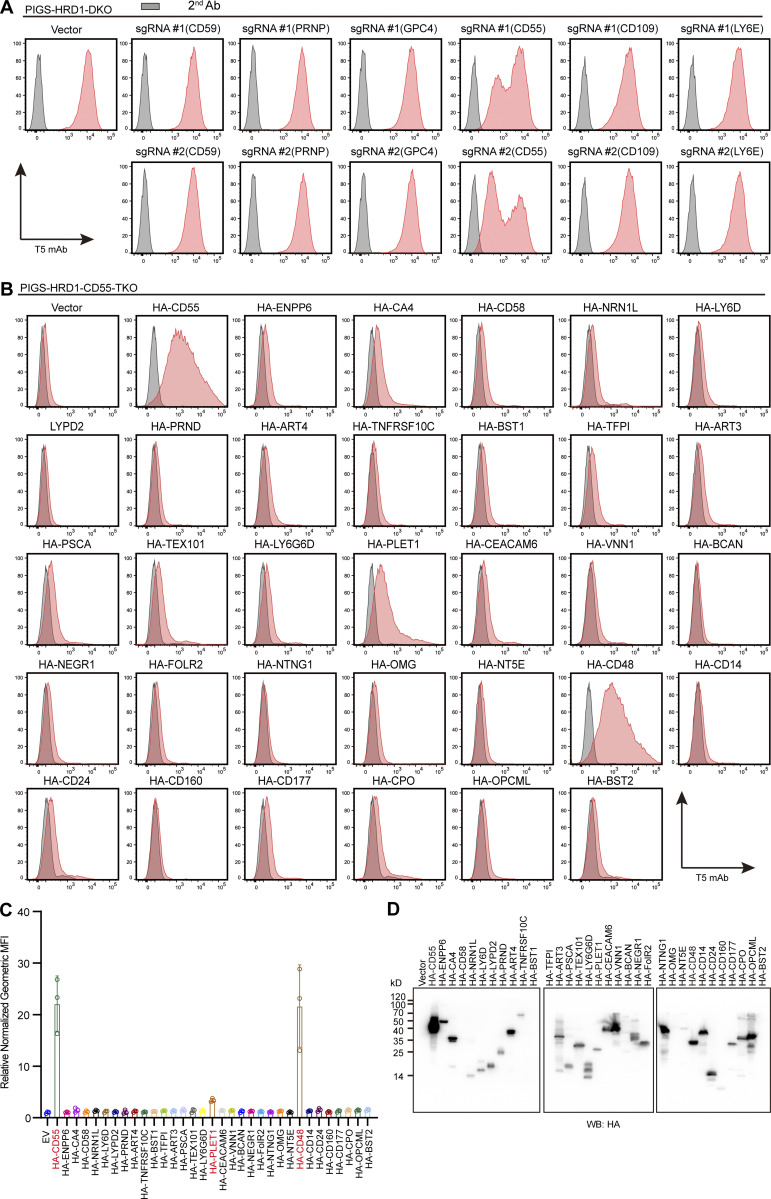
**Exploration of GPI-APs involved in GPI biosynthesis. (A)** Flow cytometry analysis of PIGS-HRD1-DKO cell-transfected plasmids bearing two target gRNAs of CD59, PRNP, CD55, CD109, and LY6E and cultured for 8 d. Cells were stained with T5 mAb. **(B and C)** Flow cytometry analysis of PIGS-HRD1-CD55-TKO cells transiently expressing different HA-tagged GPI-APs. Cells were stained with T5 mAb and quantified. *n* = 3. Normalized geometric MFI in PIGS-HRD1-CD55-TKO cells transfected with EV is compared with PIGS-HRD1-CD55-TKO cells transfected with plasmids of different HA-tagged GPI-APs and displayed as the mean ± SD from three independent experiments (one-way ANOVA followed by Dunnett’s multiple comparisons test). **(D)** Cell lysates prepared from the cells used in B were analyzed by WB. Protein expression was detected using HA antibody. Source data are available for this figure: SourceData FS3.

Considering that more than 150 different human proteins are GPI-APs, we next investigated whether other GPI-AP precursors had the ability to upregulate GPI biosynthesis, similar to CD55. Among GPI-APs expressed in HEK293 cells, 16 were expressed at higher levels than CD55 (Yang et al., 2021; Fig. 5 A). We then chose five of them (LY6E, GPC4, CD59, CD109, and PRNP) and CD55, which were knocked out by two different gRNAs in PIGS-HRD1-DKO cells. All gene KOs except CD55 failed to decrease T5 mAb staining (Fig. S3 A). Conversely, we transfected cDNAs of HA-tagged, 32 GPI-APs that are not expressed in HEK293 cells (RNA-seq data shown in Fig. 5 A and Table S2), and HA-CD55 as a positive control, in PIGS-HRD1-CD55-TKO cells (Fig. S3, B and C). Six (TFP1, BST1, BST2, CD58, OMG, and CD160) were not expressed as assessed by WB against anti-HA antibody (Fig. S3 D). Among 26 expressed HA-tagged GPI-APs, HA-tagged CD48 (HA-CD48) efficiently and HA-tagged placenta-expressed transcript 1 (PLET1) less efficiently restored the expression of free GPIs whereas others were inactive (Fig. S3, B and C).

With the finding that the GPI attachment signal peptide of CD55 was an active element (Fig. 4 E), the GPI attachment signal of CD48 and PLET1 was fused with GFP. For comparison, the GPI attachment signal peptides of TFP1, BST1, BST2, and CD58 were fused with GFP and they were transfected into PIGS-HRD1-CD55-TKO cells. When cells expressing similar levels of GFP were analyzed, GFPs fused with the GPI attachment signal peptides of CD55, CD48, and PLET1 similarly restored the expression of free GPIs whereas those with the GPI attachment signal peptides of TFP1, BST1, BST2, and CD58 did not (Fig. 5, B and C). These results indicate that precursors of some GPI-APs, such as CD55, CD48, and PLET1, particularly their GPI attachment signal peptides, are functional in the positive regulation of GPI biosynthesis.

We next asked whether CD48 precursor is a substrate of ERAD-L pathway like CD55 precursor. HA-CD48 transiently expressed in PIGS-HRD1-DKO cells was stabilized compared with that in PIGS-KO cells (Fig. 5 D). The level of HA-CD48 in PIGS-HRD1-DKO cells was greatly decreased by rescue of HRD1 (Fig. 5 D), indicating that CD48 precursor is also under control of ERAD-L pathway.

According to previous reports that replacement of the ω-site serine 353 in CD55 with proline abolishes GPI attachment (Moran et al., 1991), we constructed a CD55(S353P) mutant in which serine was replaced with proline and transiently overexpressed it in PIGS-HRD1-CD55-TKO cells. CD55(S353P) was active in restoring T5 mAb staining (Fig. 5 E). We next constructed a GFP-fusion of the C-terminal sequence of CD55(S353P), CD55(C)(S353P), and transiently transfected it into PIGS-KO and PIGS-HRD1-DKO cells. Interestingly, the GFP-fusion of CD55(C; S353P) was more stable than the GFP-fusion of WT CD55(C) in PIGS-KO cells, in which the ERAD system functioned normally (Fig. 5 F). This result raised the possibility that stabilization of CD55 in the ER was the only effect of HRD1-KO in the upregulation of GPI biosynthesis. To validate this hypothesis, we stably overexpressed GFP-CD55(C; S353P) in PIGS-KO cells and isolated two clones, #1 and #2, with different levels of GFP-CD55(C; S353P; Fig. 5 G). Even in the presence of HRD1-dependent ERAD-L pathway, the free GPIs stained by T5 mAb were detected in those cells (Fig. 5 H). The T5 mAb staining levels were correlated with the GFP-CD55(C)(S353P) protein levels (Fig. 5, G–I). These results indicated that GPI biosynthesis was increased by the GPI attachment signal peptide of CD55 in a dose-dependent manner.

### Key residues in GPI attachment signal for GPI biosynthesis

**Figure 6.**
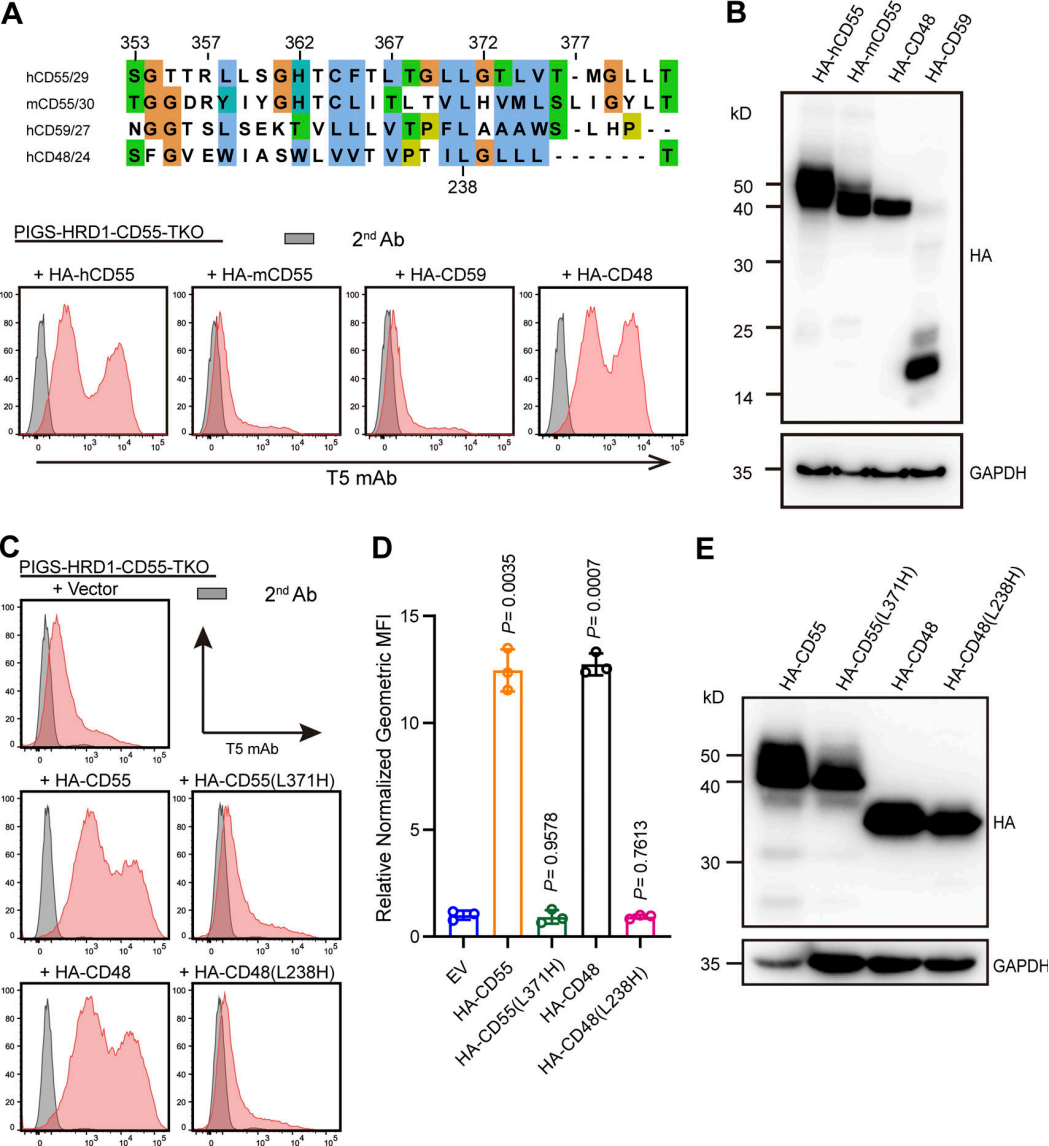
**Exploration of the function-important residues of the GPI attachment signal peptide in GPI biosynthesis. (A)** Upper: GPI attachment signal peptides of human CD55, mouse CD55, CD59, and CD48 are aligned. The conservation of amino acids is shown in colors. Lower: Flow cytometry analysis of PIGS-HRD1-CD55-TKO cells transiently expressing plasmids with HA-tagged human CD55, mouse CD55, CD59, and CD48. Cells were stained with T5 mAb. **(B)** Cell lysates prepared from the cells used in A were analyzed by WB. The expression of fused proteins was detected by HA antibody. GAPDH was used as a loading control. **(C–E)** The indicated mutant CD55 and CD48 constructs were transiently expressed in PIGS-HRD1-CD55-TKO cells. Surface expression of free GPI was detected using flow cytometry. Relative normalized geometric MFI in PIGS-HRD1-CD55-TKO transfected with EV is compared with various transfected cells and displayed as the mean ± SD from three independent experiments with P values (one-way ANOVA followed by Dunnett’s multiple comparisons test). Cell lysates prepared from the cells used in C were analyzed by WB. The expression of fused proteins was detected by HA antibody. GAPDH was used as a loading control. Source data are available for this figure: SourceData F6.

**Figure S4.**
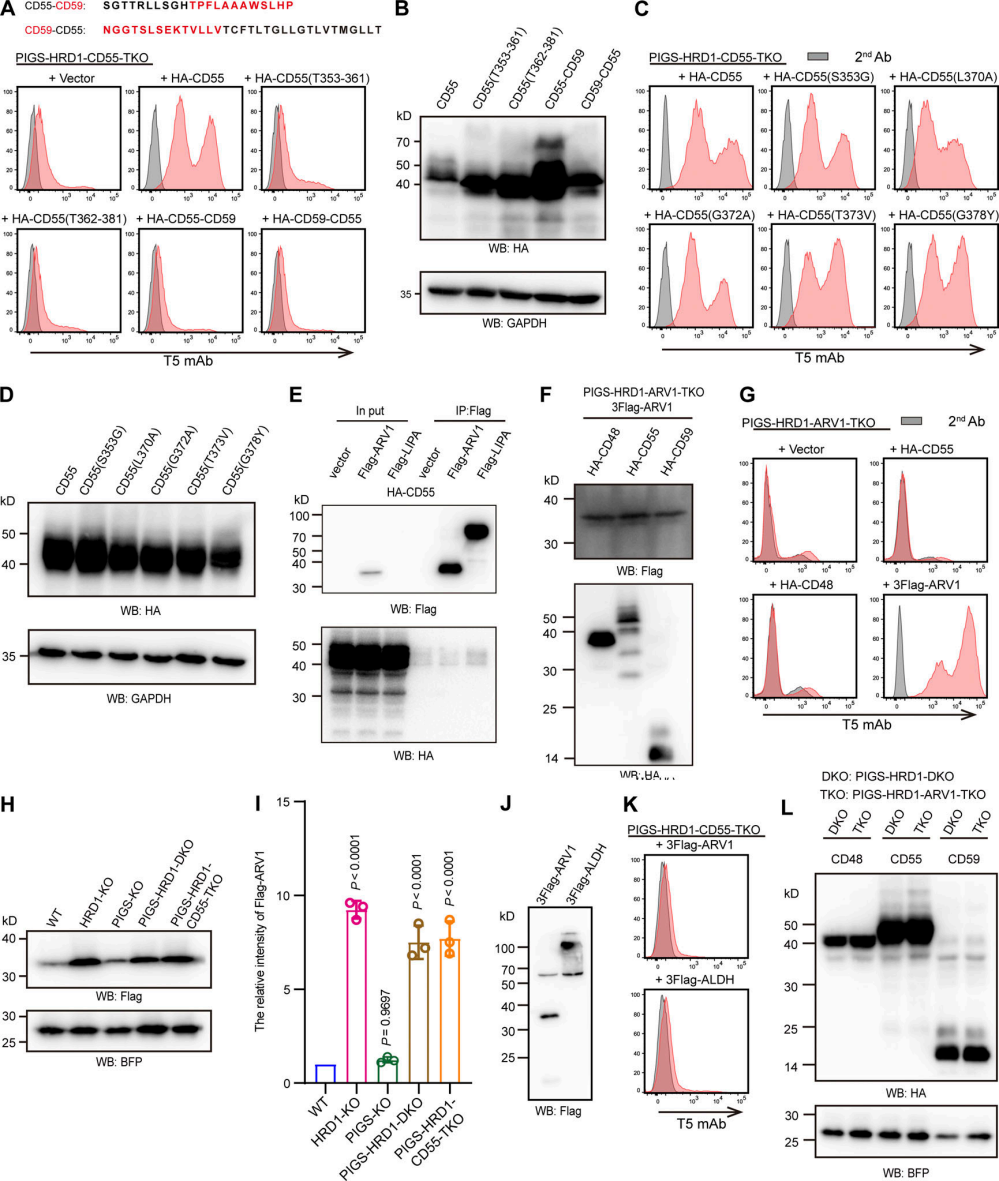
**Function of the GPI attachment signal peptide of CD55 and interaction of ARV1 with CD55. (A)** Upper: Chimera GPI attachment signal peptide of CD55 and CD59. Lower: Flow cytometry analysis of PIGS-HRD1-CD55-TKO cells transfected with the HA-CD55 truncated hydrophilic or the hydrophobic region of GPI attachment signal peptide, and chimera CD55 and CD59. Cells were stained with T5 mAb. **(B)** Cell lysates prepared from the cells used in A were analyzed by WB. Protein expression was detected using HA antibody. GAPDH was used as a loading control. **(C)** Flow cytometry analysis of PIGS-HRD1-CD55-TKO cells transfected with HA-CD55 mutants of GPI attachment signal peptide. Cells were stained with T5 mAb. **(D)** Cell lysates prepared from the cells used in C were analyzed by WB. Expression of mutant proteins was detected using the HA antibody. GAPDH was used as a loading control. **(E)** Cell lysates prepared from PIGS-HRD1-CD55-TKO cells that expressed HA-CD55 stably and Flag-ARV1 or Flag-LIPA transiently were immunoprecipitated with anti-Flag beads. Samples were analyzed by WB. Proteins were detected using the Flag and HA antibodies. **(F)** Cell lysates prepared from PIGS-HRD1-ARV1-TKO cells stably expressing 3Flag-ARV1 transiently expressing HA-tagged CD48, CD55, and CD59 were analyzed by WB. Protein expression was detected using the Flag and HA antibodies. **(G)** Flow cytometry analysis of PIGS-HRD1-ARV1-TKO cells transfected with HA-CD55, HA-CD48, and 3Flag-ARV1. Cells were stained with T5 mAb. **(H)** Cell lysates prepared from WT, HRD1-KO, PIGS-KO, PIGS-HRD1-DKO, and PIGS-HRD1-CD55-TKO cells transiently coexpressed 3Flag-ARV1 and BFP were analyzed by WB. Protein expression was detected using the Flag and BFP antibodies. BFP was used as a loading control. **(I)** The relative intensity of ARV1 level in H were displayed as the value ± SD from three independent experiments (one-way ANOVA followed by Dunnett’s multiple comparisons test). **(J and K)** PIGS-HRD1-CD55-TKO cells transiently expressed 3Flag-ARV1 and 3Flag-ALDH were analyzed by WB using the Flag antibody (J) and flow cytometry using T5 mAb (K). **(L)** Cell lysates prepared from PIGS-HRD1-DKO and PIGS-HRD1-ARV1-TKO cells transiently coexpressed HA-tagged CD48, CD55, or CD59 were analyzed by WB. Protein expression was detected using HA antibody. Cotransfected BFP was used as a loading control. Source data are available for this figure: SourceData FS4.

GPI transamidase recognizes and cleaves the GPI attachment signal between ω and ω+1 sites, forming the thioester bond on the catalytic Cys residue to generate an enzyme–substrate intermediate. GPI is then transferred to the newly exposed C-terminus of the protein (Eisenhaber et al., 2014; Yang et al., 2021). Although all proteins modified by GPI contain a GPI attachment signal peptide at the C-terminus, the sequences are not conserved. We compared the GPI attachment signal peptide sequences of human CD55, mouse CD55, human CD59, and CD48 and tested their activities. Although human CD55 and mouse CD55 sequences showed some identity, mouse CD55 and human CD59 did not have the ability to restore free GPI expression in PIGS-HRD1-CD55-TKO cells (Fig. 6, A and B). Since the C-terminal GPI attachment signal possesses 5–10 hydrophilic amino acids (spacer sequence) and a 15–20 stretch of hydrophobic amino acids, we truncated the hydrophilic and hydrophobic regions of human CD55. Neither truncated construct (T353–361 and T362–381) showed activity (Fig. S4, A and B). We also made chimeras by exchanging hydrophilic and hydrophobic regions of functional CD55 and non-functional CD59, respectively, and overexpressed the constructs in PIGS-HRD1-CD55-TKO cells. Chimeric proteins could not restore T5 mAb staining (Fig. S4, A and B), suggesting that both hydrophilic and hydrophobic regions are important for the activity. We then focused on amino acids among GPI attachment signal sequences and constructed point mutants. Since non-functional CD59 and mouse CD55 had histidine within the hydrophobic region, we introduced histidine into CD55 and CD48 by replacing one of the leucines. The CD55 L371H and CD48 L238H mutants lost activity, even if the mutant proteins were expressed at similar levels (Fig. 6, C–E). Other CD55 mutants (S353G, L370A, G372A, T373V, and G378Y) maintained the functional activity (Fig. S4, C and D). These findings imply that histidine in the hydrophobic region in the signal peptides is inhibitory for their ability to enhance GPI biosynthesis.

### ARV1 is closely located to CD55 and CD48

**Figure 7.**
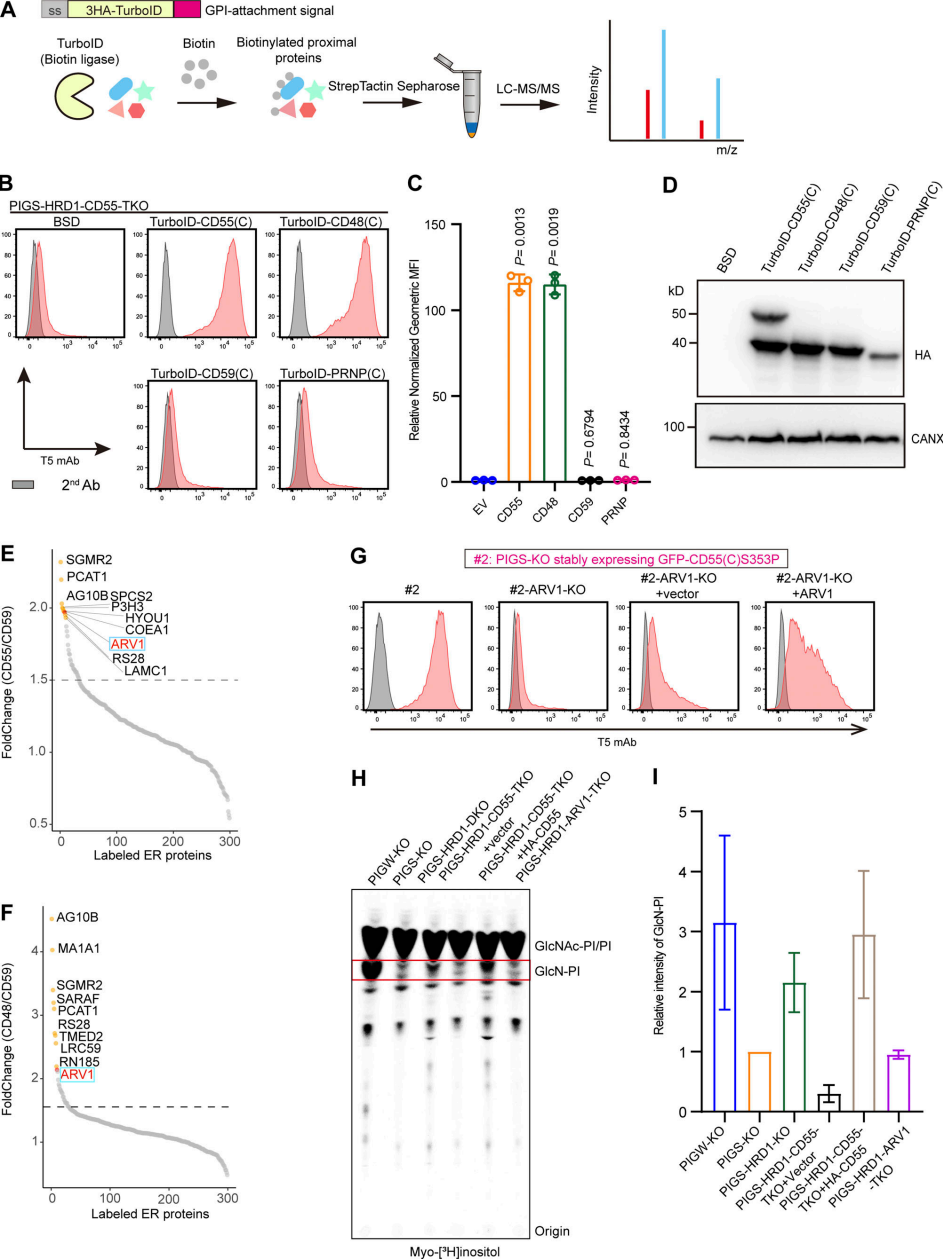
**ARV1 is as spatially close to CD55 and CD48, and functionally required for CD55-dependent upregulation of GPI biosynthesis. (A)** Schematic strategy of biotin labeling and MS-based protein identification. A 3× HA-tagged biotin ligase (Turbo-ID) was fused with the GPI attachment signal peptide. **(B–D)** PIGS-HRD1-CD55-TKO cells were stably transfected with EV and 3× HA-tagged TurboID fused with GPI attachment signal peptides of the CD55, CD48, CD59, and PRNP. The surface expression of free GPI was stained with T5 mAb and analyzed by flow cytometry. Relative normalized geometric MFIs of PIGS-HRD1-CD55-TKO cells transfected either with EV or various TurboID constructs are displayed as the mean ± SD from three independent experiments with P values (one-way ANOVA followed by Dunnett’s multiple comparisons test). Cell lysates prepared from the cells used in B were analyzed by WB. The expression of fused proteins was detected using the HA antibody. CANX was used as a loading control. An extra band of 50 kD in TurboID-CD55(C) transfected cells is of unknown origin. **(E)** The fold change in TurboID-CD55(C) versus TurboID-CD59(C) biotin and TMT-labeled peptide. A fold change ranking at the top 10 is highlighted in orange, and ARV1 is highlighted in red. **(F)** The fold change in TurboID-CD48(C) versus TurboID-CD59(C) biotin and TMT-labeled peptide. A fold change ranking at the top 10 is highlighted in orange, and ARV1 is highlighted in red. See Table S3 for the entire data. **(G)** Flow cytometry of PIGS-KO HEK293 cells stably expressing GFP-CD55(C) S353P (clone #2), ARV1-KO clone #2 cells, and ARV1-KO cells of clone #2 transiently transfected with EV or vector containing 3× Flag-tagged ARV1. Cells were stained with T5 mAb. **(H)** HPTLC analysis of GlcN-PI from cells metabolically labeled with [2-^3^H] inositol for 24 h. **(I)** Relative mean values of GlcN-PI spots in H are displayed as the mean ± error bar from two independent experiments. BSD, blasticidin S deaminase. Source data are available for this figure: SourceData F7.

With the finding that the GPI attachment signal peptides of CD55 and CD48 are involved in upregulation of GPI biosynthesis, we next investigated the underlying mechanism. To identify proteins that might physically interact with them, we used a proximity-tagging system, Turbo-ID. First, we added two functional GPI attachment signal peptides of CD55 and CD48, and two non-functional ones of CD59 and PRNP to the C-terminus of Turbo-ID (Fig. 7 A), which were stably expressed in PIGS-HRD1-CD55-TKO cells (Fig. 7 D). TurboID-CD55(C) and TurboID-CD48(C) restored free GPI expression, as expected, whereas TurboID-CD59(C) and TurboID-PRNP(C) had no activity (Fig. 7, B and C). To identify proteins that specifically interacted with CD55, we compared biotinylated proteins by TurboID-CD55(C) and TurboID-CD48(C) with those by TurboID-CD59(C). ARV1, which was earlier found in the genome-wide screening for positive regulators of GPI biosynthesis (Fig. 2, C–E), was ranked in the top 10 after comparing normalized peptide counts of CD55 with CD59 or CD48 with CD59 (Fig.7, E and F). To test whether CD55 directly interacted with ARV1, we performed immunoprecipitation experiments by transfecting 3Flag-ARV1 into PIGS-HRD1-CD55-TKO cells stably expressing HA-CD55. We did not detect coprecipitation of ARV1 with CD55 under the tested conditions, suggesting that the interaction was very weak or transient, or that levels of one or more other proteins required to form ARV1- and CD55-containing complexes were very low (Fig. S4 E). The 3Flag-ARV1 levels were similar in the presence or absence of HA-CD55 in PIGS-HRD1-ARV1-TKO cells; therefore, ARV1 and CD55 precursors did not stabilize each other (Fig. S4 F).

**Figure S5.**
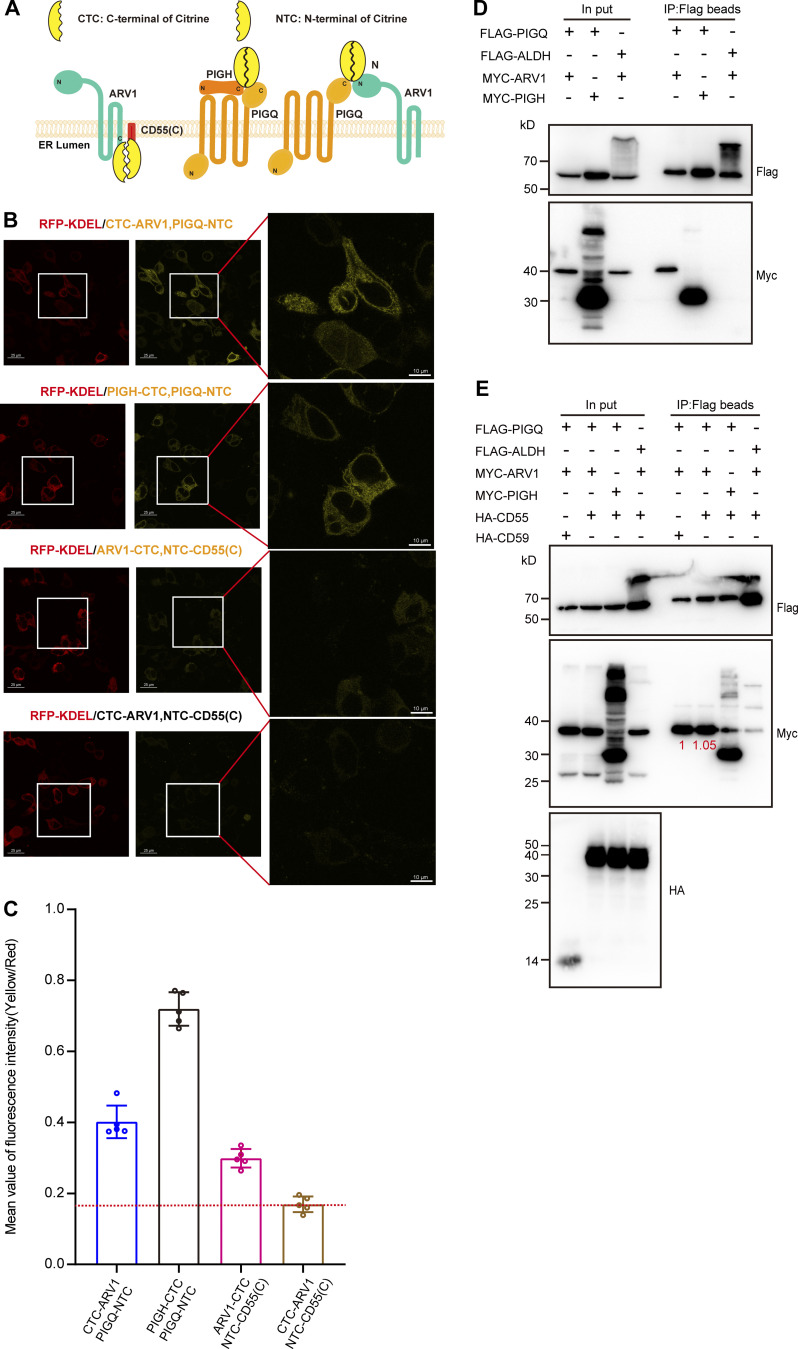
**Analysis of the interaction of ARV1 with CD55 or PIGQ. (A)** Schematic model of the BiFC system. Split Citrines (N-fragment and C-fragment) were fused at C-termini or N-termini of ARV1, PIGQ, CD55(C), and PIGH. When the N-fragment and C-fragment of Citrine interact with each other, mature fluorescence protein is generated. **(B)** Fluorescence images of PIGS-HRD1-CD55-TKO cells transiently coexpressing RFP-KDEL and split Citrine fused proteins. **(C)** The mean value of the fluorescence intensity of cells shown in B was calculated by ImageJ and displayed as the mean (Yellow/Red) ± SD of five independent cells. **(D)** Cell lysates prepared from PIGS-HRD1-CD55-TKO transfectants were immunoprecipitated with anti-Flag beads. Samples were analyzed by WB. Proteins were detected using the Flag or Myc antibodies. **(E)** Cell lysates prepared from PIGS-HRD1-CD55-TKO transfectants were immunoprecipitated with anti-Flag beads. Samples were analyzed by WB. Proteins were detected using the Flag, HA, or Myc antibodies. Coimmunoprecipitated level was indicated with numbers in red. Source data are available for this figure: SourceData FS5.

To further test direct interaction of CD55(C) and ARV1, we used a split-YFP system, bimolecular fluorescence complementation (BiFC) assay (Verhoef and Wade, 2017; Fig. S5 A). The N-terminal subdomain of monomeric Citrine (mCitrine, a mutant YFP) was fused to the N-terminus of CD55(C) [indicated NTC-CD55(C)], and the C-terminal subdomain of mCitrine was fused either to the N- or the C-terminus of ARV1 [indicated CTC-ARV1 or ARV1-CTC, respectively], and they and RFP-KDEL (an ER marker) were co-transfected into PIGS-HRD1-CD55-TKO cells. A weak fluorescence was induced in the ER when NTC-CD55(C) was co-transfected with ARV1-CTC but not CTC-ARV1 (Fig. S5, B and C). This result indicates that ARV1 and CD55(C) directly but weakly interact with each other in the ER. The result also indicates that ARV1 has its N-terminus in the cytoplasmic side and its C-terminus in the luminal side of the ER.

### ARV1 is required for upregulation of GPI biosynthesis by CD55

Since both CD55 and ARV1 were required for the upregulation of GPI biosynthesis in PIGS-HRD1-KO cells, we next asked whether ARV1 was required for the upregulation of GPI biosynthesis by CD55 and CD48. The increased T5 mAb staining level induced by the stably expressed GFP-CD55(C)(S353P) in PIGS-KO cells (clone #2) was decreased to the background level by knocking out ARV1 and restored by transfection of ARV1 (Fig. 7 G). Overexpression of HA-CD55 or HA-CD48 in PIGS-HRD1-ARV1-TKO cells could not restore the T5 mAb staining (Fig. S4 G); therefore, ARV1 was functionally required for CD55 and CD48 precursors. ARV1 levels were eight times higher in HRD1-KO and PIGS-HRD1-DKO cells than in WT and PIGS-KO cells (Fig. S4, H and I), suggesting that ARV1 increases and plays a critical role under stressed conditions. However, overexpression of ARV1 alone in PIGS-HRD1-CD55-TKO HEK293 cells did not induce positive T5 mAb staining (Fig. S4, J and K). The levels of CD55 and CD48 precursors were not changed in the absence or presence of ARV1(Fig. S4 L). These results suggested that both CD55/CD48 and ARV1 are prerequisites for the upregulation of GPI biosynthesis.

Finally, we investigated steps in the GPI biosynthesis pathway being upregulated by ARV1 and the CD55 precursor. ARV1 was implicated in the initial step in GPI biosynthesis because trypanosome ARV1 homolog was physically associated with the first enzyme GPI-GlcNAc transferase and because yeast ARV1 was predicted to be associated with GPI1, a component of GPI-GlcNAc transferase (Ikeda et al., 2016; Ji et al., 2021; Okai et al., 2020). We first tested association of ARV1 with PIGQ, human homolog of GPI1, and demonstrated coimmunoprecipitation of ARV1 and PIGQ (Fig. S5 D). The coimmunoprecipitation levels were similar in the absence and presence of overexpressed CD55 (Fig. S5 E), suggesting that CD55 precursor is not involved in the association. Association between ARV1 and PIGQ was also demonstrated by the BiFC assay using CTC-ARV1 and PIGQ-NTC (Fig. S5, A–C). These results support the idea that ARV1 is a regulator of GPI biosynthesis acting in the first step.

The [2-^3^H] mannose labeling showed that all spots of GPI intermediates containing mannoses were increased in PIGS-HRD1-DKO cells and their CD55-overexpressing cells (Fig. 3 C), indicating that biosynthesis was upregulated by the CD55 precursor at an earlier step before mannose addition. To determine which step was regulated by CD55, myo-[2-^3^H]-inositol labeling was performed in PIGW-KO (Murakami et al., 2003), PIGS-KO, PIGS-HRD1-DKO, PIGS-HRD1-CD55-TKO, and PIGS-HRD1-ARV1-TKO cells (Fig. 7, H and I). Radioactive inositol was incorporated into PI and then used to generate the first, second, and third GPI intermediates GlcNAc-PI, GlcN-PI, and GlcN-acylPI. After thin-layer chromatography and detection by phosphor imaging, GlcN-PI, which accumulates in PIGW-KO cells, appeared below the abundant PI spots, whereas GlcNAc-PI and GlcN-acylPI did not appear as discrete bands due to overlapping migration with abundant PI and other inositol-containing components (Murakami et al., 2003). GlcN-PI was increased in PIGS-HRD1-DKO cells compared with PIGS-KO cells, while knocking out CD55 eliminated GlcN-PI increase, which was rescued by CD55 transfection (Fig. 7, H and I). Knocking out ARV1 similarly prevented the GlcN-PI increase (Fig. 7, H and I), suggesting that both CD55 and ARV1 were required for the upregulation of GPI biosynthesis in the early step.

## Discussion

**Figure 8.**
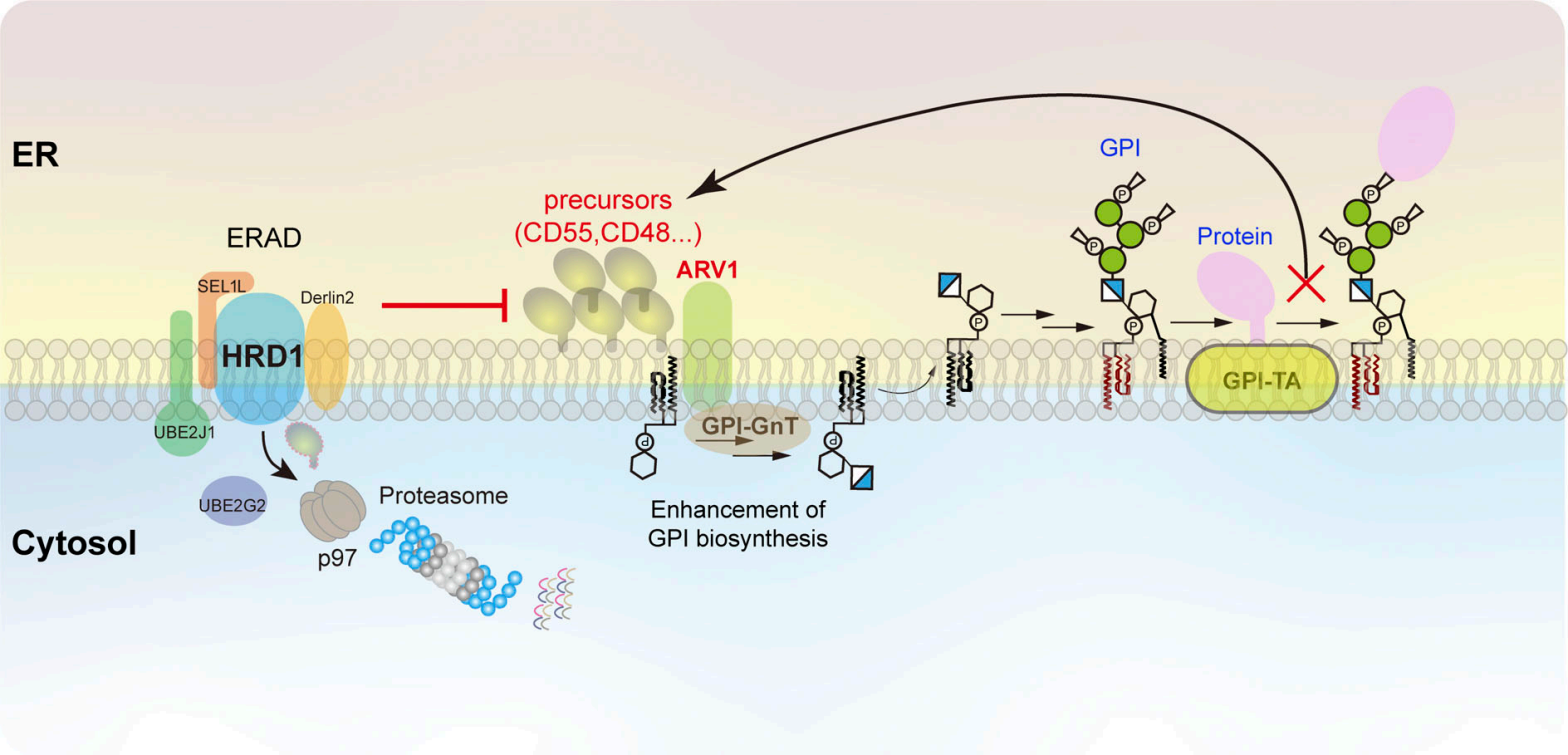
**Schematic model of the functional roles of the HRD1-dependent ERAD-L substrate CD55 precursor in regulating GPI biosynthesis.** CD55 precursor proteins and other GPI-AP precursor proteins are processed and transferred to GPI by the GPI transamidase (GPI-TA), generating GPI-anchored forms. Conversely, these precursor proteins that failed to have GPI attachment are degraded through the HRD1-dependent ERAD-L pathway. Once the ERAD-L pathway is impaired, CD55 precursor proteins accumulate in the ER, in turn upregulating GPI biosynthesis at early step(s). ARV1 associating with GPI-GnT is critical for the upregulation of GPI biosynthesis under CD55 accumulated conditions.

Although the majority of genes required for GPI precursor biosynthesis, GPI transfer to proteins, and GPI remodeling have been identified, understanding the dynamic regulation of GPI biosynthesis is still a challenge. This is in part due to technical obstacles in directly detecting changes in GPI levels. Here, we designed and implemented a free GPI expression system under GPI transamidase- and ERAD-deficient conditions. Detection of free GPI expression provides a new approach for rapidly determining the status of GPI biosynthesis and can be used to understand the regulatory mechanism. In this work, we used the enhancement of free GPI expression in PIGS-HRD1-DKO cells to identify genes that regulate GPI biosynthesis. The screening facilitated the discovery of CD55 precursor, which is the substrate of the ERAD-L pathway and upregulates GPI biosynthesis upon accumulation (Fig. 8).

Under normal conditions, a GPI attachment signal of precursor protein is recognized and cleaved by GPI transamidase during GPI modification (Liu et al., 2021). Most likely, the ratio of GPI and proteins is controlled properly. It is difficult to detect either the accumulation of GPI or precursor proteins in WT cells. Normally, misfolded GPI-anchored PrP* (A117V) and Gas1* (G291R) are poor ERAD substrates because of the specific characteristics of GPI anchors (Ashok and Hegde, 2008; Satpute-Krishnan et al., 2014; Sikorska et al., 2016). In stressed mammalian cells, misfolded GPI-anchored PrP* is transported out of the ER quickly via a pathway termed “rapid ER stress-induced export” for degradation in the lysosome (Satpute-Krishnan et al., 2014). However, misfolded transmembrane type Gas1* (Gas1*-TMD) and PrP maintaining its GPI attachment signal peptide that fails to undergo transamidation are mainly degraded by ERAD (Ashok and Hegde, 2008; Sikorska et al., 2016). In this study, we found that knocking out the PIGS and HRD1 genes caused the accumulation of CD55 precursor proteins, which enabled us to reveal the specific role of the GPI attachment signal peptide of CD55 in GPI biosynthesis. It is reasonable to postulate that CD55 precursor acts as a regulator because it is widely expressed in diverse organs and cells (Karlsson et al., 2021). Moreover, we found that the GPI attachment signal peptides of CD48 and PLET1 functioned similarly to that of CD55. The expression of CD48 and PLET1 is restricted to certain cell types, suggesting the existence of other GPI-AP precursors regulating GPI biosynthesis in a tissue-specific manner. Conversely, mouse CD55 could not restore the human CD55 KO phenotype. There are two types of CD55 in mouse cells, the GPI-anchored form and the transmembrane form, which differs from human CD55. We hypothesize that different and diverse GPI-APs are utilized as regulators of GPI biosynthesis among species.

Several papers have reported that Arv1 plays roles in lipid and cholesterol homeostasis, sphingolipid distribution, and GPI biosynthesis in the yeast *S. cerevisiae* (Georgiev et al., 2013; Swain et al., 2002). It was proposed that in GPI biosynthesis, Arv1 may deliver early GPI intermediate GlcN-acyPI to the ER lumen as the flippase or contribute to the flip reaction, so the loss of Arv1 leads to deficiencies in GPI-AP biosynthesis in *S. cerevisiae* (Kajiwara et al., 2008; Okai et al., 2020). Furthermore, mutations in human ARV1 cause symptoms similar to inherited GPI deficiencies, indicating that human ARV1 is also involved in GPI biosynthesis (Ikeda et al., 2016; Salian et al., 2021). A recent study in the African trypanosome *Trypanosoma brucei* showed that an Arv1-like protein (TbArv1) is pulled down by TbGPI3, the mammalian homolog of which is PIGA (Ji et al., 2021). Using a combination of RoseTTAFold and AlphaFold, a study to predict protein assemblies with two to five components in *S. cerevisiae* also predicted that ARV1 may interact with Gpi1, the yeast homolog of mammalian PIGQ (Humphreys et al., 2021). Indeed, we demonstrated association of human PIGQ and ARV1 by the BiFC assay and coimmunoprecipitation (Fig. S5, B–E), suggesting that ARV1 forms a complex with GPI-GlcNAc transferase (GPI-GnT), the first enzyme in the pathway (Fig. S1 A). Both our CRISPR–Cas9 genetic screen using PIGS-HRD1-DKO cells and proximity labeling using TurboID-CD55(C) and TurboID-CD48(C) identified ARV1, suggesting that ARV1 is spatially close to CD55 and CD48 precursors and is functionally required for CD55-dependent GPI upregulation. We further showed that GPI biosynthesis in PIGS-HRD1-DKO cells is upregulated in early steps, but further KO of CD55 or ARV1 eliminated this effect. CD55 and ARV1 might act in the same pathway, where CD55 serves as the signal of GPI shortage and ARV1 might modulate the proper lipid environments to enhance the activity of GPI-GnT. It will be interesting to further characterize the relationship between the CD55 precursor and ARV1 in the future.

The expression of free GPIs is sensitive to ARV1 KO, indicating that ARV1 is critical for the upregulation of GPI biosynthesis, particularly under conditions where GPI-AP precursor proteins, including CD55 precursor, accumulate. However, in HEK293 WT cells, KO of ARV1 did not change the expression of GPI-APs, suggesting that ARV1 plays a role only in GPI biosynthesis under stressed conditions in cultured cell lines. Indeed, ARV1 levels increased eight times in HRD1-KO HEK293 cells (Fig. S4, H and I). In contrast, primary cells such as blood neutrophils and fibroblasts from patients with ARV1 deficiency had reduced cell-surface levels of GPI-APs (Davids et al., 2020; Salian et al., 2021). Therefore, ARV1 is critical for maintaining normal GPI-APs levels in human body. On the other hand, free GPIs are also present normally as membrane components in several mouse tissues including pons, medulla oblongata, spinal cord, and epididymis (Wang et al., 2019), and human blood cells (Duval et al., 2021). In these tissues/cells, a fraction of synthesized GPI must be kept as free GPIs avoiding transfer to proteins by GPI transamidase. The biosynthesis of GPI-APs is thought to be controlled by the balance of three factors, amounts of GPI and precursor proteins, and GPI transamidase activity. These tissues may be out of balance among the three. GPI biosynthesis is upregulated when ERAD is impaired (Wang et al., 2020). Therefore, once ER stress occurs, free GPIs would be generated in the cells/tissues.

Since the expression of known PIG genes was not changed in the presence or absence of CD55, and overexpression of PIG genes did not restore free GPI expression in PIGS-HRD1-CD55-TKO cells, the regulation was neither at the transcriptional nor the translational level of GPI biosynthetic genes. Thus, the upregulation of GPI biosynthesis by CD55 precursors is a rather quick and short-lived quantity control system for GPI-APs once the downstream GPI transamidation step fails in WT cells. It is possible that CD55 and ARV1 are involved in this regulatory system to balance the biosynthesis of free GPIs and the generation of GPI-APs in the ER.

## Materials and methods

### Cell culture, transfection, and stable cell line generation

HEK293 (CRL-1573; ATCC) cells and all derivative cells were cultured in DMEM with high glucose, glutamine (01-052-1ACS; Biological Industries), and 10% FBS (04-001-1ACS, Biological Industries) at 37°C in a humidified 5% CO_2_ atmosphere. PIGK-KO, GPAA1-KO, PIGT-KO, PIGS-KO, PIGU-KO, PIGS-HRD1-DKO, PIGS-UBE2G2-DKO, PIGS-UBE2J1-DKO, PIGS-GP78-DKO, and SLC35A2-PIGT-DKO cells were previously constructed (Wang et al., 2020). The PIGK-HRD1-DKO, PIGT-HRD1-DKO, PIGU-HRD1-DKO, GPAA1-HRD1-DKO, SLC35A2-PIGT-HRD1-TKO, PIGS-HRD1-SRD5A3-TKO, PIGS-HRD1-ARV1-TKO, PIGS-HRD1-CD55-TKO, PIGS-HRD1-SPPL3-TKO, and PIGS-HRD1-CLPTM1L-TKO cells used in this study were established by the CRISPR–Cas9 system with two different gRNAs, as listed in Table S1. For stable expression, PLAT-GP packaging cells were seeded and cultured to 90% confluence and transfected with the pLIB2-BSD plasmid bearing the cDNA of interest using PEI-Max. Viral medium was added to the cells, and these cells were cultured at 32°C for 12 h. The medium was changed after 24 h and cultured at 37°C. 3 d after infection, the cells were incubated in the medium with 10 μg ml^−1^ blasticidin (InvivoGen) for 14 d.

### Antibodies and reagents

The mouse monoclonal anti–*T. gondii* free GPI antibody, clone T5_4E10 (T5 mAb; 1:100 for FACS), was a generous gift from Dr. Jean François Dubremetz (Montpellier University, France; Striepen et al., 1997). T5 mAb (50267) is now available from BEI Resources, National Institute of Allergy and Infectious Diseases, National Institutes of Health. Mouse monoclonal anti-CD55 (clone IA10; 1:100 for FACS; 1:500 for WB; Kinoshita et al., 1985), anti-CD59 (clone 5H8; 1:100 for FACS; Ashida et al., 2005), anti-Flag (F3165; Sigma-Aldrich; 1:4000 for WB), anti-CANX (M178-3; MBL; 1:4,000 for WB), anti-CD147 (sc-71038; Santa Cruz Biotechnology; 1:1,000 for WB), anti-GAPDH (60004-1-Ig; Proteintech; 1:4,000 for WB), anti-c-Myc (HT101; TransGen Biotech; 1:2,000 for WB), anti-GM130 (610822; BD Biosciences; 1:250 for IF), rabbit monoclonal anti-HA (3724; Cell Signaling Technology; 1:4,000 for WB), and polyclonal anti-GFP (50430-2-AP; Proteintech; 1:4,000 for WB), anti-tRFP (AB233; Evrogen; 1:4,000 for WB) were used as primary antibodies. F(ab′)_2_-goat anti-mouse IgG (H+L) PE (12-4010-82; Thermo Fisher Scientific; 1:200 for FACS), Alexa Fluor 647–conjugated goat against mouse IgM (ab150123; Abcam; 1:400 for FACS), goat anti-mouse IgG (H+L) HRP (HS201; TransGen Biotech; 1:5,000 for WB), goat anti-rabbit IgG (H+L) HRP (HS101; TransGen Biotech; 1:5,000 for WB), Alexa Fluor 488–conjugated goat anti-mouse IgG (A-11008; Thermo Fisher Scientific; 1:500 for IF), and Alexa Fluor 555–conjugated goat anti-rabbit IgG (A-21424; Thermo Fisher Scientific; 1:500 for IF) were used as secondary antibodies. PNGase F (P0704; New England Biolabs) and Endo Hf (P0703; New England Biolabs) were used for cleavage of N-glycans. Biotin (V900418; Sigma-Aldrich) was used for proximity labeling.

### CRISPR–Cas9 screening and FACS

For a large-pooled screen, viral production and functional titration were conducted in the same manner as described previously (Wang et al., 2020). Pooled human GeCKOv2 plasmids (lentiCRISPRv2) were cotransfected with the lentiviral packaging plasmids pLP1, pLP2, and pLP/VSVG (Thermo Fisher Scientific) into Lenti-X 293T cells (Clontech). 12 h later, the medium was changed to 10 ml prewarmed DMEM supplemented with 10% FBS. The viral media was collected 24, 48, and 72 h after transfection and filtered through a membrane (Millex 0.45 µm, poly vinylidene di-fluoride, 33 mm). Finally, 30 ml viral media in total was combined and stored at 4°C for functional titration and pooled screening as quickly as possible.

PIGS-HRD1-DKO cells were plated in 8 × 15 cm dishes (3.5 × 10^6^ cells per dish). Approximately, 8 × 10^7^ cells (1 × 10^7^ cells per dish) were transduced with viral supernatant after 36 h of seeding. Cells were selected with 0.5 μg ml^−1^ puromycin until the infected cells were expanded to 2.4 × 10^8^ to maintain the complexity of the gRNA library. Cells were combined and split 1:4, and a minimum of 6 × 10^7^ cells were plated for culture. At 2 wk after transduction, a pellet of 5 × 10^7^ cells without sorting was stored at −80°C. For cell sorting, ∼1 × 10^8^ cells were harvested and incubated with T5 mAb, followed by staining with anti-mouse IgM. After washing with PBS, the cells were resuspended in Hanks’ Balanced Salt Solution (H6648; Sigma-Aldrich), and T5 mAb staining-negative cells were sorted by FACSAria (BD). We prepared 2 × 10^7^ cells for the second sorting. After sorting, the cells were maintained in DMEM supplemented with 0.25 μg ml^−1^ puromycin. Pellets of 2 × 10^7^ sort2 cells were stored at −80°C until use. For analysis, cells were cultured in 6-well plates 1 d before analysis. The cells were harvested and washed once with PBS and then stained with CD59 or T5 mAb in FACS solution (PBS containing 1% BSA and 0.1% NaN_3_) on ice for 25 min. They were then washed twice in FACS buffer, followed by staining with Alexa Fluor 647–conjugated goat anti-mouse IgM or PE-conjugated goat anti-mouse IgG. After two washes with FACS buffer, the cells were analyzed using a BD FACSCanto II.

### Genomic DNA sequencing and analysis

Approximately, 5 × 10^7^ unsorted and 2 × 10^7^ twice-sorted PIGS-HRD1-DKO cells were extracted for genomic DNA using a Wizard Genomic DNA Purification Kit (Promega). The gRNAs were amplified from genomic DNA of unsorted and twice-sorted cells. PCR (25 cycles) was performed to amplify the gRNAs using KOD FX Neo Polymerase (TOYOBO LIFE SCIENCE), making up a total of 65 tubes for unsorted cells and 12 tubes for twice-sorted cells (oligos for amplification of gRNAs are shown in Data S1). PCR products for unsorted cells (1,050 μl) and for twice-sorted cells (250 μl) were applied to a 2% agarose gel for purification. The PCR products were concentrated, mixed (unsorted to sorted 4.2:1), and analyzed by paired-end sequencing with a NovaSeq 6000 system (Illumina). Deep sequencing raw data were processed for gRNA counting using Python scripts. The high-throughput sequencing reads were demultiplexed using the 5-bp adapter by cutadapt version v1.18 (Martin, 2011). By using MAGeCK workflow version 0.5.8, the adapters of the demultiplexed reads were trimmed to obtain 20-bp gRNA sequences, and the single-guide RNA (sgRNA) sequences were mapped to the sequences of the Human GeCKO v2 sgRNA library to determine the total number of gRNA counts. The robust rank aggregation values and P values were determined using the MAGeCK algorithm (Li et al., 2014).

### Plasmids

All primers used in this study are listed in Table S1. To knock out target genes with the CRISPR–Cas9 system, sgRNAs were designed with the E-CRISP website (http://www.e-crisp.org/E-CRISP/) and the targeting DNA fragments were ligated into the BbsI-digested vector pX330-EGFP. To construct pME-HRD1-3HA and pME-SPPL3-3HA, HRD1 and SPPL3 were amplified from a human cDNA library, digested with XhoI and MulI, and cloned into the same site of pME-B3GALT4-3HA. The HRD1-3HA fragment from pME-HRD1-3HA was digested and ligated to pLIB2-BSD through EcoRI and NotI. SRD5A3 and CLPTM1L fragments were digested with EcoRI and NotI, and pLIB2-BSD-SRD5A3 and pLIB2-BSD-CLPTM1L were constructed. The fragments of ARV1 and PIGQ were digested with SalI and NotI and ligated into pME-3Flag and pME-N6myc to construct pME-3Flag-ARV1, pME-3Flag-PIGQ, and pME-N6myc-ARV1. PIGH was amplified, digested with SalI and XbaI, and ligated into pME-N6myc to construct pME-N6myc-PIGH. Human CD55, CD48, CD59, PRNP, and mouse CD55 (mCD55) were amplified, digested, and ligated into pME-puro-ssHA-GPI, which contains a CD59 signal sequence, one HA tag, and a GPI attachment signal sequence of CD55, through XhoI and NotI to generate pME-puro-ssHA-CD55, pME-puro-ssHA-CD48, pME-puro-ssHA-CD59, pME-puro-ssHA-PRNP, and pME-puro-ssHA-mCD55. GPI attachment signals of CD55, CD48, PLET1, TFPI, BST1, BST2, and CD58 were amplified from pME-puro-ssHA-GPI-APs (CD55, CD48, PLET1, TFPI, BST1, BST2, and CD58) and cloned into pME-ssGFP to generate pME-ssGFP-CD55(C), pME-ssGFP-CD48(C), pME-ssGFP-PLET1(C), pME-ssGFP-TFPI(C), pME-ssGFP-BST1(C), pME-ssGFP-BST2(C), and pME-ssGFP-CD58(C). The ssGFP-CD55(C) fragment was cut with EcoRI and NotI and ligated into pLIB2-BSD to generate pLIB2-BSD-ssGFP-CD55(C). pME-ssHA-CD55 was used as a template and mutagenized with different primers to construct truncated CD55. Plasmids harboring mutant CD55, CD48, or CD55(C) were constructed by site-direct mutagenesis based on pME-puro-ssHA-CD55, pME-puro-ssHA-CD48, pME-puro-ssHA-mCD55, and pLIB2-BSD-ssGFP-CD55(C). 3HA-TurboID was amplified from 3xHA-TurboID-NLS_pCDNA (3107171; Addgene) and cloned into pME-ssCD59-EGFP to generate pME-ssCD59-3HA-TurboID. The GPI attachment signals of CD55, CD48, CD59, and PRNP were amplified and ligated into pME-ssCD59-3HA-TurboID-CD55(C), pME-ssCD59-3HA-TurboID-CD48(C), pME-ssCD59-3HA-TurboID-CD59(C), and pME-ssCD59-3HA-TurboID-PRNP(C), followed by digestion with EcoRI and NotI and cloned into pLIB2-BSD. To detect the protein–protein interaction by BiFC assay, PIGQ and PIGH were infused into pME-puro-NTC, which contains N-fragment of Citrine, and pME-puro-CTC, which contains C-fragment of mCitrine, to generate pME-puro-PIGQ-NTC and pME-puro-PIGH-CTC. To construct pME-puro-CTC-ARV1 and pME-puro-NTC-CD55(C), the fragments of ARV1 and CD55(C) were amplified and digested with XhoI and NotI and then ligated into pME-puro-CTC and pME-puro-NTC. For pME-puro-ARV1-CTC, the fragment of ARV1 was amplified and digested with SalI and XhoI and then ligated into pME-puro-CTC.

### Generation of KO cell lines

To knock out SRD5A3, ARV1, CD55, SPPL3, and CLPTM1L, cells were transfected with one or two different pX330-EGFPs containing the target sgRNA. Lipofectamine 2000 (11668019; Thermo Fisher Scientific) was used for transfection. 3 d after transfection, the GFP-positive cells were sorted with a cell sorter S3e (Bio-Rad). The sorted cells were cultured for 1 wk or more and then diluted to select clonal KO cells. Clonal cells were determined by rescue experiments and WB analysis. KO of HRD1 in PIGK-KO, GPAA1-KO, PIGT-KO, and PIGU-KO or CD59, PRNP, GPC4, CD109, and LY6E in PIGS-HRD1-DKO cells was carried out within the bulk cells. After transient transfection of sgRNA constructs as described above, the bulk cells were sorted and cultured for 10 d, followed by flow cytometry analysis.

### SDS-PAGE and WB

WB was performed to confirm the expression of recombinant proteins. Cells (∼10^6^ cells per well) were lysed with 60 μl of radioimmunoprecipitation assay (RIPA) lysis buffer (50 mM Tris, pH 8, 150 mM NaCl, 0.1% SDS, 0.5% sodium deoxycholate, 1% Triton X-100, 1 mM phenylmethylsulfonyl fluoride [HY-B0496; MedChemExpress], and protease inhibitor cocktail [HY-K0010; MedChemExpress]) on ice for 30 min. After incubation, the sample was centrifuged at 21,600 × *g* for 10 min at 4°C to remove the insoluble fraction. The supernatant was mixed with SDS-PAGE loading buffer and boiled at 95°C for 5 min or kept at 4°C overnight (for multitransmembrane proteins). The proteins were separated by SDS-PAGE and transferred onto poly vinylidene di-fluoride membranes, and the membranes were blocked at RT in Tris-buffered saline containing 0.1% Tween-20 (TBS-T) and 5% nonfat milk for 1 h, followed by incubation with primary antibodies at RT for 1 h. After washing three times with TBS-T buffer, the membranes were then incubated with HRP-labeled secondary antibodies for 1 h at RT. After washing with TBS-T, the membrane was visualized with ECL Prime WB Detection Reagent (GE Health care).

### Fluorescence imaging

To detect the subcellular localization of HA-tagged CD55, PIGS-HRD1-CD55-TKO cells stably expressing HA-CD55 were seeded on glass coverslips pretreated with 1% gelatin and cultured for another 2 d. Cells were washed with PBS, fixed in 4% paraformaldehyde, washed with PBS, and incubated with 40 mM ammonium chloride. Then, the cells were incubated at RT in blocking buffer A (PBS containing 5% FBS) for 1 h. Mouse anti-CANX (M178–3; MBL) and rabbit monoclonal anti-HA (3724; Cell Signaling Technology) were used as the primary antibodies diluted in blocking buffer for 1 h. Cells were gently washed with PBS twice. Alexa Fluor 555–conjugated goat anti-rabbit IgG (A-21424; Thermo Fisher Scientific) and Alexa Fluor 488–conjugated goat anti-mouse IgG (A-11008; Thermo Fisher Scientific) were used as the secondary antibodies and diluted in blocking buffer for 1 h. The cells were gently washed with PBS twice. Finally, the coverslips were mounted onto slides using a mounting solution containing DAPI for 5 min and visualized by a confocal microscope (C2si; Nikon) with a CFI Plan Apochromat VC oil objective lens (100× magnification and 1.4 NA) at RT. Images were collected from three to six random fields in each slide. The experiments were performed at least three times. Image acquisition was controlled and processed by NIS-Elements AR 4.30.00 Software.

To detect the interaction of ARV1 with PIGQ or CD55, split mCitrines (N-fragment and C-fragment) were fused at the C-termini or N-termini of ARV1, PIGQ, CD55(C), and PIGH. Combinations of CTC-ARV1 and PIGQ-NTC, ARV1-CTC and NTC-CD55(C), PIGH-CTC and PIGQ-NTC, or ARV1-CTC and NTC-CD55(C) were coexpressed with plasmid containing RFP-KDEL (an ER marker) in PIGS-HRD1-CD55-TKO cells. Followed by culturing in glass dishes for 2 d, cells were washed with PBS, fixed in 4% paraformaldehyde, washed with PBS, and incubated with 40 mM ammonium chloride. Finally, the cells on the glass dishes were observed by a 63 × 1.4-NA oil-immersion objective on a confocal microscope (Leica TCS SP8) at RT. Leica LASX software was used for image controlled as well as for postacquisition image analysis. The intensity of fluorescence of images and show of indicated regions were acquired by Image J. The experiments were performed at least three times.

### RNA-seq

RNA-seq and analysis were conducted as previously described (Huang et al., 2021). Total RNA was extracted from whole cells with the mirVana miRNA Isolation Kit (Thermo Fisher Scientific) according to the manufacturer’s protocol. RNA quality and integrity were evaluated with an Agilent 2100 Bioanalyzer (Agilent Technologies). Samples with an RNA integrity number ≥7 were considered to be of high quality and were processed further and subjected to subsequent analysis. Total RNA-seq libraries were generated using 4 μg of total RNA, which was analyzed using the TruSeq Stranded mRNA LTSample Prep Kit (Illumina). These libraries were then sequenced using the Illumina sequencing platform (HiSeqTM 2500 or Illumina HiSeq X 10), and 125-bp and 150-bp paired-end reads were generated. Transcriptome sequencing was conducted by OE Biotech Co., Ltd., and clean reads were provided. The clean reads were mapped to the hg38 reference genome using hisat2 (version 2.1.0). The output BAM files were converted to SAM files using SAMtools 1.9. The final transcripts per million (TPM) values were obtained using Stringtie 1.3.5.

### In vivo metabolic labeling of GPI intermediates

The process of labeling experiments followed a previous protocol (Wang et al., 2020). For mannose labeling, ∼2 × 10^6^ PIGS-KO, PIGS-HRD1-DKO, and PIGS-HRD1-CD55-TKO cells, and PIGS-HRD1-CD55-TKO cells stably expressing HA-CD55 were precultured in normal medium overnight, washed with wash medium (glucose-free DMEM buffered with 20 mM Hepes, pH 7.4), and incubated for 1 h at 37°C in 1 ml of reaction medium (wash medium supplemented with 10% dialyzed FBS (Gibco), 10 μg ml^−1^ tunicamycin (Wako), and 100 μg ml^−1^ glucose). [2-^3^H] Mannose (American Radiolabeled Chemicals) was added to 25 Ci ml^−1^ and the cells were incubated for 1 h at 37°C in 5% CO_2_. The cells were pelleted and washed with 1 ml of cold PBS. Radiolabeled GPIs were extracted with 1-butanol, separated by high-performance thin-layer chromatography (HPTLC; Merck), and visualized using an FLA 7000 analyzer (Fujifilm).

To detect GlcN-PI or GlcN-(acyl)-PI after inositol-labeling, PIGW-KO, PIGS-KO, PIGS-HRD1-DKO, PIGS-HRD1-ARV1-TKO, PIGS-HRD1-CD55-TKO, and HA-CD55 rescued cells were washed with inositol-free DMEM and then incubated in 1 ml of reaction medium B (inositol-free DMEM buffered with 20 mM Hepes, pH 7.4) supplemented with 10% dialyzed FBS in the presence of 10 μCi of myo-[2-^3^H] inositol (PerkinElmer) for 24 h. After metabolic labeling, the cells were washed twice with 1 ml of cold PBS and pelleted by centrifugation. Lipids and radiolabeled GPIs were extracted with 1-butanol partitioning, separated by HPTLC (Merck), and visualized using an FLA 7000 analyzer.

### Proximity labeling assay

For biotin labeling, the major process followed the protocol described by Alice Ting’s group (Branon et al., 2018).PIGS-HRD1-CD55-TKO cells stably expressing TurboID-CD55(C), TurboID-CD48(C), or TurboID-CD59(C) were seeded in 5 × 15 cm dishes and cultured in normal medium to reach 100% confluence. From a 100 mM biotin stock in DMSO, we diluted biotin directly into serum-containing cell culture medium to the desired final concentration of 500 µM biotin. Labeling was stopped after 2 h by transferring the cells to ice and washing them five times with ice-cold PBS. The cells were harvested and washed with PBS and lysed in 5 ml RIPA lysis buffer by gentle pipetting and rotating for 1 h at 4°C. Lysates were clarified by centrifugation at 21,600 × *g* for 10 min at 4°C, followed by collection of the supernatant in a new tube. To enrich biotinylated material from samples, 250 µl StrepTactin Sepharose (28-9355-99; GE) was added to the supernatant and rotated for 1 h at 4°C. The Sepharose was washed twice with 1 ml of RIPA lysis buffer, once with 1 ml of 1 M KCl, once with 1 ml of 0.1 M Na_2_CO_3_, once with 1 ml of 2 M urea in 10 mM Tris-HCl (pH 8.0), and twice with 1 ml RIPA lysis buffer. The beads were then resuspended in 0.5 ml fresh RIPA lysis buffer, transferred to a new Eppendorf tube, and stored at −80°C for further processing and preparation for liquid chromatography–tandem mass spectrometry (LC-MS/MS) analysis.

### MS analysis

To prepare samples for MS analysis, proteins bound to streptavidin beads were washed twice with 200 µl of 50 mM Tris HCl buffer (pH 7.5) followed by two washes with 0.8 M urea/40 mM NH_4_HCO_3_ buffer. The beads were incubated with 200 µl of 0.8 M urea/40 mM NH_4_HCO_3_ containing 10 mM DTT incubated for 1 h at 37°C with shaking, followed by alkylation with 10 mM iodoacetamide for 45 min in the dark at 37°C with shaking. Then, 4 µg trypsin was added to the sample and another 4 µg trypsin was added to the mixture overnight at 37°C with shaking. After overnight digestion, the samples were acidified (to pH < 3) by addition of formic acid (FA). The samples were desalted on C18 StageTips and evaporated to dryness in a vacuum concentrator.

For tandem mass tag (TMT) labeling, desalted peptides were labeled with TMT (6-plex) reagents according to the manufacturer’s protocol. Peptides were reconstituted in 100 µl of 50 mM Hepes. Each 0.8 mg vial of TMT reagent was reconstituted in 41 µl of anhydrous acetonitrile (MeCN) and added to the corresponding peptide sample for 1 h at RT. TMT labeling reactions were quenched with 8 µl of 5% hydroxylamine at RT for 15 min with shaking, evaporated to dryness in a vacuum concentrator, and desalted on C18 StageTips. For each TMT 6-plex cassette, 50% of the sample was fractionated by basic pH reverse phase using StageTips, while the other 50% of each sample was reserved for LC-MS analysis using a single-shot, long gradient. One StageTip was prepared per sample using two plugs of styrene divinylbenzene (3M) material. The StageTips were conditioned two times with 50 µl of 100% methanol, followed by 50 µl of 50% MeCN/0.1% FA, and two times with 75 µl of 0.1% FA. The sample, resuspended in 100 µl of 0.1% FA, was loaded onto the stage tips and washed with 100 µl of 0.1% FA. Subsequently, the sample was washed with 60 µl of 20 mM NH_4_HCO_2_/2% MeCN, and this wash was saved and added to fraction 1. The sample was then eluted from StageTip using the following concentrations of MeCN in 20 mM NH_4_HCO_2_: 10, 15, 20, 25, 30, 40, and 50%. For a total of six fractions, the 10 and 40% (fractions 2 and 7) elutions were combined, as well as the 15 and 50% elutions (fractions 3 and 8). The six fractions were dried by vacuum centrifugation.

The dried peptides were resuspended in 15 μl of 2% MeCN and 0.1% FA solution and then analyzed using an EASY-nLC 1200 system (Thermo Fisher Scientific) coupled with a high-resolution Orbitrap Fusion Lumos spectrometer (Thermo Fisher Scientific). Each injection volume was 3 μl. The samples were first separated on an EASY-nLC 1200 system with an RSLC C18 column (1.9 μm × 100 μm × 20 cm) packed in-house.

### Statistical analysis

Prism Software (GraphPad Prism 8.0 software; GraphPad software Inc.) was used to generate diagrams and to calculate statistical significance. The flow cytometry data were normalized to the geometric MFI of the respective control sample (EV transfected, KO, or WT cells) to calculate fold changes. All diagrams depict mean values ± SD; statistical analysis was determined using the one-way ANOVA followed by Dunnett’s multiple comparisons test (sample ≥3) or two-sided Student’s *t* test to evaluate comparisons between three individual experiments.

### Online supplemental material

Fig. S1 shows CRISPR–Cas9 pooled screening to identify regulators of GPI biosynthesis. Fig. S2 presents the PIG gene expression and protein levels in PIGS-HRD1-CD55-TKO cells. Fig. S3 shows the expression of GPI-APs involved in GPI biosynthesis. Fig. S4 presents function of the GPI attachment signal peptide of CD55 and interaction of ARV1 with CD55. Fig. S5 shows analysis of the interaction of ARV1 with CD55 or PIGQ. Table S1 lists primers used in this study. Table S2 shows GPI-AP mRNA levels in HEK293 cells (TPM by RNA-seq). Table S3 shows TurboID labeling results. The CRISPR screening data are provided in Data S1. The RNA-seq data are available in Data S2 and from the GEO database under accession number GSE184822 (RNA-seq of PIGS-KO, PIGS-HRD1-DKO, PIGS-HRD1-CD55-TKO, and PIGS-HRD1-CD55-TKO+HA-CD55 cells). The MS proteomics data have been deposited to the ProteomeXchange Consortium via the PRIDE partner repository with the dataset identifier PXD028707 (project name: Determination of proteins labeling by Turbo-ID in PIGS-HRD1-CD55-TKO cells). Uncropped WB images in the main and supplemental figures are provided in Source Data files. All other data that support the findings of this study are available from the corresponding author upon reasonable request. Open Access funding provided by Osaka University.

## Supplementary Material

Table S1 lists primers used in this study.

Table S2 shows GPI-AP mRNA levels in HEK293 cells (TPM by RNA-seq).

Table S3 shows TurboID results.

Data S1 shows CRISPR screening data.

Data S2 shows RNA-seq data.

SourceData F3 is the source file for Fig. 3.

SourceData F4 is the source file for Fig. 4.

SourceData F5 is the source file for Fig. 5.

SourceData F6 is the source file for Fig. 6.

SourceData F7 is the source file for Fig. 7.

SourceData FS3 is the source file for Fig. S3.

SourceData FS4 is the source file for Fig. S4.

SourceData FS5 is the source file for Fig. S5.
