# Supplementary material for: Accumulated precursors of specific GPI-anchored proteins upregulate GPI biosynthesis with ARV1
Source: J Cell Biol. 2023 Feb 24;222(5):e202208159. doi: 10.1083/jcb.202208159 (PMC9997660; doi:10.1083/jcb.202208159)
Supplement: Table S1 — lists primers used in this study. [file JCB_202208159_TableS1.docx]

**Table S1.**

List of primers used in this study

| **list of primers used in this study** | | |
| --- | --- | --- |
| **Primer name** | **Sequence (5'-3')** | **purpose** |
| **CRISPR sgRNA primers** | | |
| sg-HRD1-F | caccCAAGTATGTGCTGCACTCCG | Cloning sgRNA to px330-EGFP |
| sg-HRD1-R | aaacCGGAGTGCAGCACATACTTG | Cloning sgRNA to px330-EGFP |
| sg-SRD5A3-F1 | caccGAGTGCTCGGCCTCCGCCCA | Cloning sgRNA to px330-EGFP |
| sg-SRD5A4-R1 | aaacTGGGCGGAGGCCGAGCACTC | Cloning sgRNA to px330-EGFP |
| sg-SRD5A3-F2 | caccGACATCAAAGGCTCGGCAGG | Cloning sgRNA to px330-EGFP |
| sg-SRD5A4-R2 | aaacCCTGCCGAGCCTTTGATGTC | Cloning sgRNA to px330-EGFP |
| sg-ARV1-F1 | caccGATGCACCTGTACTGGCAGG | Cloning sgRNA to px330-EGFP |
| sg-ARV1-R1 | ﻿aaacCCTGCCAGTACAGGTGCATC | Cloning sgRNA to px330-EGFP |
| sg-ARV1-F2 | caccGCACACCGTGGTTATAGTCT | Cloning sgRNA to px330-EGFP |
| sg-ARV1-R2 | ﻿aaacAGACTATAACCACGGTGTGC | Cloning sgRNA to px330-EGFP |
| sg-CD55-F1 | caccCTTGTACGGCCTTCCAAAGC | Cloning sgRNA to px330-EGFP |
| sg-CD55-R1 | aaac﻿GCTTTGGAAGGCCGTACAAG | Cloning sgRNA to px330-EGFP |
| sg-CD55-F2 | caccAATGGTCAGATTGATGTACC | Cloning sgRNA to px330-EGFP |
| sg-CD55-R2 | aaac﻿GGTACATCAATCTGACCATT | Cloning sgRNA to px330-EGFP |
| sg-SPPL3-F1 | caccGGAACATCACGCCCAACATT | Cloning sgRNA to px330-EGFP |
| sg-SPPL3-R1 | ﻿aaacAATGTTGGGCGTGATGTTCC | Cloning sgRNA to px330-EGFP |
| sg-SPPL3-F2 | caccGACGTTGCTATTGAAGATGT | Cloning sgRNA to px330-EGFP |
| sg-SPPL3-R2 | ﻿aaacACATCTTCAATAGCAACGTC | Cloning sgRNA to px330-EGFP |
| sg-CLPTM1L-F1 | caccGCCCACCACCAAGCTGGTGA | Cloning sgRNA to px330-EGFP |
| sg-CLPTM1L-R1 | aaacTCACCAGCTTGGTGGTGGGC | Cloning sgRNA to px330-EGFP |
| sg-CLPTM1L-F2 | caccGCAGCTTGGGCCGCCGCGCC | Cloning sgRNA to px330-EGFP |
| sg-CLPTM1L-R2 | aaacGGCGCGGCGGCCCAAGCTGC | Cloning sgRNA to px330-EGFP |
| sg-CD59-F1 | caccGCTGTTTTGCAGTCAGCAGT | Cloning sgRNA to px330-EGFP |
| sg-CD59-R1 | ﻿aaacACTGCTGACTGCAAAACAGC | Cloning sgRNA to px330-EGFP |
| sg-CD59-F2 | caccGCTGTTCGTTAAAGTTACAC | Cloning sgRNA to px330-EGFP |
| sg-CD59-R2 | ﻿aaacGTGTAACTTTAACGAACAGC | Cloning sgRNA to px330-EGFP |
| sg-PRNP-F1 | caccGAACCAGCATCCAGCAGCCA | Cloning sgRNA to px330-EGFP |
| sg-PRNP-R1 | ﻿aaacTGGCTGCTGGATGCTGGTTC | Cloning sgRNA to px330-EGFP |
| sg-PRNP-F2 | caccGGTGGCTGGGGTCAAGGAGG | Cloning sgRNA to px330-EGFP |
| sg-PRNP-R2 | ﻿aaacCCTCCTTGACCCCAGCCACC | Cloning sgRNA to px330-EGFP |
| sg-GPC4-F1 | caccGCCAGGGTGCAGAGAAGCGC | Cloning sgRNA to px330-EGFP |
| sg-GPC4-R1 | ﻿aaacGCGCTTCTCTGCACCCTGGC | Cloning sgRNA to px330-EGFP |
| sg-GPC4-F2 | ﻿caccGTTGAAACGTTACTACGTGG | Cloning sgRNA to px330-EGFP |
| sg-GPC4-R2 | ﻿aaacCCACGTAGTAACGTTTCAAC | Cloning sgRNA to px330-EGFP |
| sg-CD109-F1 | caccGTGCACCGCCGCGCTGGCCG | Cloning sgRNA to px330-EGFP |
| sg-CD109-R1 | aaac﻿CGGCCAGCGCGGCGGTGCAC | Cloning sgRNA to px330-EGFP |
| sg-CD109-F2 | caccGTTGGATCAAATTTGATTTG | Cloning sgRNA to px330-EGFP |
| sg-CD109-R1 | ﻿aaacGTGCACCGCCGCGCTGGCCG | Cloning sgRNA to px330-EGFP |
| sg-LY6E-F1 | caccGATGGGGCAGGCCGGGGAAC | Cloning sgRNA to px330-EGFP |
| sg-LY6E-R1 | aaac﻿GTTCCCCGGCCTGCCCCATC | Cloning sgRNA to px330-EGFP |
| sg-LY6E-F2 | caccGTTGTCCTGGTCGGAGCAGA | Cloning sgRNA to px330-EGFP |
| sg-LY6E-R2 | ﻿aaacTCTGCTCCGACCAGGACAAC | Cloning sgRNA to px330-EGFP |
| **cDNA Cloning Primers** | | |
| HRD1-F | aaaaGTCGACCACCATGTTCCGCACGGCAGTGAT | Cloning HRD1 to pME-3HA |
| HRD1-R | aaaaGCGGCCGCTTAGTGGGCAACAGGAGACT |  |
| CLPTM1L-F | aaaaGAATTCCACCATGTGGAGCGGCCGCAGCTC | Cloning CLPTM1L to pLIB2-BSD |
| CLPTM1L-R | aaaaCTCGAGTCAGTCCGTGTGGGGCGCCC |  |
| SPPL3-F | aaaa﻿GAATTCCACCATGGCGGAGCAGACCTACTC | Cloning SPPL3 to pME-3HA |
| SPPL3-R | aaaaACGCGTTCATACTTCCAGGAATCGGG |  |
| ARV1-F | aaaaGTCGACCACCATGGGCAACGGCGGGCGGAG | Cloning ARV1 to pME-3Flag |
| ARV1-R | aaaaGCGGCCGCTCAGAAGTCCTGAGATTTAA |  |
| SRD5A- F | aaaaGTCGACCACCATGGCTCCCTGGGCGGAGGC | Cloning SRD5A3 to pLIB2-BSD |
| SRD5A-R | aaaaGCGGCCGCTTAAAACAAAAATGGTAGGA |  |
| mCD55-F | aaaaCTCGAGGACTGCGGCCCACCTCCAGA | Cloning mCD55 to pME-HA |
| mCD55-R | aaaaGCGGCCGCCTATGTCAAGTAGCCAATGA |  |
| 3xHA-TurboID-F | aaaaAAGCTTTACCCGTATGATGTTCCGGA | Cloning 3XHA-TurboID to pLIB2-BSD |
| 3xHA-TurboID-R | aaaaCTCGAGCTTTTCGGCAGACCGCAGAC |  |
| CD55(C)-F | aaaaCTCGAGTCAGGTACTACCCGTCTTCT | Cloning CD55(C) to pLIB2-BSD-3xHA-TurboID |
| CD55(C)-R | aaaaGCGGCCGCCTAAGTCAGCAAGCCCATGGT |  |
| CD48(C)-F | aaaaCTCGAGTCCTTTGGAGTAGAATGGAT | Cloning CD48(C) to pLIB2-BSD-3xHA-TurboID |
| CD48(C)-R | aaaaGCGGCCGCTCAGGTAAGTAACAGGCCAA |  |
| CD59(C)-F | aaaaCTCGAGAATGGTGGGACATCCTTATC | Cloning CD59(C) to pLIB2-BSD-3xHA-TurboID |
| CD59(C)-R | aaaaGCGGCCGCCTAAGTCAGCAAGCCCATGG |  |
| PRNP(C)-F | aaaaCTCGAGTCGAGCATGGTCCTCTTCTC | Cloning PRNP(C) to pLIB2-BSD-3xHA-TurboID |
| PRNP(C)-R | aaaaGCGGCCGCCTATCCCACTATCAGGAAGA |  |
| CD55 (T35-96)-F | AGCTGCGAGGTGCCAACAAGGCTAAATTCT | pME-HA-CD55(T35-96) |
| CD55 (T35-96)-R | CTCGAGGCCGGCAAGCTTTCCAGCTGCGTA |  |
| CD55 (T35-159)-F | AAGAAATCATGCCCTAATCCGGGAGAAATA | pME-HA-CD55(T35-159) |
| CD55 (T35-159)-R | CTCGAGGCCGGCAAGCTTTCCAGCTGCGTA |  |
| CD55 (T35-221)-F | GAAATTTATTGTCCAGCACCACCACAAATT | pME-HA-CD55(T35-221) |
| CD55 (T35-221)-R | CTCGAGGCCGGCAAGCTTTCCAGCTGCGTA |  |
| CD55 (T35-285)-F | AAATCTCTAACTTCCAAGGTCCCACCAACA | pME-HA-CD55(T35-285) |
| CD55 (T35-285)-R | CTCGAGGCCGGCAAGCTTTCCAGCTGCGTA |  |
| CD55 (T35-349)-F | TAGGCGGCCGCTAGACTAGTCTAGAGAAAA | pME-HA-CD55(T35-349) |
| CD55 (T35-349)-R | CTCGAGGCCGGCAAGCTTTCCAGCTGCGTA |  |
| CD55 (T285-349)-F | GGAACCACTTCAGGTACTACCCGTCTTCTA | pME-HA-CD55(T285-349) |
| CD55 (T285-349)-R | TCCTCTGCATTCAGGTGGTGGGCCACTCCA |  |
| CD55 (T349-381)-F | TAGGCGGCCGCTAGACTAGTCTAGAGAAAA | pME-HA-CD55(T349-381) |
| CD55 (T349-381)-R | TCCTCTGCATTCAGGTGGTGGGCCACTCCA |  |
| CD55 (T353-361)-F | CACACGTGTTTCACGTTGACAGGTTTGCTT | pME-HA-CD55(T353-361) |
| CD55 (T353-361)-R | AGTGGTTCCACTTCCTTTATTTGGGGTTGT |  |
| CD55 (T362-381)-F | TAGGCGGCCGCTAGACTAGTCTAGAGAAAA | pME-HA-CD55(T362-381) |
| CD55 (T362-381)-R | CCCAGATAGAAGACGGGTAGTACCTGAAGT |  |
| CD55 (S353G)-F | GACGGGTAGTACCTCCAGTGGTTCCACTTCCTTTATTTGGGG | pME-HA-CD55(S353G) |
| CD55 (S353G)-R | CCCCAAATAAAGGAAGTGGAACCACTGGAGGTACTACCCGTC |  |
| CD55 (S353P)-F | GACGGGTAGTACCTGGAGTGGTTCCACTTCC | pME-HA-CD55(S353P) |
| CD55 (S353P)-R | GGAAGTGGAACCACTCCAGGTACTACCCGTC |  |
| GFP-CD55(C) S353P-F | GACGGGTAGTACCTGGGTCGACCTTGTACAG | pME-GFP-CD55(C) S353P |
| GFP-CD55(C) S353P-R | CTGTACAAGGTCGACCCAGGTACTACCCGTC |  |
| CD55 (L370A)-F | GTTACTACCGTCCCAAGCGCACCTGTCAACGTGAAACACGT | pME-HA-CD55(L370A) |
| CD55 (L370A)-R | ACGTGTTTCACGTTGACAGGTGCGCTTGGGACGCTAGTAAC |  |
| CD55 (G372A)-F | CCCATGGTTACTAGCGTATGAAGCAAACCTGTCAACGTGAAACACG | pME-HA-CD55(G372A) |
| CD55 (G372A)-R | CGTGTTTCACGTTGACAGGTTTGCTTCATACGCTAGTAACCATGGG |  |
| CD55 (L373V)-F | CCATGGTTACTAGCGTCGCAAGCAAACCTGTCAAC | pME-HA-CD55(L373V) |
| CD55 (L373V)-R | GTTGACAGGTTTGCTTGCGACGCTAGTAACCATGG |  |
| CD55 (G378Y)-F | GGCCGCCTAAGTCAGCAAATACATGGTTACTAGCGTCCC | pME-HA-CD55(G378Y) |
| CD55 (G378Y)-R | GGGACGCTAGTAACCATGTATTTGCTGACTTAGGCGGCC |  |
| CD55-CD59(C)-F | GCCTGGAGCCTTCATCCCTAGGCGGCCGCTAGACTAGT | pME-HA-CD55-CD59(C) |
| CD55-CD59(C)-R | TGCTGCCAGAAATGGAGTGTGCCCAGATAGAAGACGGG |  |
| CD59-CD55(C)-F | AAAACAGTTCTTCTGCTGGTGACGTGTTTCACGTTGACAGG | pME-HA-CD59-CD55(C) |
| CD59-CD55(C)-R | CTCTGATAAGGATGTCCCACCATTTCCTTTATTTGGGGTTGTTT |  |
| CD48 (L238H)-F | AGTAACAGGCCATGAATGGTGGGCACCGTG | pME-HA-CD48(L378H) |
| CD48 (L238H)-R | CACGGTGCCCACCATTCATGGCCTGTTACT |  |
| ARV1-F1 | aaaaGTCGACATGGGCAACGGCGGGCGGA | pME-N6myc-ARV1 |
| ARV1-R1 | aaaaGCGGCCGCTAGAAGTCCTGAGATTTAA |  |
| PIGH-F | aaaaGTCGACgaggatgagcggagcttttc | pME-N6myc-PIGH |
| PIGH-R | aaaaTCTAGAtcatgggcttgttgatgtgg |  |
| PIGQ-F | aaaaGTCGACGTGCTCAAGGCCTTCTTCCC | pME-3Flag-PIGQ |
| PIGQ-R | aaaaGCGGCCGCCTACAAAGCGACCTCTCCAG |  |
| PIGQ-F IN-FUSION | gctctaaaagctgcggaattcCACCATGGTGCTCAAGGCC | pME-PIGQ-NTC |
| PIGQ-R IN-FUSION | ggatcctccgccgccctcgagCAAAGCGACCTCTCCAGGG |  |
| PIGH-F IN-FUSION | gctctaaaagctgcggaattcCACCATGGAGGATGAGCGG | pME-PIGH-CTC |
| PIGH-R IN-FUSION | ggatcctccgccgccctcgagTGGGCTTGTTGATGTGGCTT |  |
| ARV1-F2 | aaaaCTCGAGGGCAACGGCGGGCGGAGCGG | pME-CTC-ARV1 |
| ARV1-R2 | aaaaGCGGCCGCTCAGAAGTCCTGAGATTTAA |  |
| ARV1-F3 | aaaaGTCGACCACCatgggcaacggcgggcggag | pME-ARV1-CTC |
| ARV1-R3 | aaaaCTCGAGgaagtcctgagatttaaaga |  |
| CD55(C)-F1 | aaaaGTCGACTCAGGTACTACCCGTCTTCT | pME-NTC-CD55(C) |
| CD55(C)-R1 | aaaaGCGGCCGCCTAAGTCAGCAAGCCCATGGT |  |
| CD55(C)-F2 | aaaaCTCGAGTCAGGTACTACCCGTCTTCT | pME-ssGFP-CD55(C) |
| CD55(C)-R2 | aaaaGCGGCCGCCTAAGTCAGCAAGCCCATGGT |  |
| PLET1(C)-F | aaaaGTCGACagcagccccatcacagaggc | pME-ssGFP-PLET1(C) |
| PLET1(C)-R | aaaaGCGGCCGCttagaagagaagtgtgctgg |  |
| BST1(C)-F | aaaaGTCGACgccccaagtctttatacaga | pME-ssGFP-BST1(C) |
| BST1(C)-R | aaaaGCGGCCGCttacagttgagtcctggaag |  |
| BST2(C)-F | aaaaGTCGACagctccgctgcggcgcccca | pME-ssGFP-BST2(C) |
| BST2(C)-R | aaaaGCGGCCGCtcactgcagcagagcgctga |  |
| CD58(C)-F | aaaaGTCGACattttgacaacctgtatccc | pME-ssGFP-CD58(C) |
| CD58(C)-R | aaaaGCGGCCGCTCAATTGGAGTTGGTTCTGT |  |
| TFPI(C)-F | aaaaGTCGACAATGATGGTTGGAAGAATGC | pME-ssGFP-TFPI(C) |
| TFPI(C)-R | aaaaGCGGCCGCCTAACATAGGCATGAAATGC |  |
