# Supplementary material for: Accumulated precursors of specific GPI-anchored proteins upregulate GPI biosynthesis with ARV1
Source: J Cell Biol. 2023 Feb 24;222(5):e202208159. doi: 10.1083/jcb.202208159 (PMC9997660; doi:10.1083/jcb.202208159)
Supplement: Table S2 — shows GPI-AP mRNA levels in HEK293 cells (TPM by RNA-seq). [file JCB_202208159_TableS2.docx]

**Table S2.**

GPI-AP mRNA levels in HEK293 cells (TPM by RNASeq)

| Gene | TPM |
| --- | --- |
| PGRMC1 | 102.7065 |
| LY6E | 87.56313 |
| HSPA5 | 71.70255 |
| RAB5IF | 32.33833 |
| PRNP | 26.359 |
| EMC10 | 22.46 |
| BST2 | 21.79953 |
| GPC4 | 17.2451 |
| CD59 | 17.24233 |
| GPC1 | 14.90203 |
| GAS1 | 14.16447 |
| EFNA3 | 12.94127 |
| RTN4RL2 | 10.217 |
| CD109 | 9.054205 |
| GPC3 | 8.47488 |
| HYAL2 | 8.25609 |
| CD55 | 8.212023 |
| FAM241B | 7.564888 |
| RGMA | 7.464783 |
| RTN4R | 7.284023 |
| RTN4RL1 | 6.64749 |
| MELTF | 6.418393 |
| MOXD1 | 5.84535 |
| EFNA4 | 5.84316 |
| MICA | 5.420328 |
| NRN1 | 5.167253 |
| EFNA1 | 5.074095 |
| MMP17 | 5.02618 |
| ULBP3 | 4.547013 |
| SEMA6A | 3.767053 |
| NCAM1 | 3.704225 |
| MDGA1 | 3.426498 |
| LPL | 2.987728 |
| ACHE | 2.928713 |
| LYPD1 | 2.838403 |
| RGMB | 2.744788 |
| EFNA5 | 2.519358 |
| ALPP | 2.41164 |
| FOLR1 | 2.409398 |
| ULBP2 | 2.403795 |
| CACNA2D1 | 2.373773 |
| ULBP1 | 2.298385 |
| LYPD6 | 1.666303 |
| DMKN | 1.538488 |
| GPC5 | 1.533104 |
| LYPD6B | 1.40829 |
| FAM155B | 1.30889 |
| ALPL | 1.267172 |
| SMPDL3B | 1.252741 |
| GPC2 | 1.251063 |
| CACNA2D2 | 1.182899 |
| GFRA2 | 1.157547 |
| ALPPL2 | 1.057479 |
| CNTN1 | 1.055537 |
| CPM | 1.024725 |
| RECK | 0.956251 |
| CD58 | 0.940047 |
| PLAUR | 0.88994 |
| EFNA2 | 0.876374 |
| GFRA1 | 0.866902 |
| CD52 | 0.833046 |
| SEMA7A | 0.778048 |
| LY6K | 0.730406 |
| CDH13 | 0.686542 |
| CACNA2D3 | 0.674904 |
| TFPI | 0.649829 |
| CNTFR | 0.623782 |
| LYPD3 | 0.572209 |
| GPC6 | 0.489958 |
| IGSF21 | 0.472351 |
| CD24 | 0.463998 |
| LYNX1 | 0.445118 |
| MMP25 | 0.437028 |
| CCDC180 | 0.370075 |
| NTNG2 | 0.313218 |
| PRSS8 | 0.286543 |
| NT5E | 0.270767 |
| CD14 | 0.260698 |
| TNFRSF10C | 0.245193 |
| TECTA | 0.245002 |
| RAET1G | 0.220691 |
| PSCA | 0.219729 |
| LYPD5 | 0.14842 |
| NRN1L | 0.114483 |
| LY6G6C | 0.114005 |
| GFRA3 | 0.097478 |
| MSLN | 0.091082 |
| LY6G6D | 0.084094 |
| IZUMO1R | 0.078584 |
| NYX | 0.077396 |
| RAET1L | 0.061989 |
| TSPAN1 | 0.05715 |
| BST1 | 0.050257 |
| ENG | 0.050015 |
| GPIHBP1 | 0.048912 |
| THY1 | 0.035171 |
| CPO | 0.034444 |
| ITLN1 | 0.034394 |
| LSAMP | 0.027245 |
| SPRN | 0.021256 |
| XPNPEP2 | 0.019662 |
| VNN2 | 0.018269 |
| OTOA | 0.018231 |
| PRSS21 | 0.017482 |
| BCAN | 0.017357 |
| LRRC6 | 0.01672 |
| DPEP2 | 0.013441 |
| CA4 | 0.013082 |
| DPEP1 | 0.012743 |
| CD177 | 0.012741 |
| ALPI | 0.012456 |
| CNTN2 | 0.011681 |
| CD160 | 0.007707 |
| CEACAM6 | 0.007181 |
| CFC1 | 0.006527 |
| ENPP6 | 0.005176 |
| ART1 | 0.003954 |
| GLIPR1L1 | 0.002922 |
| VTCN1 | 0.002686 |
| CACNA2D4 | 0.002059 |
| NTM | 0.001792 |
| CNTN5 | 0.001217 |
| LY6L | 0 |
| SIRPB1 | 0 |
| HJV | 0 |
| PLET1 | 0 |
| ART3 | 0 |
| ART4 | 0 |
| CD48 | 0 |
| CEACAM5 | 0 |
| CEACAM7 | 0 |
| CEACAM8 | 0 |
| CNTN3 | 0 |
| CNTN4 | 0 |
| CNTN6 | 0 |
| DPEP3 | 0 |
| FCGR3B | 0 |
| FOLR2 | 0 |
| GFRA4 | 0 |
| GML | 0 |
| GP2 | 0 |
| LY6D | 0 |
| LY6H | 0 |
| LYPD2 | 0 |
| LYPD4 | 0 |
| LYPD8 | 0 |
| MDGA2 | 0 |
| NEGR1 | 0 |
| NTNG1 | 0 |
| OMG | 0 |
| OPCML | 0 |
| PRND | 0 |
| PRSS41 | 0 |
| SPACA4 | 0 |
| SPAM1 | 0 |
| TDGF1 | 0 |
| TECTB | 0 |
| TEX101 | 0 |
| TREH | 0 |
| UMOD | 0 |
| VNN1 | 0 |
| VNN3 | 0 |
