# Supplementary material for: Accumulated precursors of specific GPI-anchored proteins upregulate GPI biosynthesis with ARV1
Source: J Cell Biol. 2023 Feb 24;222(5):e202208159. doi: 10.1083/jcb.202208159 (PMC9997660; doi:10.1083/jcb.202208159)
Supplement: Table S3 — shows TurboID results. [file JCB_202208159_TableS3.docx]

**Table S3.**

TurboID results

| Genes | FoldChange(CD55/CD59) |
| --- | --- |
| SGMR2 | 2.317840509 |
| PCAT1 | 2.196039391 |
| AG10B | 2.029785634 |
| P3H3 | 2.00089091 |
| SPCS2 | 1.995895162 |
| HYOU1 | 1.979278104 |
| COEA1 | 1.977195889 |
| ARV1 | 1.969838734 |
| RS28 | 1.951636038 |
| LAMC1 | 1.931996621 |
| DNJC3 | 1.871671922 |
| LAMB1 | 1.837937564 |
| MA1A1 | 1.804901379 |
| CKAP4 | 1.743553173 |
| PDIA4 | 1.708188023 |
| DJB12 | 1.681886103 |
| SERPH | 1.676982717 |
| GANAB | 1.642886841 |
| LRC59 | 1.630232732 |
| P4HA2 | 1.606554866 |
| LAMB2 | 1.602769985 |
| CREL2 | 1.602344077 |
| SARAF | 1.583023734 |
| HYAL2 | 1.575525978 |
| DJB11 | 1.573079471 |
| LMAN1 | 1.568937851 |
| TMTC3 | 1.556580486 |
| RN185 | 1.556243605 |
| CSTN1 | 1.554729619 |
| SPCS3 | 1.541995946 |
| SOAT1 | 1.532284264 |
| PDIA3 | 1.520490454 |
| MESD | 1.512440632 |
| DHB12 | 1.490775856 |
| PLOD3 | 1.476841698 |
| PLCA | 1.473869412 |
| ERD21 | 1.461362312 |
| GT252 | 1.460401201 |
| B3GLT | 1.45021119 |
| P3H1 | 1.448615869 |
| TMED2 | 1.442300992 |
| RNT2 | 1.434651421 |
| SORT | 1.431060548 |
| DNJA1 | 1.428936753 |
| ARSB | 1.423357016 |
| IKIP | 1.419929157 |
| DHCR7 | 1.416349163 |
| TXD12 | 1.414687632 |
| EDEM2 | 1.414256914 |
| WNT5B | 1.413876092 |
| SRP09 | 1.399045992 |
| PDIA6 | 1.395356037 |
| CO5A1 | 1.394829612 |
| STT3B | 1.387444435 |
| NCLN | 1.383503055 |
| CC134 | 1.382163223 |
| CO6A2 | 1.382012632 |
| FPRP | 1.380233972 |
| NOMO3 | 1.37935799 |
| PDIA1 | 1.370577503 |
| LBR | 1.366849961 |
| ADPGK | 1.364987844 |
| JMJD8 | 1.363113889 |
| TXD16 | 1.356890421 |
| 15-Sep | 1.354568663 |
| EDEM3 | 1.353412256 |
| TMED7 | 1.350999542 |
| ERO1B | 1.347257838 |
| PO210 | 1.344118741 |
| DNJB9 | 1.338779255 |
| FKB10 | 1.338181818 |
| ERLN2 | 1.33778149 |
| GLU2B | 1.336663545 |
| LRP6 | 1.331218632 |
| NICA | 1.330478513 |
| CNPY3 | 1.330456803 |
| LRP5 | 1.330254815 |
| DJC10 | 1.329416144 |
| SUMF1 | 1.323947896 |
| CRTAP | 1.323142463 |
| PCSK6 | 1.317240478 |
| ERAP2 | 1.303985425 |
| FKBP9 | 1.302916969 |
| TXND5 | 1.301603676 |
| GLCM | 1.300952582 |
| CLUS | 1.295588865 |
| ASPH | 1.293449566 |
| TMED5 | 1.290299708 |
| ELAV1 | 1.285198946 |
| MANF | 1.283803523 |
| TXD11 | 1.281109082 |
| MIA2 | 1.280166926 |
| ENPL | 1.273129588 |
| PLOD1 | 1.272993992 |
| P4HA1 | 1.270003213 |
| ATF6A | 1.2649584 |
| TRFM | 1.25884268 |
| PLGT3 | 1.254804966 |
| SC65 | 1.252296075 |
| ARSK | 1.250221634 |
| PIGO | 1.245896033 |
| RS6 | 1.244365893 |
| SCAP | 1.241238084 |
| S39A7 | 1.23738595 |
| ERP29 | 1.228570797 |
| CDK1 | 1.225386779 |
| PXDN | 1.225300047 |
| SUCO | 1.225273901 |
| TGO1 | 1.222639233 |
| COPB | 1.222437443 |
| RL24 | 1.222332671 |
| CCN1 | 1.222028824 |
| RL40 | 1.221021969 |
| AHSA1 | 1.218847601 |
| CO6A1 | 1.217594395 |
| FBX6 | 1.21659919 |
| CHLE | 1.207515242 |
| PIGB | 1.205215782 |
| GT251 | 1.204406388 |
| AMRP | 1.19875218 |
| ERAP1 | 1.198105839 |
| COIA1 | 1.197986307 |
| RL13 | 1.196310915 |
| ERLN1 | 1.193424201 |
| COPD | 1.191574059 |
| UGGG1 | 1.1901385 |
| MFGM | 1.188006246 |
| PLGT2 | 1.182710243 |
| FZD6 | 1.178772699 |
| ARSA | 1.175896086 |
| RS3A | 1.17560482 |
| HM13 | 1.175524529 |
| SPP2B | 1.175251081 |
| NUCB1 | 1.172682413 |
| SURF4 | 1.171082354 |
| ABD12 | 1.166117767 |
| COPG1 | 1.162594832 |
| PLOD2 | 1.160771899 |
| TMX1 | 1.1585772 |
| FUCO2 | 1.156597996 |
| EDEM1 | 1.156213129 |
| RCN2 | 1.153904503 |
| ERMP1 | 1.152782205 |
| RL27A | 1.152272282 |
| SEC63 | 1.15134518 |
| PPIB | 1.149650641 |
| CALR | 1.142970668 |
| E2AK3 | 1.142488384 |
| BIP | 1.141256051 |
| SUMF2 | 1.141193755 |
| DPM1 | 1.136345414 |
| RS8 | 1.136291391 |
| DIK1B | 1.134585692 |
| ERP44 | 1.133915373 |
| RL21 | 1.131806931 |
| PGLT1 | 1.130604658 |
| ATF6B | 1.129210798 |
| PIGT | 1.127617562 |
| AT2A2 | 1.126331839 |
| RPN1 | 1.12428006 |
| MAGT1 | 1.121124107 |
| RS3 | 1.12052352 |
| RL10 | 1.119799086 |
| TOIP2 | 1.118179484 |
| MLEC | 1.11758903 |
| FIBIN | 1.117514852 |
| VAS1 | 1.117213664 |
| RS26 | 1.116582658 |
| HS71B | 1.113635507 |
| AT131 | 1.11281647 |
| MOXD1 | 1.109795213 |
| CALX | 1.10930424 |
| UBAC2 | 1.101463081 |
| RS23 | 1.101156344 |
| P3H2 | 1.097222636 |
| SULF2 | 1.095160508 |
| ACATN | 1.091055561 |
| APLP2 | 1.087404173 |
| CGT | 1.085349488 |
| RL36A | 1.083794229 |
| FXRD2 | 1.083374739 |
| KPYM | 1.083302204 |
| ERN1 | 1.081704206 |
| TECR | 1.074854213 |
| S61A1 | 1.072692557 |
| RL6 | 1.07024228 |
| FKTN | 1.069968157 |
| AT1A1 | 1.068493151 |
| SG196 | 1.067601357 |
| HACD3 | 1.066001383 |
| SE1L1 | 1.0657648 |
| ADA10 | 1.064486943 |
| RTN4R | 1.063788223 |
| SULF1 | 1.0595 |
| DPA1 | 1.059453816 |
| OS9 | 1.057721765 |
| PDIA5 | 1.056808727 |
| UGGG2 | 1.056791747 |
| RCN1 | 1.056436567 |
| RL18 | 1.053861594 |
| ERLEC | 1.052912667 |
| A4 | 1.050884744 |
| COPA | 1.050576629 |
| PIGG | 1.050072728 |
| KTN1 | 1.047197687 |
| PIGU | 1.047087343 |
| IMB1 | 1.046292696 |
| SELN | 1.044862965 |
| NF2L1 | 1.042600123 |
| GPX8 | 1.03958632 |
| TMM33 | 1.03497306 |
| ITPR2 | 1.033839393 |
| TOR1B | 1.033680732 |
| SRPRA | 1.025033434 |
| STT3A | 1.02493892 |
| GT253 | 1.016458144 |
| OST48 | 1.013256258 |
| PLD3 | 1.012879139 |
| ASTRA | 1.012794406 |
| ARSG | 1.012222347 |
| POMT2 | 1.010180142 |
| STIM1 | 1.009782363 |
| SIL1 | 1.004326757 |
| SC11A | 1.001358661 |
| GPAA1 | 0.997931873 |
| NENF | 0.997544669 |
| TM39A | 0.997399109 |
| GPI8 | 0.99705284 |
| RL34 | 0.995138178 |
| S35B2 | 0.995050839 |
| CC50A | 0.993838887 |
| T106C | 0.991421973 |
| STIM2 | 0.979698702 |
| TMEDA | 0.971175453 |
| PRDX4 | 0.969922718 |
| CLGN | 0.967301355 |
| ULBP1 | 0.965118807 |
| OFUT1 | 0.960781591 |
| EXT2 | 0.959706563 |
| RENR | 0.954875953 |
| PGRC2 | 0.951307064 |
| RL4 | 0.949919796 |
| GALT2 | 0.94954955 |
| TMED9 | 0.948363411 |
| CD59 | 0.946422193 |
| 2B1F | 0.945282101 |
| TMX3 | 0.945178803 |
| HLAE | 0.943427508 |
| WLS | 0.940120943 |
| XXLT1 | 0.938863274 |
| TPST2 | 0.938802943 |
| HSP13 | 0.938317704 |
| EXTL3 | 0.937198597 |
| CAB45 | 0.935475567 |
| UCHL1 | 0.934009644 |
| CR3L2 | 0.932719495 |
| FKBP7 | 0.931719672 |
| TMED4 | 0.930503648 |
| LRC8E | 0.929872127 |
| ERO1A | 0.923722646 |
| MBTP1 | 0.91726792 |
| NPC1 | 0.91408107 |
| MOGS | 0.913864827 |
| PMGT2 | 0.907270974 |
| EST2 | 0.906163818 |
| ILVBL | 0.90306702 |
| 1C07 | 0.898200989 |
| CH60 | 0.874630303 |
| MA1A2 | 0.874606604 |
| DAD1 | 0.873780457 |
| HLAF | 0.8736053 |
| LMAN2 | 0.863825288 |
| PGAP1 | 0.863239129 |
| EXT1 | 0.857763527 |
| POMT1 | 0.853803095 |
| ITPR3 | 0.842717534 |
| OFUT2 | 0.834960437 |
| LMF2 | 0.832603615 |
| CALU | 0.824173765 |
| LRC8C | 0.805835072 |
| RPN2 | 0.794783491 |
| SC11C | 0.789698609 |
| MBRL | 0.782366428 |
| NUCB2 | 0.765743378 |
| ARSE | 0.762489029 |
| SELT | 0.755041849 |
| SL9A6 | 0.753511908 |
| CLCC1 | 0.742184989 |
| LMF1 | 0.720823171 |
| LMA2L | 0.71551891 |
| ERGI3 | 0.707040505 |
| TOR1A | 0.705079192 |
| BAP31 | 0.701098251 |
| CNPY2 | 0.695822426 |
| GPC3 | 0.690877747 |
| DJB14 | 0.669809528 |
| BAP29 | 0.589239443 |
| CCD47 | 0.564573332 |
| PRIO | 0.543082804 |

| Genes | FoldChange(CD48/CD59) |
| --- | --- |
| AG10B | 4.517149 |
| MA1A1 | 4.026849 |
| SGMR2 | 3.396398 |
| SARAF | 3.196009 |
| PCAT1 | 3.100827 |
| RS28 | 2.715464 |
| TMED2 | 2.680542 |
| LRC59 | 2.557227 |
| RN185 | 2.191444 |
| ARV1 | 2.138559 |
| HYAL2 | 2.109529 |
| LAMB1 | 1.982085 |
| RPN1 | 1.91023 |
| STT3A | 1.88328 |
| PLCA | 1.83455 |
| DJB12 | 1.820175 |
| SEC63 | 1.800308 |
| CKAP4 | 1.780587 |
| TMED9 | 1.767827 |
| COEA1 | 1.714898 |
| DHB12 | 1.712926 |
| STT3B | 1.665604 |
| LMAN1 | 1.654292 |
| P3H3 | 1.62272 |
| CSTN1 | 1.616362 |
| S39A7 | 1.615879 |
| SORT | 1.615187 |
| CCN1 | 1.568509 |
| HM13 | 1.563021 |
| ERMP1 | 1.558578 |
| GANAB | 1.543968 |
| LBR | 1.524471 |
| FZD6 | 1.521342 |
| DNJA1 | 1.509149 |
| LAMC1 | 1.503354 |
| DJB11 | 1.500565 |
| NICA | 1.49171 |
| SPCS3 | 1.488305 |
| WNT5B | 1.481606 |
| CRTAP | 1.474468 |
| CREL2 | 1.471606 |
| B3GLT | 1.459064 |
| AMRP | 1.449226 |
| IKIP | 1.438712 |
| SERPH | 1.437805 |
| FKBP9 | 1.435682 |
| DHCR7 | 1.428766 |
| RENR | 1.425462 |
| LRP5 | 1.420838 |
| ITPR2 | 1.420136 |
| CC50A | 1.415772 |
| GLU2B | 1.414221 |
| OST48 | 1.411684 |
| TXD12 | 1.411082 |
| TMED7 | 1.40647 |
| MESD | 1.400404 |
| STIM1 | 1.400382 |
| ADPGK | 1.399254 |
| RS3 | 1.397914 |
| ASPH | 1.389396 |
| CC134 | 1.386183 |
| ATF6A | 1.38197 |
| FPRP | 1.380437 |
| GT252 | 1.378696 |
| SCAP | 1.377373 |
| RNT2 | 1.372294 |
| SRP09 | 1.36918 |
| ABD12 | 1.368179 |
| VAS1 | 1.364032 |
| KTN1 | 1.362585 |
| GPX8 | 1.360967 |
| MAGT1 | 1.359683 |
| PDIA3 | 1.358192 |
| BAP31 | 1.357203 |
| NCLN | 1.352749 |
| PDIA4 | 1.351322 |
| RL13 | 1.3488 |
| E2AK3 | 1.347466 |
| MLEC | 1.345911 |
| TGO1 | 1.345486 |
| GPAA1 | 1.338281 |
| LAMB2 | 1.331393 |
| SPCS2 | 1.330868 |
| RS6 | 1.3217 |
| ARSB | 1.321448 |
| SUCO | 1.319788 |
| RL21 | 1.31588 |
| DNJC3 | 1.310124 |
| RL40 | 1.309816 |
| FBX6 | 1.303987 |
| SELN | 1.303472 |
| PO210 | 1.298528 |
| 2B1F | 1.294585 |
| HYOU1 | 1.294115 |
| LRP6 | 1.291378 |
| MIA2 | 1.290258 |
| SPP2B | 1.286473 |
| NUCB1 | 1.285956 |
| ERD21 | 1.283702 |
| RL24 | 1.277078 |
| HLAE | 1.269103 |
| RL18 | 1.265831 |
| ASTRA | 1.265165 |
| WLS | 1.265022 |
| JMJD8 | 1.259256 |
| STIM2 | 1.25901 |
| TMED5 | 1.258308 |
| PIGB | 1.254665 |
| DNJB9 | 1.250298 |
| CR3L2 | 1.249938 |
| SUMF2 | 1.248662 |
| GPC3 | 1.248572 |
| P3H1 | 1.248125 |
| RS3A | 1.245608 |
| DIK1B | 1.244253 |
| APLP2 | 1.241989 |
| COPA | 1.239326 |
| ATF6B | 1.234824 |
| RPN2 | 1.232567 |
| PXDN | 1.231671 |
| MFGM | 1.224066 |
| CDK1 | 1.220562 |
| TMEDA | 1.219797 |
| ELAV1 | 1.217685 |
| CO6A2 | 1.211701 |
| DPM1 | 1.209259 |
| PLOD3 | 1.207909 |
| RS8 | 1.207026 |
| SUMF1 | 1.205845 |
| CALX | 1.205115 |
| CLUS | 1.204325 |
| SOAT1 | 1.204264 |
| TMED4 | 1.203209 |
| CNPY3 | 1.201013 |
| RS23 | 1.199196 |
| NOMO3 | 1.194121 |
| RL27A | 1.191851 |
| FKB10 | 1.18971 |
| TMX3 | 1.189292 |
| COPD | 1.184732 |
| HS71B | 1.182981 |
| CALR | 1.176488 |
| CO5A1 | 1.173948 |
| PIGG | 1.167689 |
| TOIP2 | 1.162623 |
| ADA10 | 1.159204 |
| COPB | 1.157222 |
| PDIA1 | 1.156037 |
| EDEM2 | 1.153851 |
| PIGT | 1.153165 |
| FIBIN | 1.151179 |
| RL6 | 1.14906 |
| PIGU | 1.14611 |
| KPYM | 1.145265 |
| RCN1 | 1.14305 |
| AT131 | 1.141582 |
| NENF | 1.141477 |
| FKTN | 1.140739 |
| PPIB | 1.140291 |
| MOXD1 | 1.140118 |
| ERO1B | 1.138969 |
| RTN4R | 1.138771 |
| ERP29 | 1.138607 |
| AT2A2 | 1.136068 |
| RL36A | 1.13517 |
| PGRC2 | 1.134791 |
| ARSK | 1.134346 |
| PCSK6 | 1.128634 |
| COPG1 | 1.126369 |
| ITPR3 | 1.126123 |
| TMTC3 | 1.125655 |
| TM39A | 1.125195 |
| P4HA2 | 1.124161 |
| TMX1 | 1.120684 |
| LMAN2 | 1.11944 |
| PDIA6 | 1.11831 |
| EXT1 | 1.115371 |
| PRIO | 1.11412 |
| GLCM | 1.112892 |
| ERP44 | 1.110865 |
| RS26 | 1.109777 |
| S61A1 | 1.109457 |
| TMM33 | 1.106272 |
| NF2L1 | 1.105176 |
| CO6A1 | 1.104262 |
| UBAC2 | 1.104162 |
| TRFM | 1.104032 |
| SULF1 | 1.100147 |
| DJC10 | 1.09949 |
| AT1A1 | 1.098702 |
| LMF2 | 1.09466 |
| DPA1 | 1.086159 |
| CD59 | 1.084177 |
| HACD3 | 1.078648 |
| A4 | 1.07494 |
| SIL1 | 1.071262 |
| RCN2 | 1.071103 |
| TXD16 | 1.070857 |
| HSP13 | 1.069977 |
| CGT | 1.06915 |
| NPC1 | 1.068854 |
| RL34 | 1.065038 |
| ENPL | 1.060811 |
| P4HA1 | 1.060572 |
| ERAP2 | 1.059132 |
| ERAP1 | 1.057638 |
| TOR1B | 1.055432 |
| RL10 | 1.054664 |
| FUCO2 | 1.052772 |
| 15-Sep | 1.048756 |
| SC65 | 1.048439 |
| SURF4 | 1.047599 |
| PGLT1 | 1.046355 |
| UCHL1 | 1.046065 |
| BAP29 | 1.037268 |
| MANF | 1.036421 |
| RL4 | 1.0314 |
| PLGT2 | 1.03043 |
| SELT | 1.030032 |
| CLCC1 | 1.020494 |
| PIGO | 1.019233 |
| ACATN | 1.014696 |
| MA1A2 | 1.013303 |
| BIP | 1.010312 |
| ULBP1 | 1.009523 |
| MBTP1 | 1.008131 |
| TECR | 1.007993 |
| 1C07 | 1.005788 |
| T106C | 1.001361 |
| SULF2 | 0.995742 |
| COIA1 | 0.995425 |
| ARSA | 0.993869 |
| SE1L1 | 0.993169 |
| ERN1 | 0.988907 |
| EXT2 | 0.975318 |
| MBRL | 0.971625 |
| AHSA1 | 0.966063 |
| GPI8 | 0.965495 |
| S35B2 | 0.962174 |
| P3H2 | 0.960813 |
| LRC8E | 0.956984 |
| IMB1 | 0.955198 |
| DAD1 | 0.941334 |
| ERLEC | 0.941243 |
| PLOD2 | 0.940076 |
| TXND5 | 0.939253 |
| ERGI3 | 0.937455 |
| PDIA5 | 0.932606 |
| UGGG2 | 0.929918 |
| TXD11 | 0.920884 |
| PLOD1 | 0.918357 |
| OS9 | 0.917813 |
| CLGN | 0.911516 |
| MOGS | 0.907698 |
| PLGT3 | 0.893556 |
| SRPRA | 0.893305 |
| FKBP7 | 0.891913 |
| GALT2 | 0.891618 |
| EDEM1 | 0.885389 |
| CCD47 | 0.871505 |
| EST2 | 0.867913 |
| ERO1A | 0.866643 |
| HLAF | 0.860879 |
| ERLN2 | 0.852221 |
| XXLT1 | 0.847535 |
| POMT1 | 0.846703 |
| CH60 | 0.84512 |
| ARSG | 0.843417 |
| GT251 | 0.843389 |
| POMT2 | 0.841584 |
| DJB14 | 0.83568 |
| ERLN1 | 0.834825 |
| OFUT1 | 0.8275 |
| CAB45 | 0.827491 |
| GT253 | 0.817105 |
| EDEM3 | 0.813072 |
| EXTL3 | 0.811852 |
| CHLE | 0.808255 |
| PLD3 | 0.805539 |
| PGAP1 | 0.799364 |
| UGGG1 | 0.798301 |
| OFUT2 | 0.788281 |
| SL9A6 | 0.777076 |
| FXRD2 | 0.753304 |
| ILVBL | 0.739095 |
| NUCB2 | 0.736545 |
| PMGT2 | 0.711331 |
| LMA2L | 0.709674 |
| CALU | 0.707862 |
| LMF1 | 0.687462 |
| SC11C | 0.666577 |
| LRC8C | 0.647521 |
| SC11A | 0.629922 |
| SG196 | 0.62387 |
| TOR1A | 0.593706 |
| ARSE | 0.559128 |
| TPST2 | 0.559027 |
| PRDX4 | 0.518189 |
| CNPY2 | 0.481763 |
