# Supplementary figures and images for: Accumulated precursors of specific GPI-anchored proteins upregulate GPI biosynthesis with ARV1
Source: J Cell Biol. 2023 Feb 24;222(5):e202208159. doi: 10.1083/jcb.202208159 (PMC9997660; doi:10.1083/jcb.202208159)

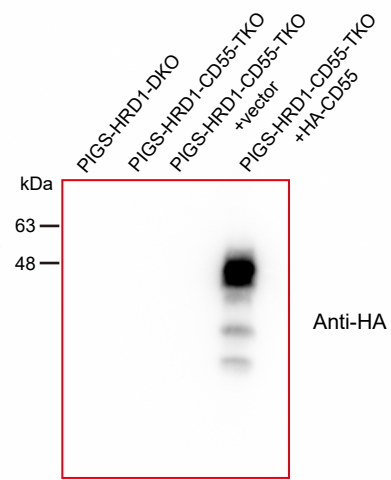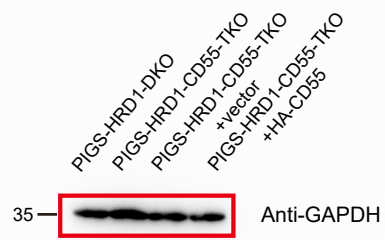

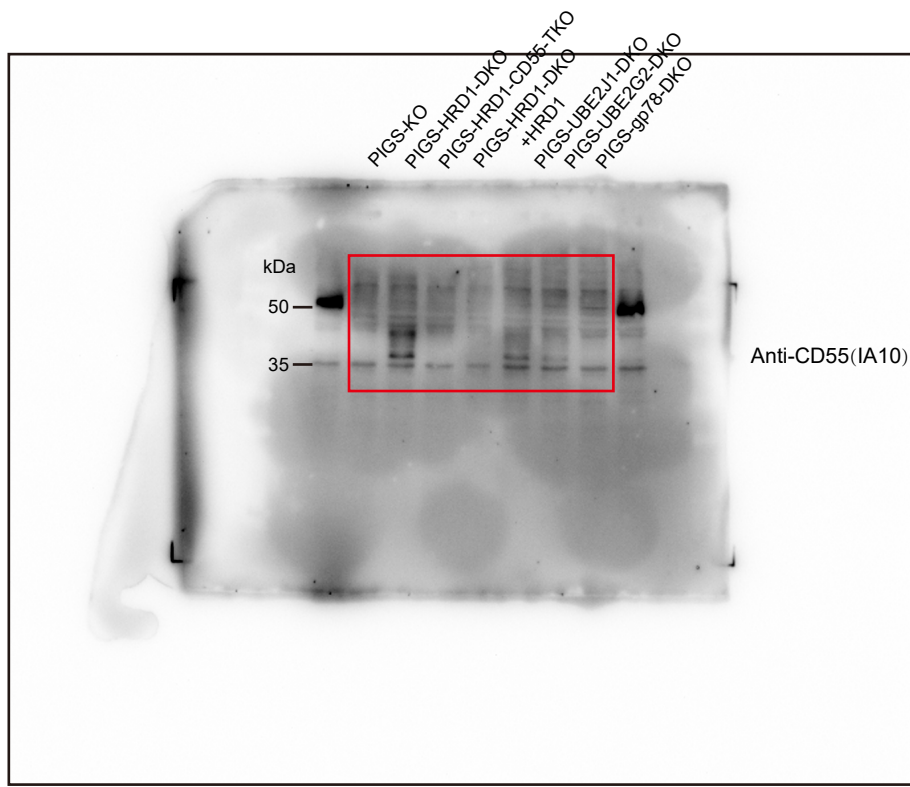

PIGS-KO  
PIGS-HRD1-DKO  
PIGS-HRD1-DKO  
+HRD1

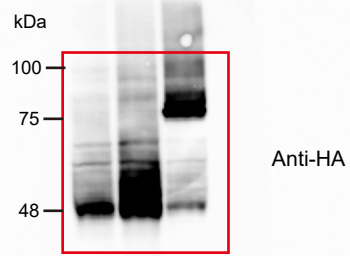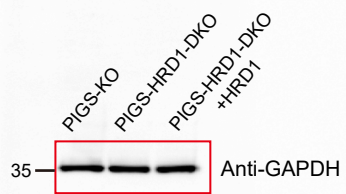

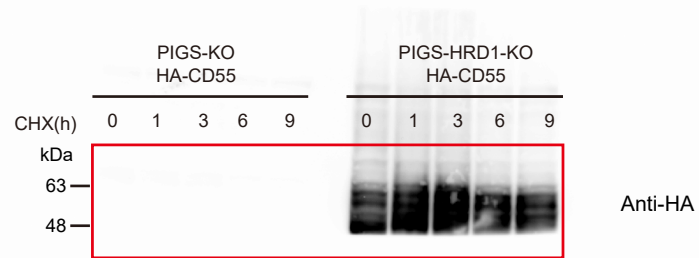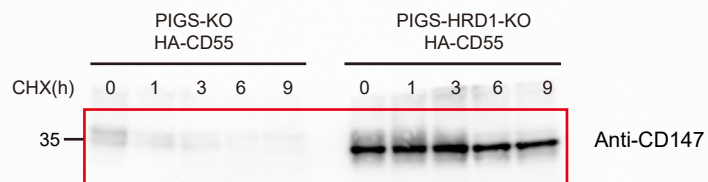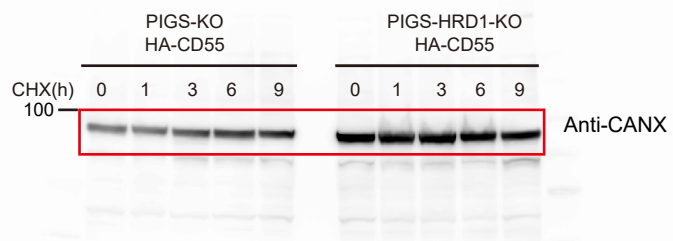

Supplement: SourceData F3 — is the source file for Fig. 3. [file JCB_202208159_SourceDataF3.pdf]

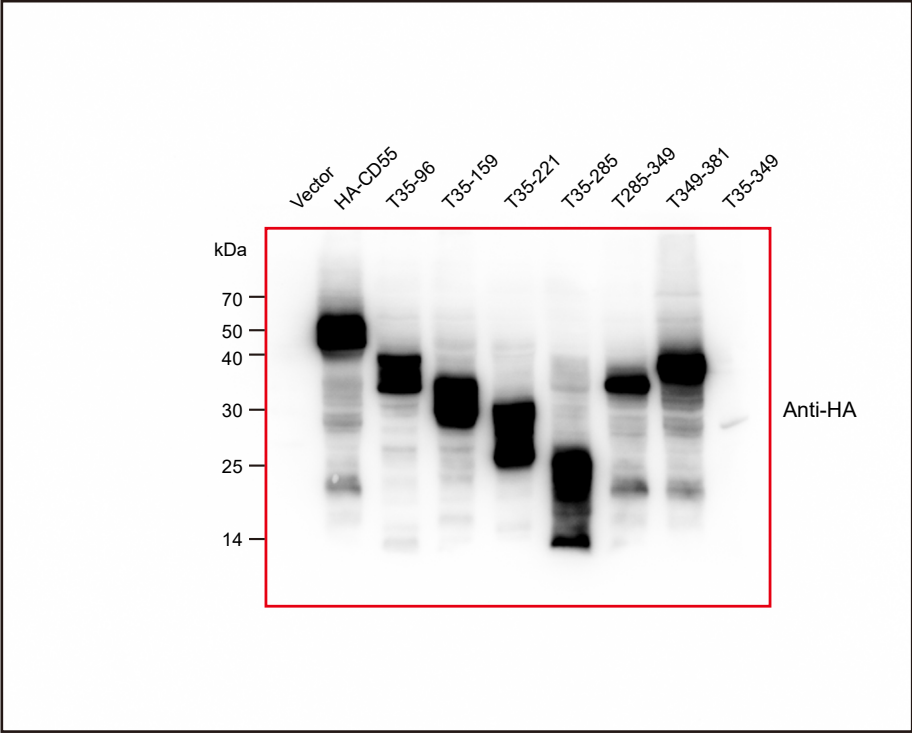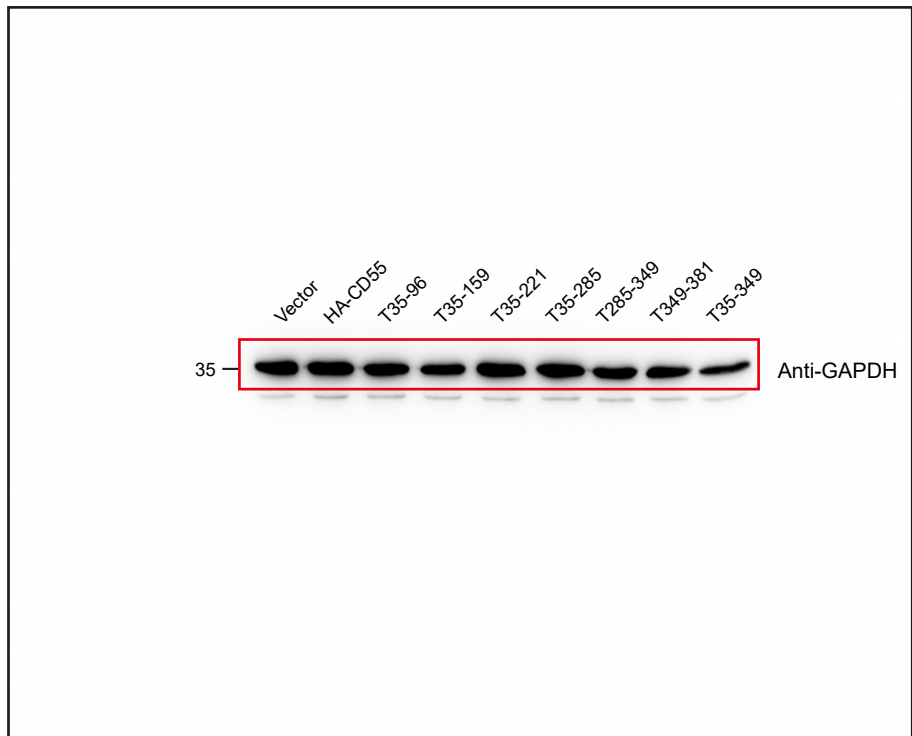

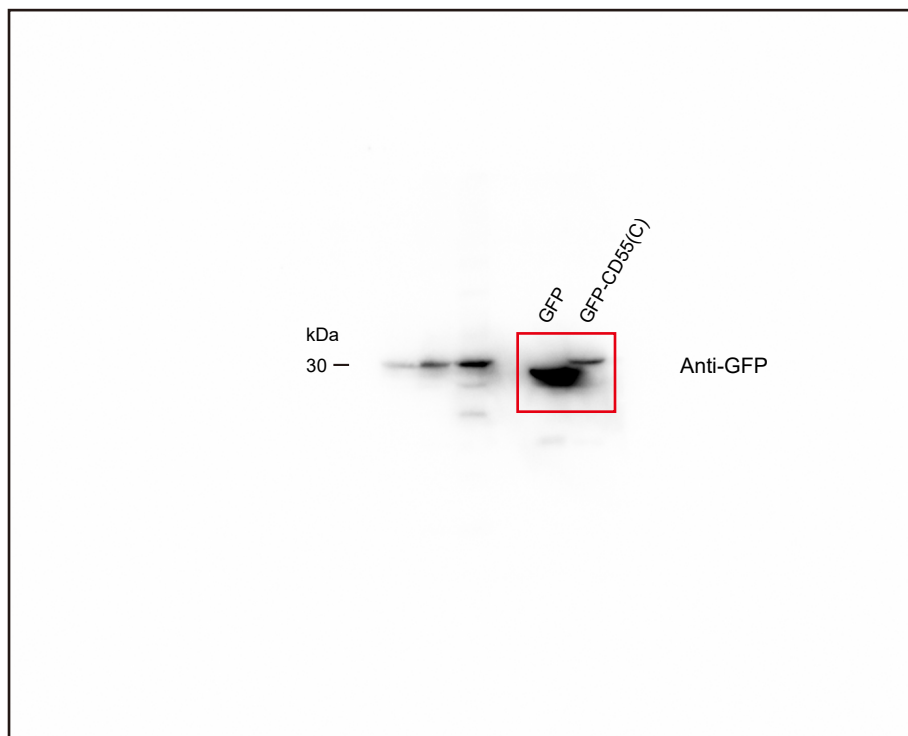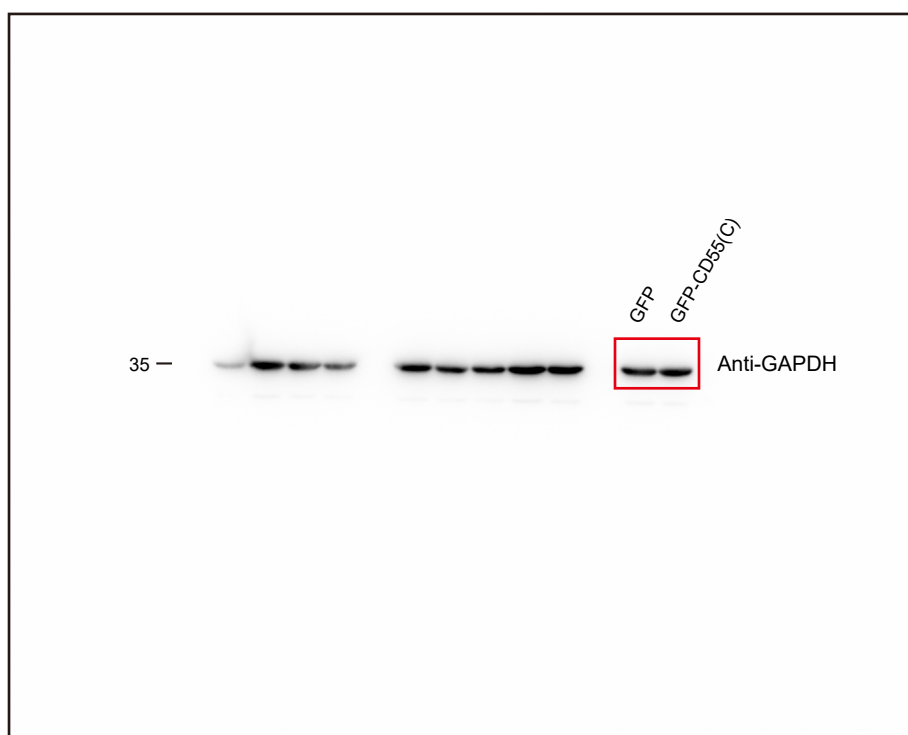

Supplement: SourceData F4 — is the source file for Fig. 4. [file JCB_202208159_SourceDataF4.pdf]

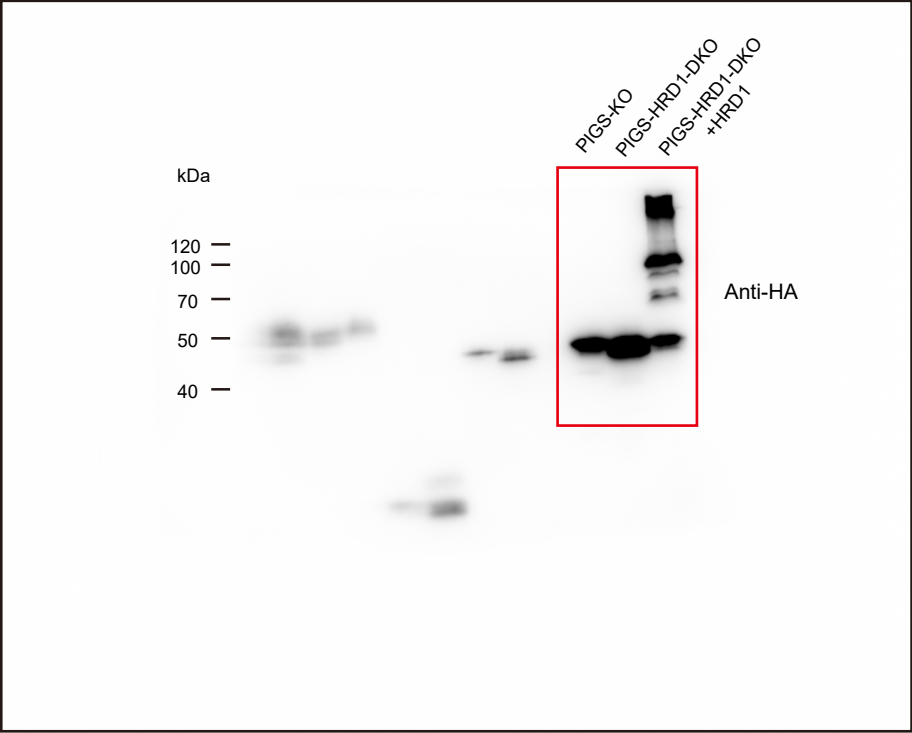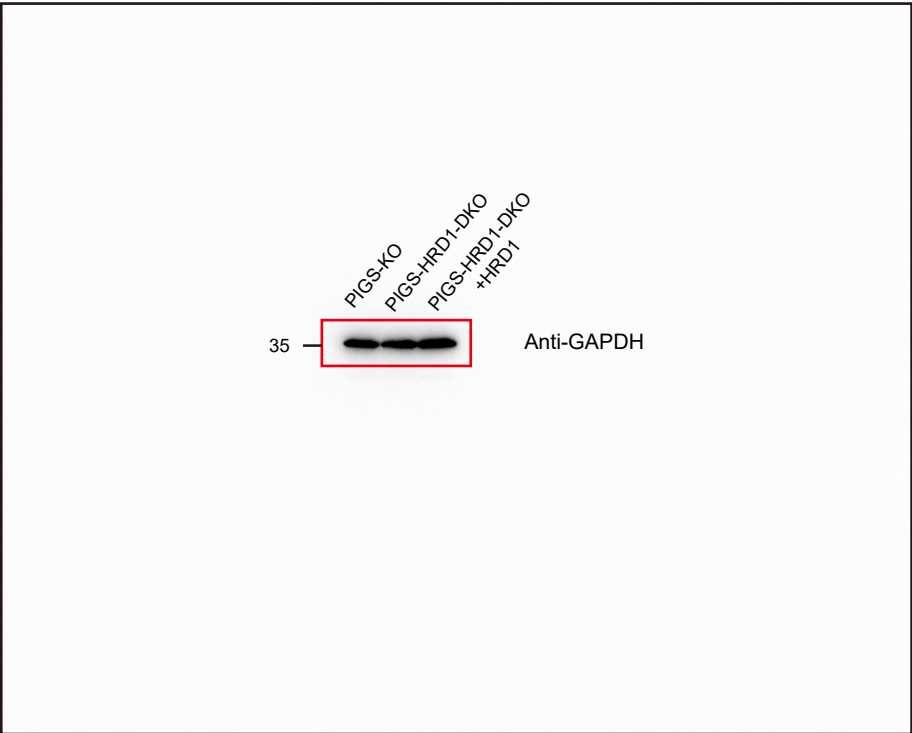

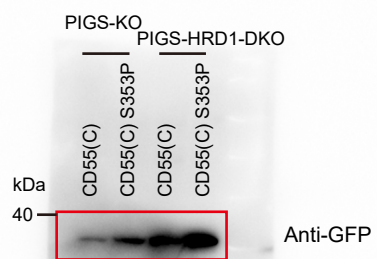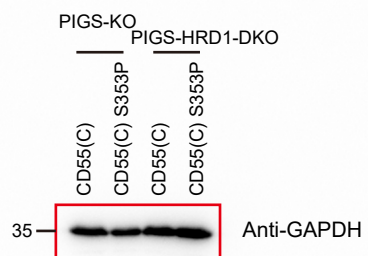

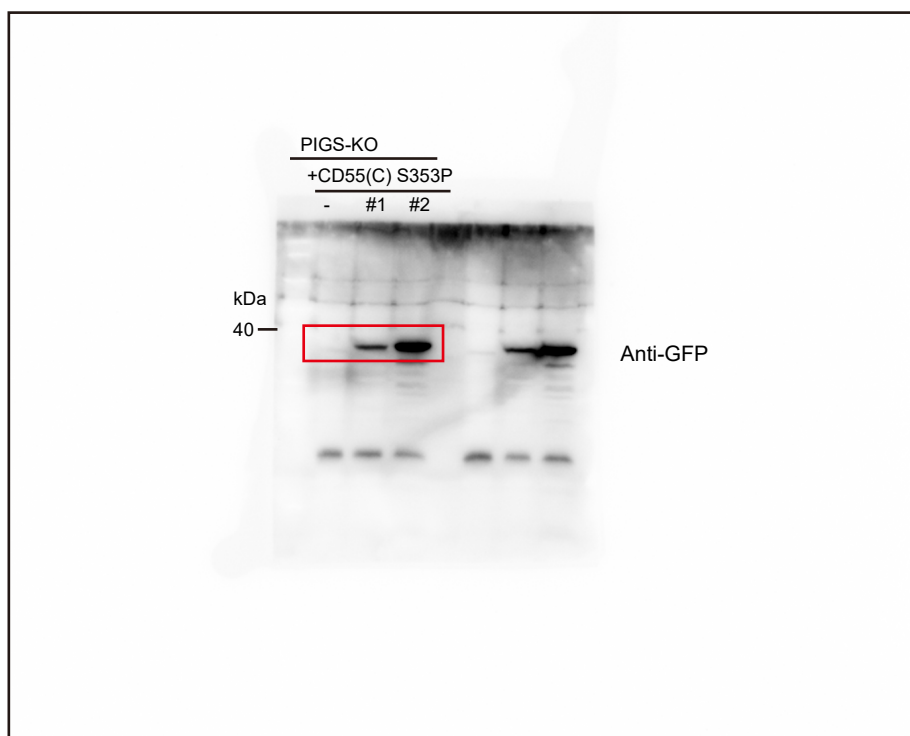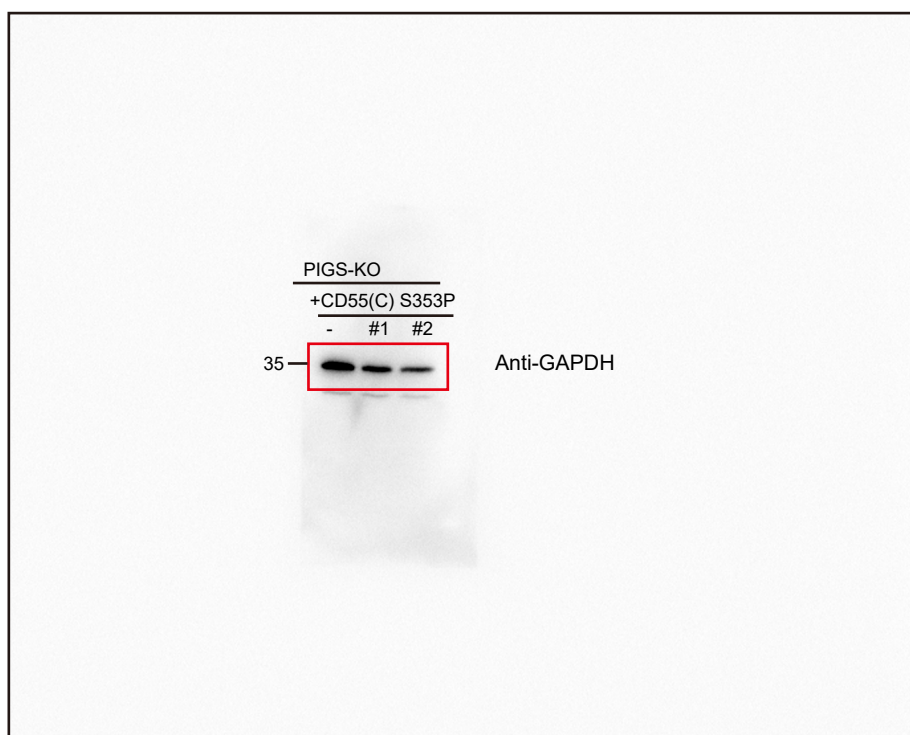

Supplement: SourceData F5 — is the source file for Fig. 5. [file JCB_202208159_SourceDataF5.pdf]

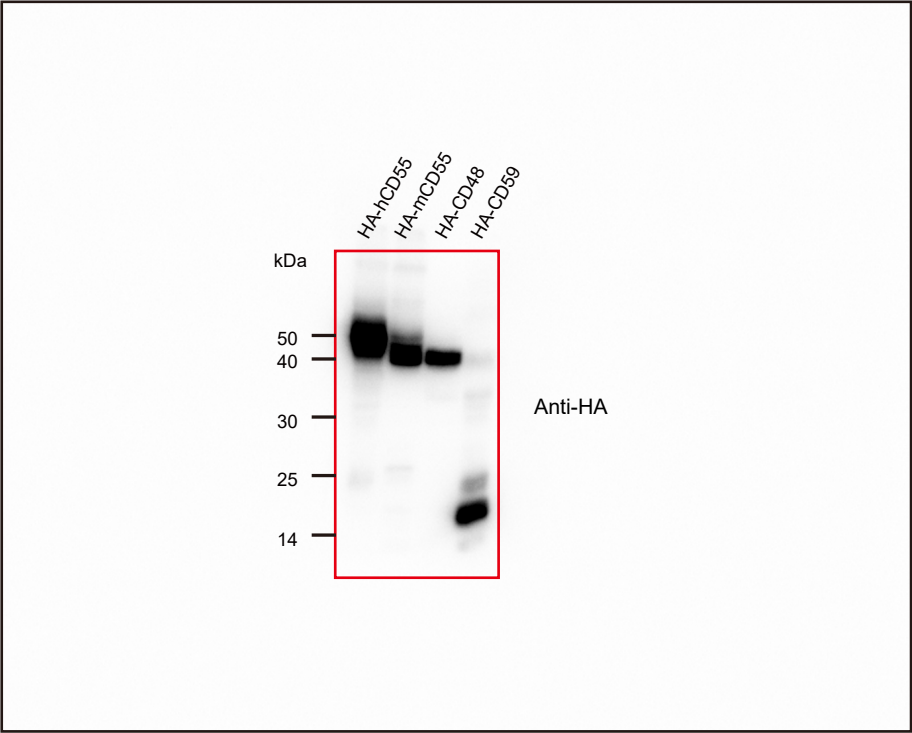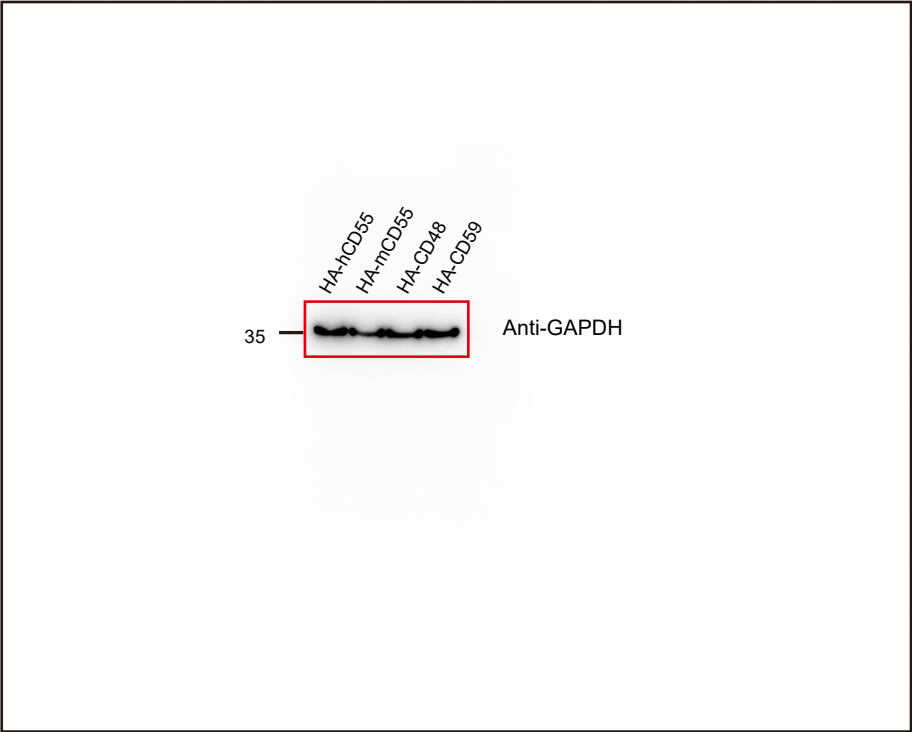

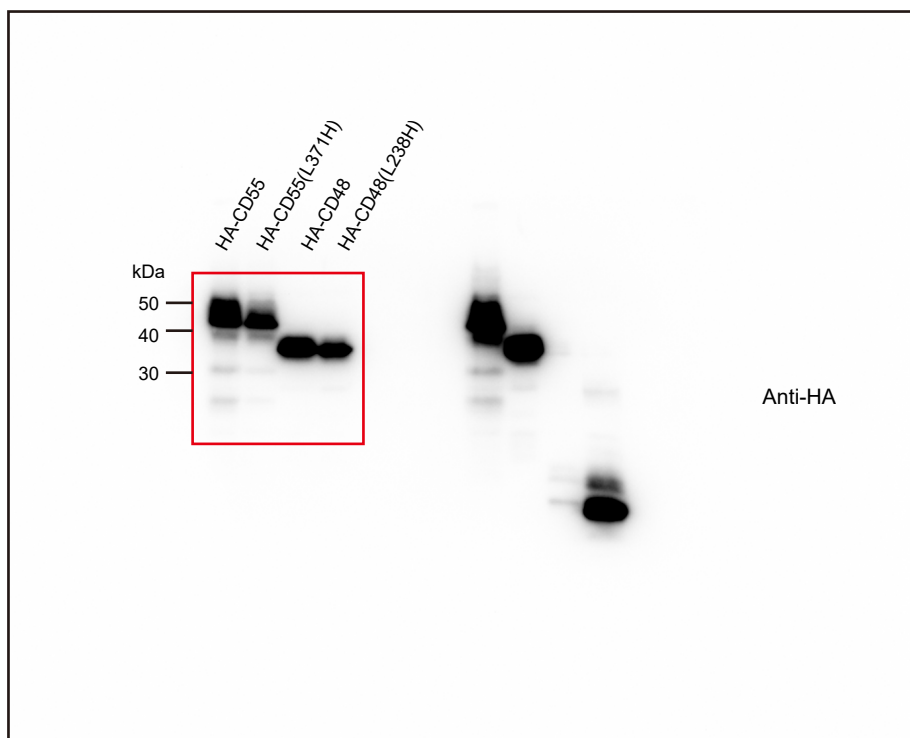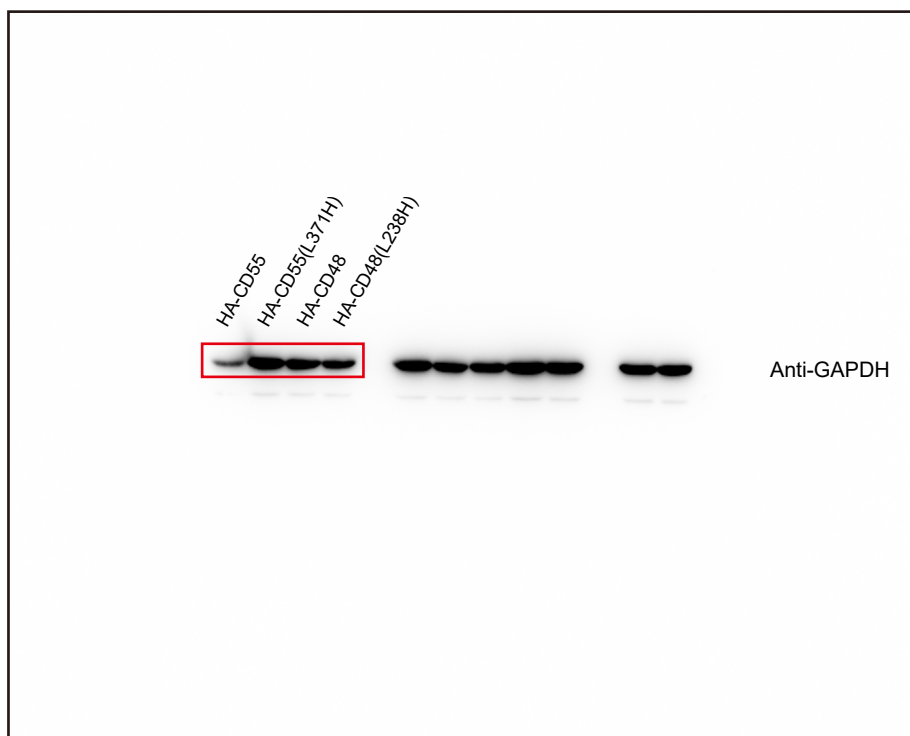

Supplement: SourceData F6 — is the source file for Fig. 6. [file JCB_202208159_SourceDataF6.pdf]

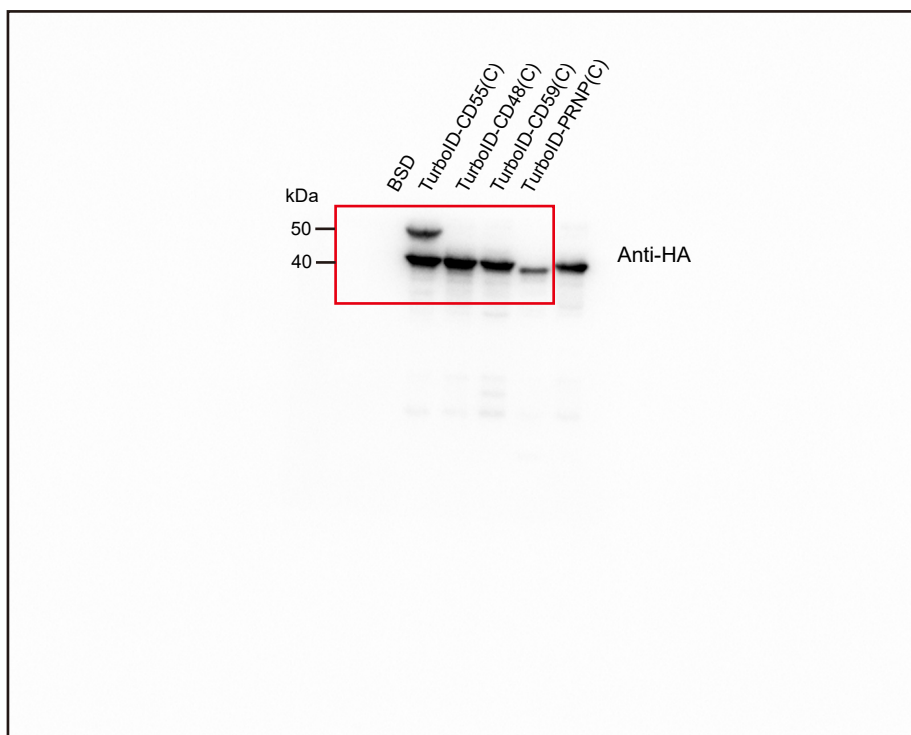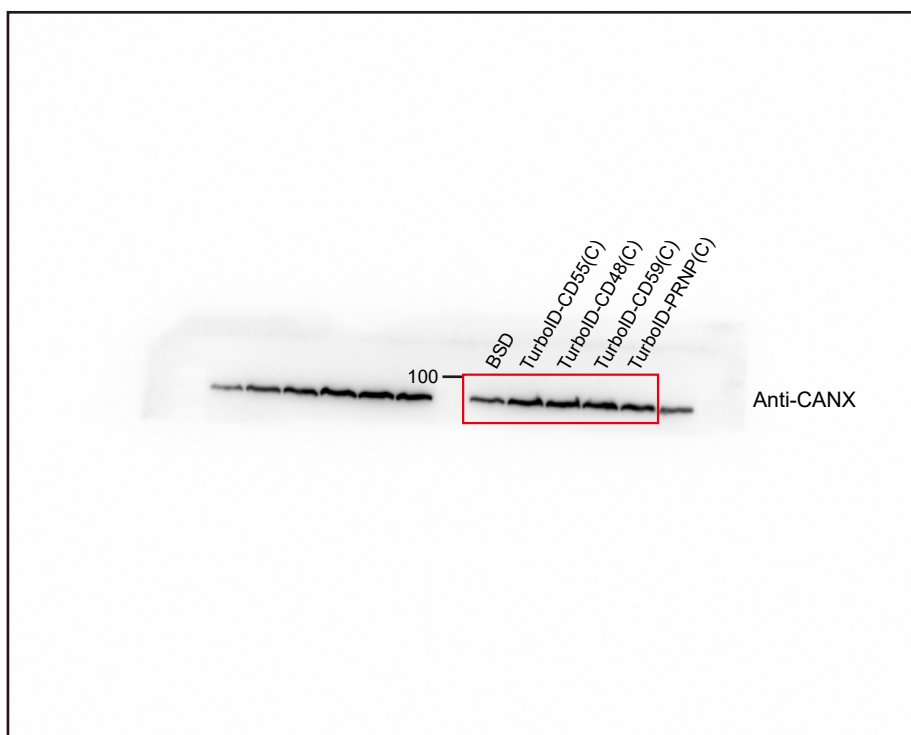

Supplement: SourceData F7 — is the source file for Fig. 7. [file JCB_202208159_SourceDataF7.pdf]

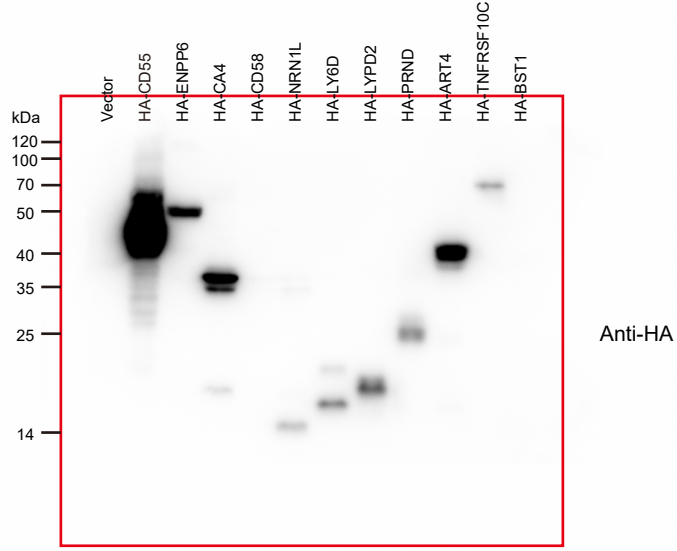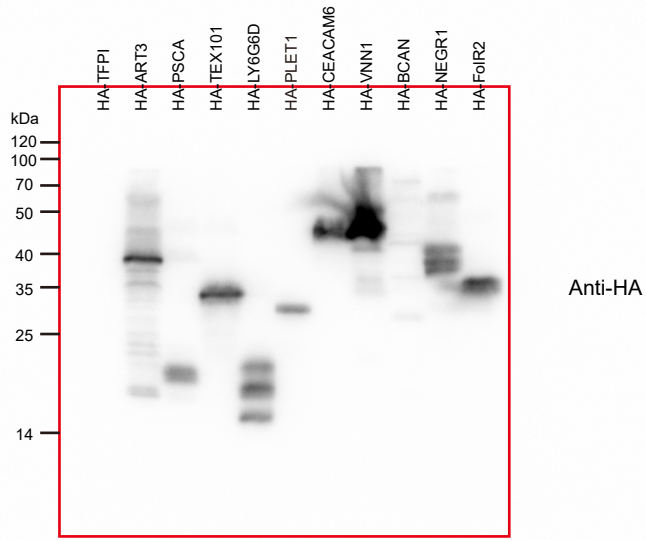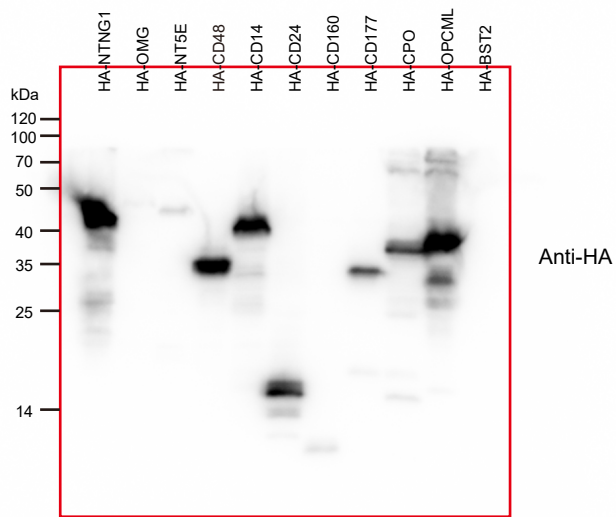

Supplement: SourceData FS3 — is the source file for Fig. S3. [file JCB_202208159_SourceDataFS3.pdf]

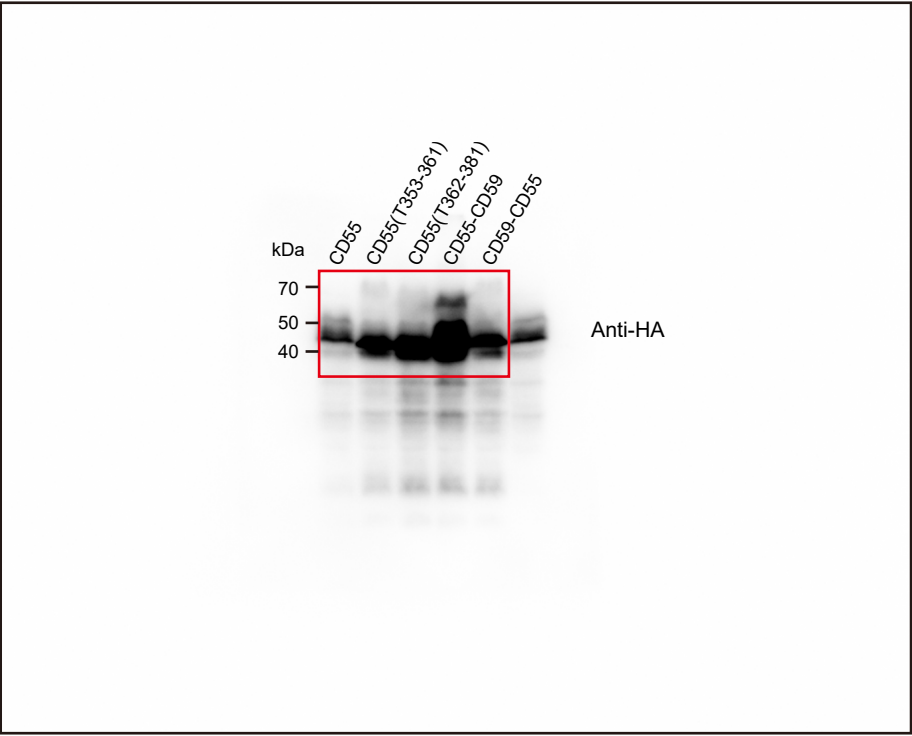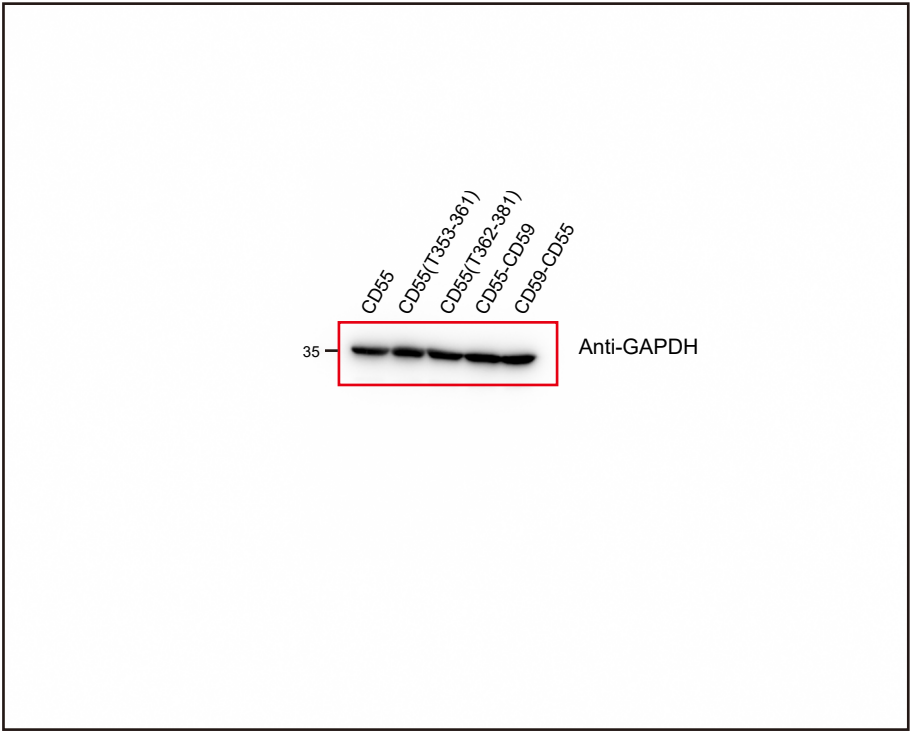

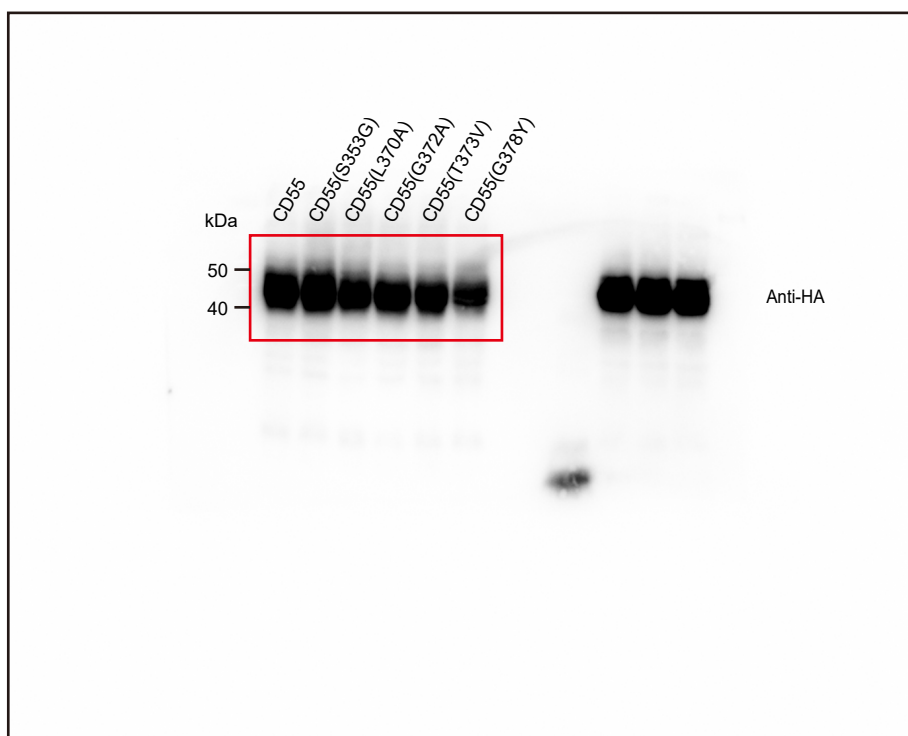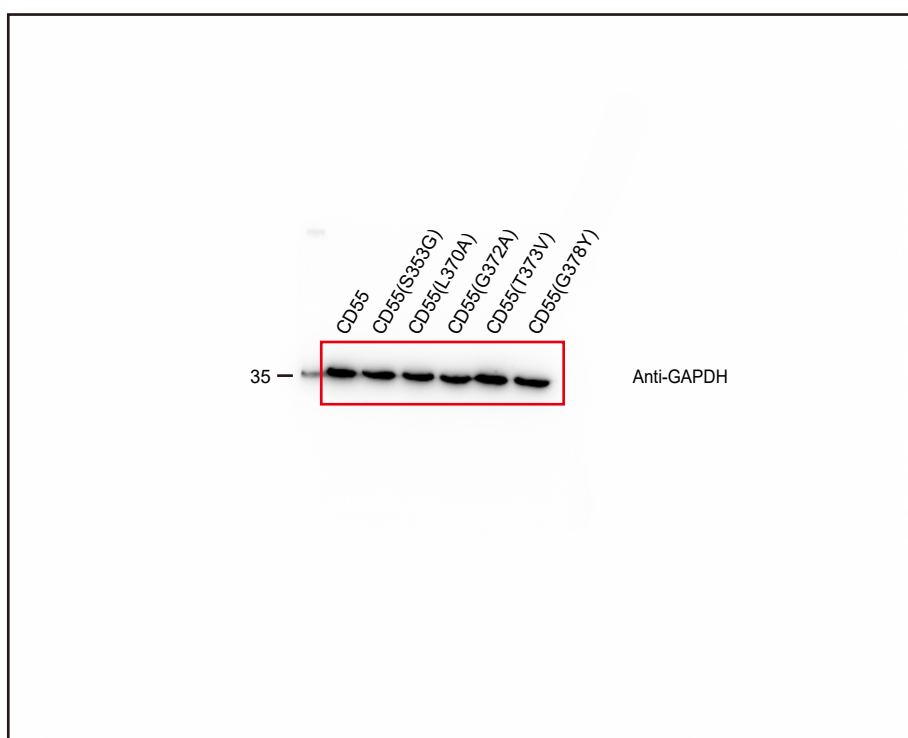

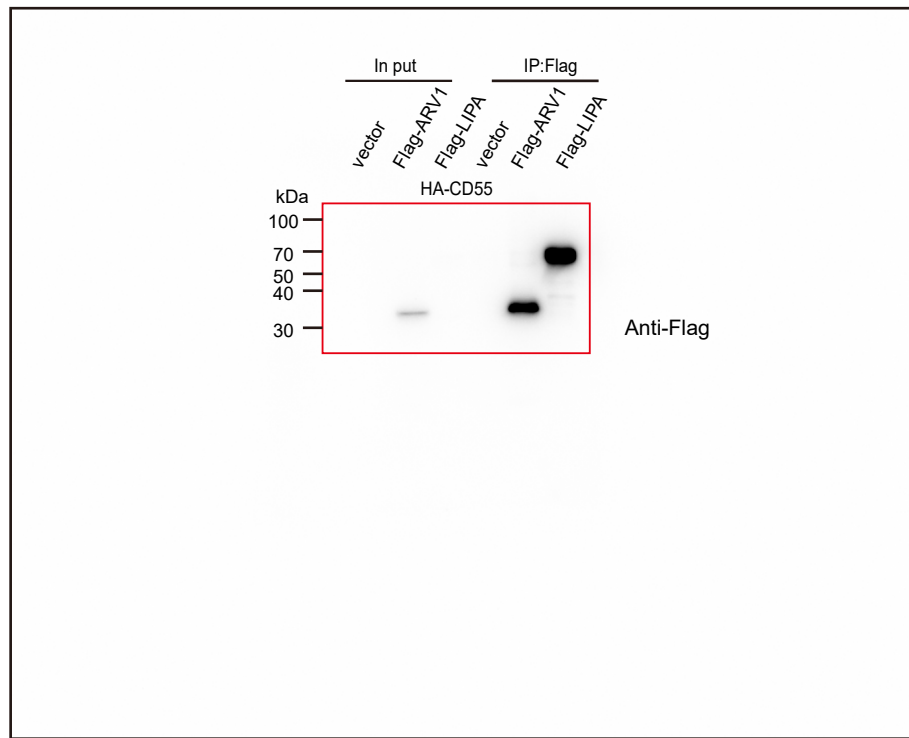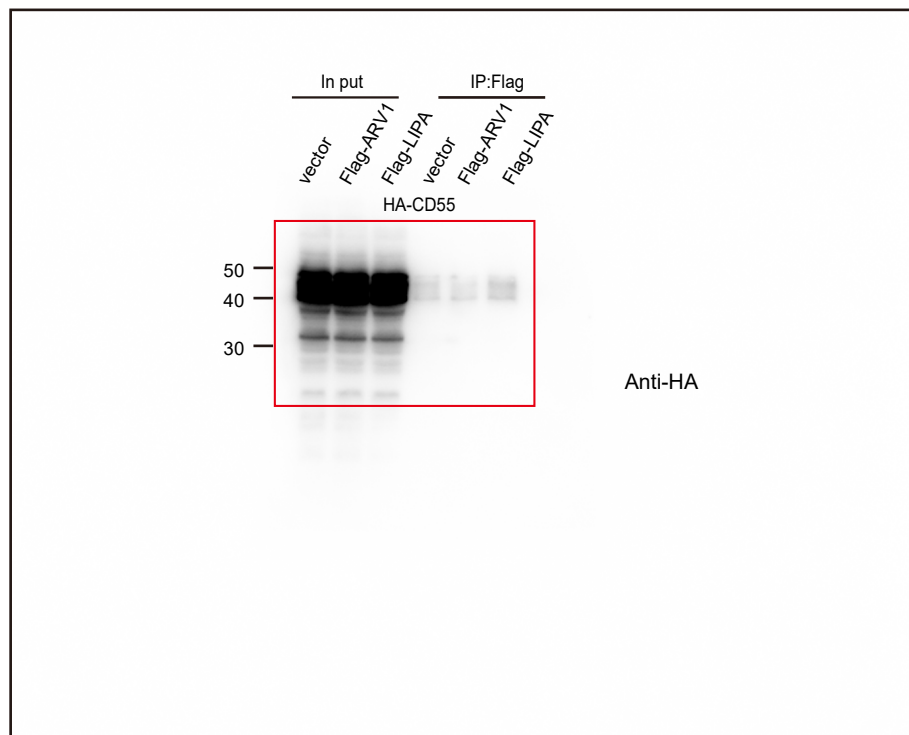

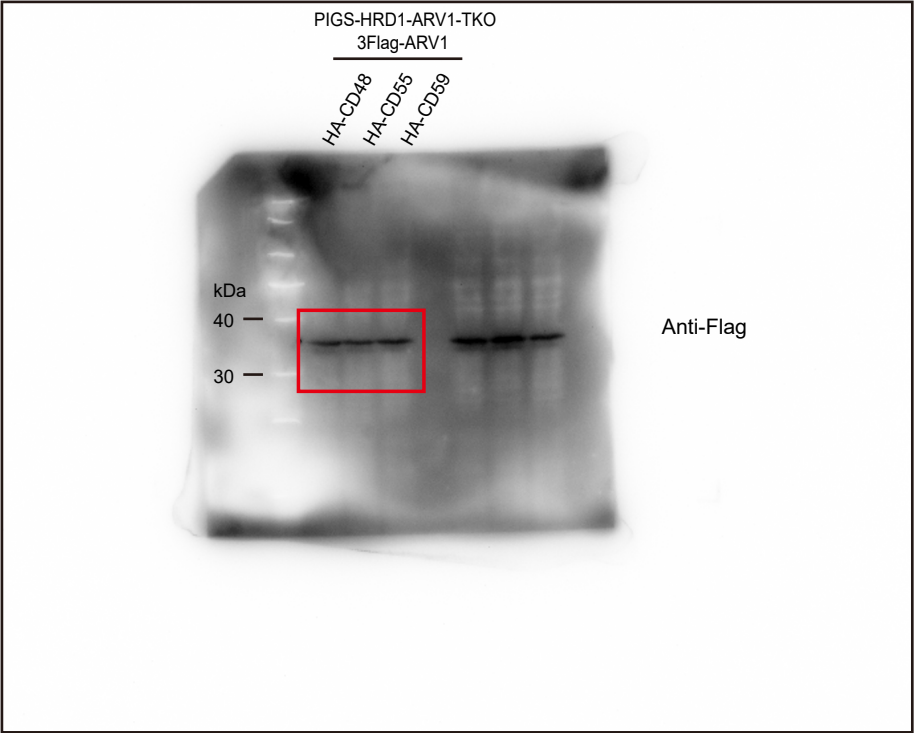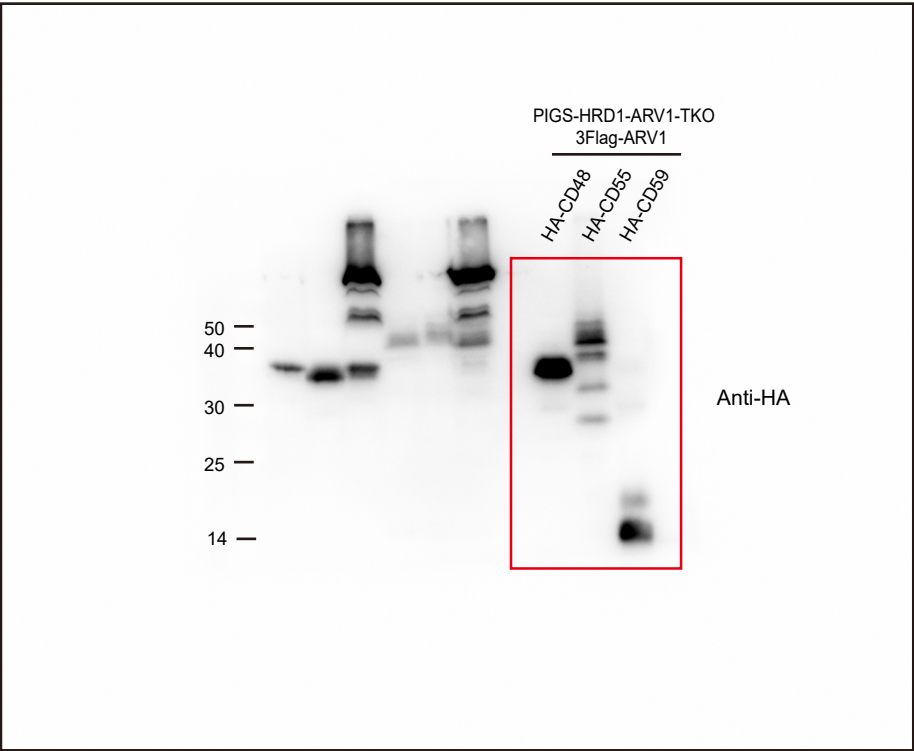

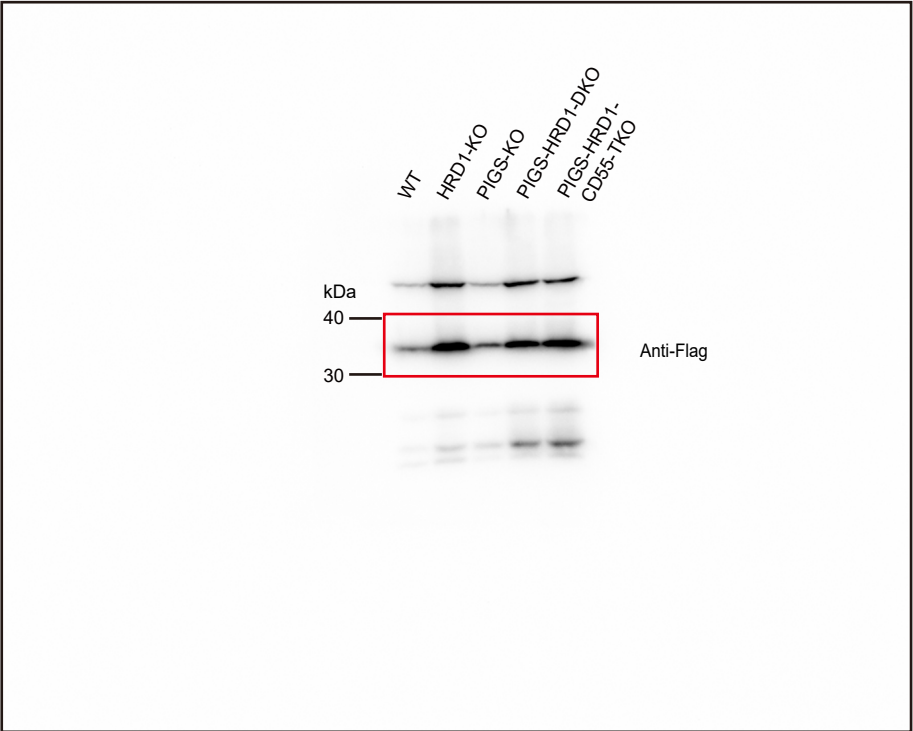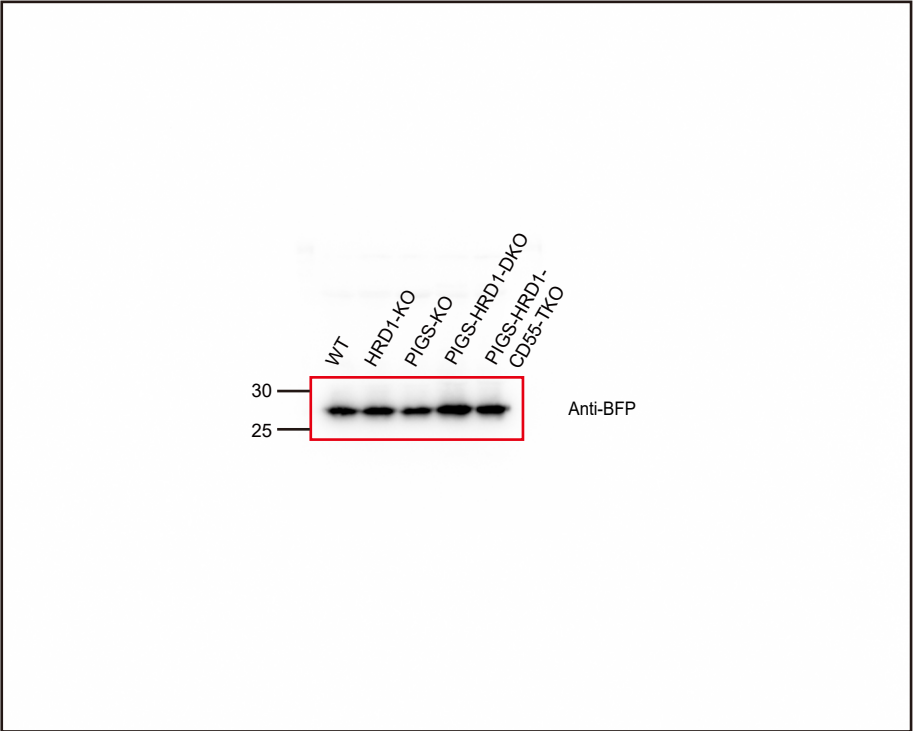

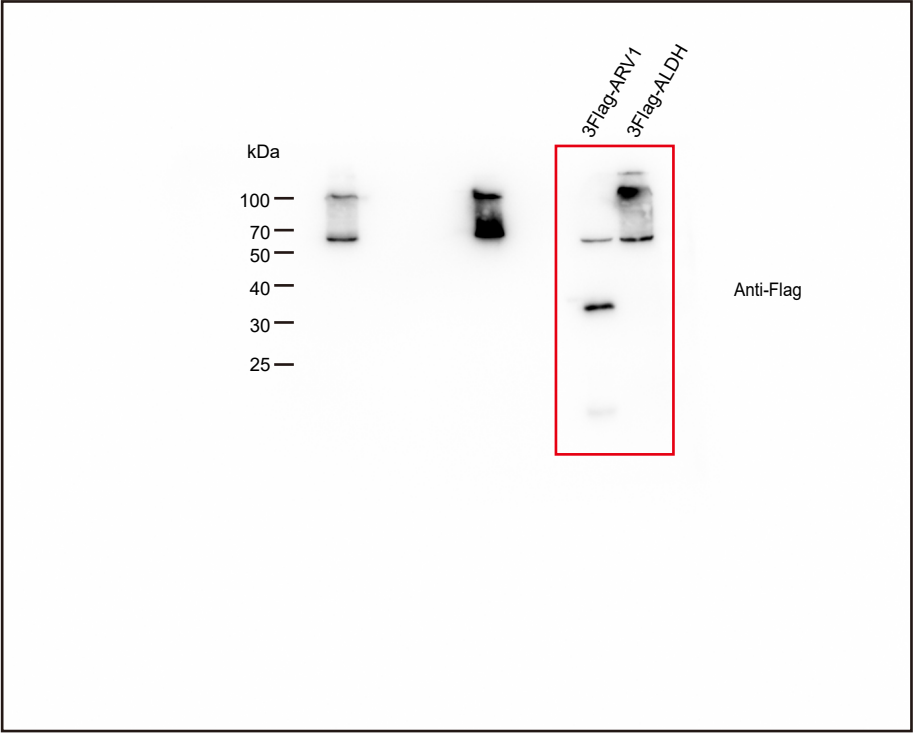

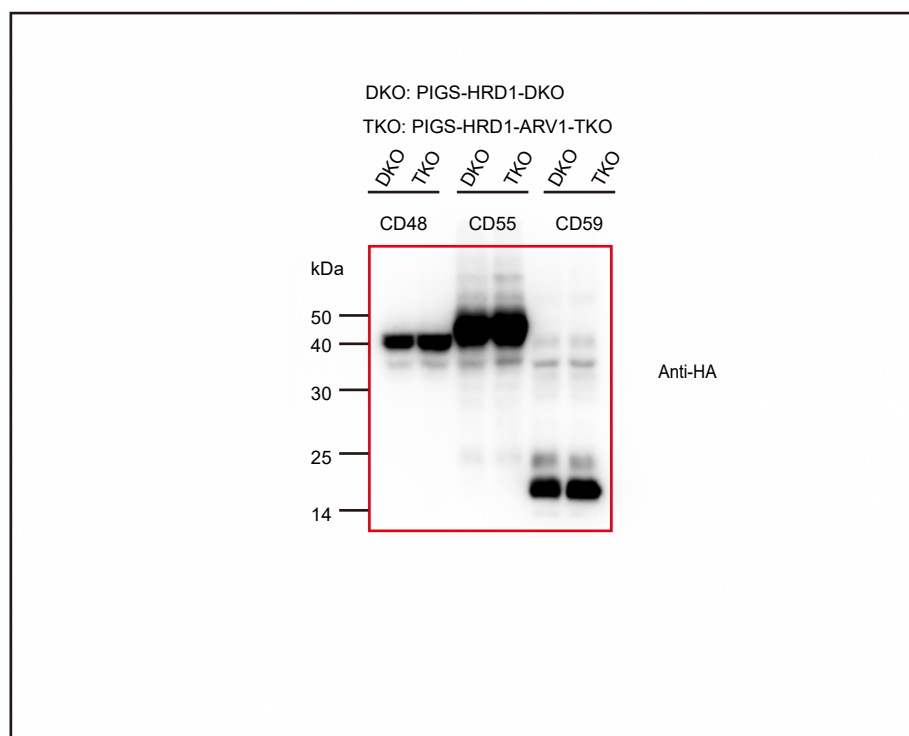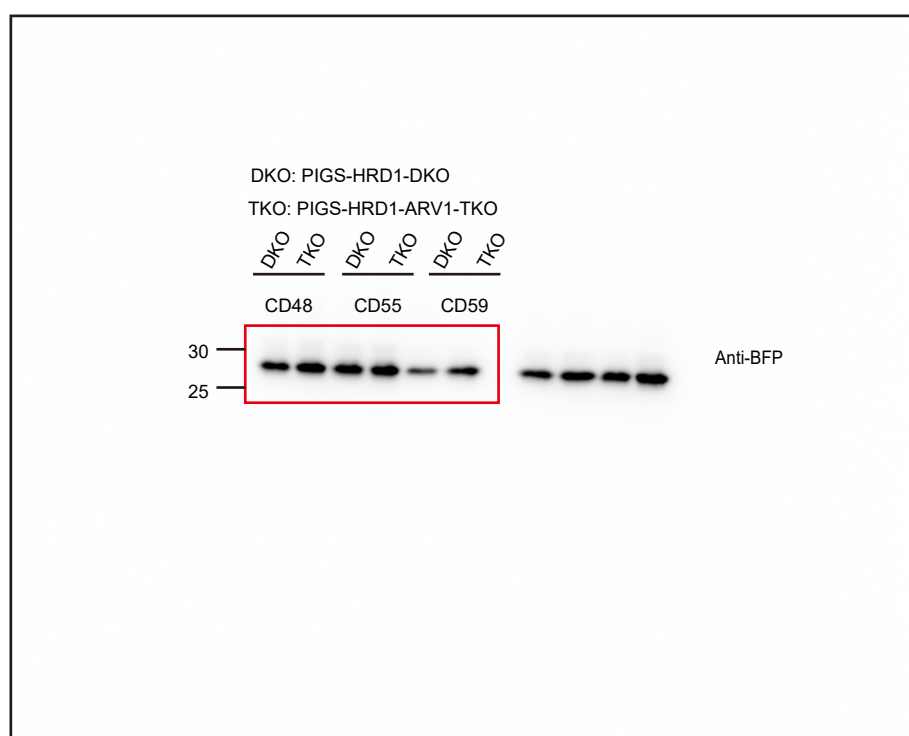

Supplement: SourceData FS4 — is the source file for Fig. S4. [file JCB_202208159_SourceDataFS4.pdf]

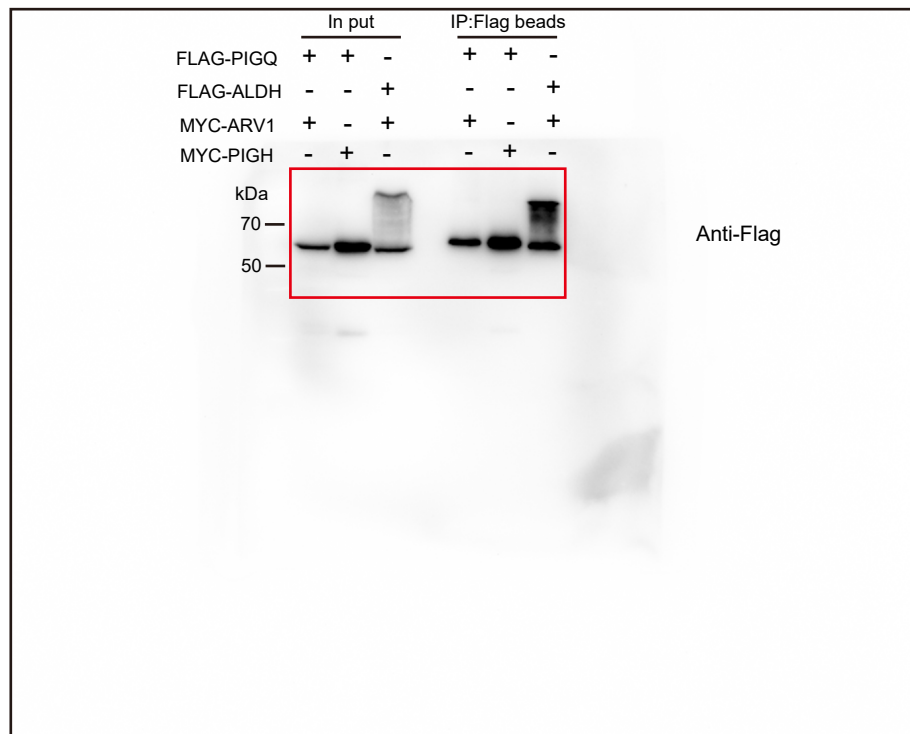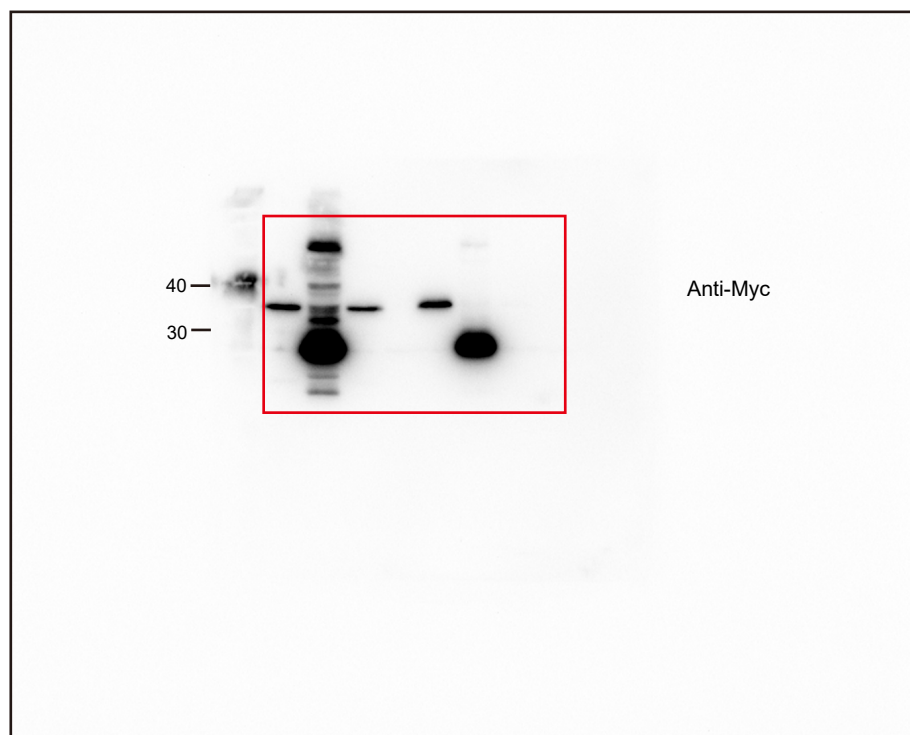

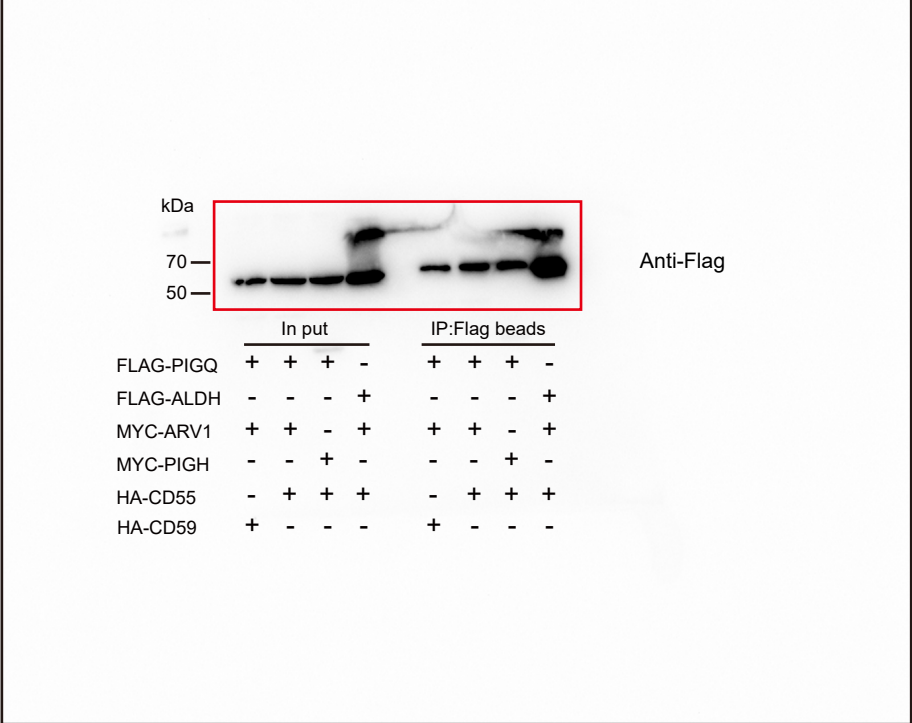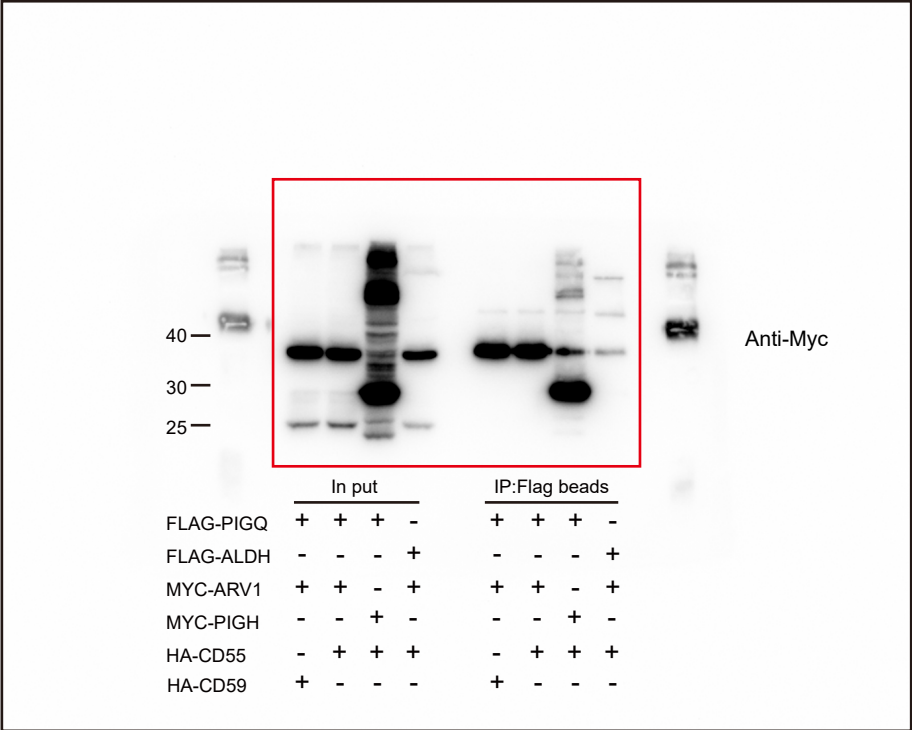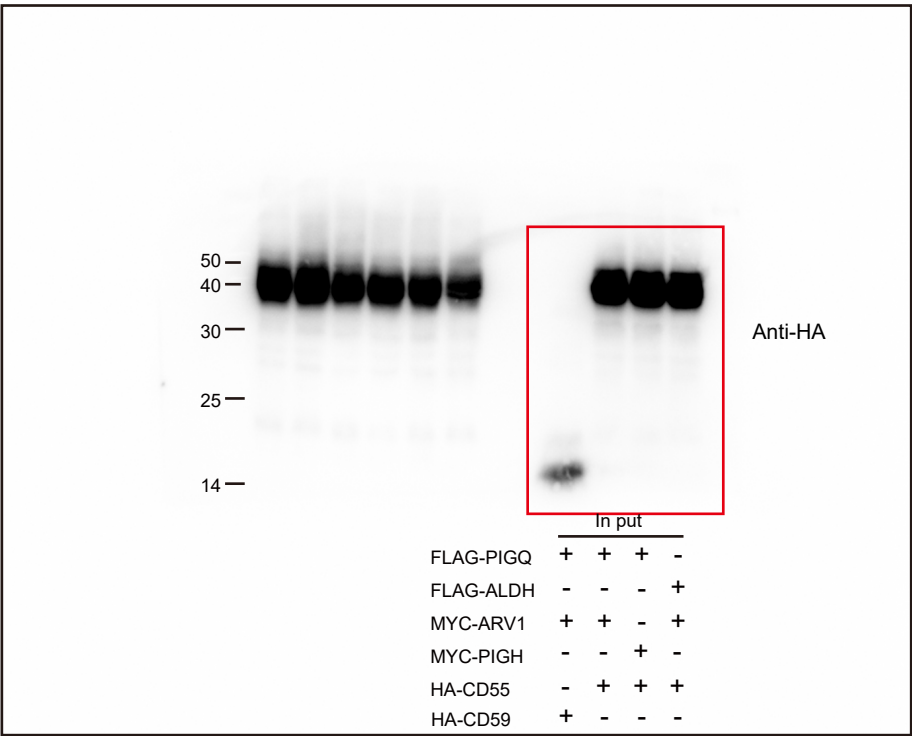

Supplement: SourceData FS5 — is the source file for Fig. S5. [file JCB_202208159_SourceDataFS5.pdf]
